# A microbial gene catalog of anaerobic digestion from full-scale biogas plants

**DOI:** 10.1093/gigascience/giaa164

**Published:** 2021-01-27

**Authors:** Shichun Ma, Fan Jiang, Yan Huang, Yan Zhang, Sen Wang, Hui Fan, Bo Liu, Qiang Li, Lijuan Yin, Hengchao Wang, Hangwei Liu, Yuwei Ren, Shuqu Li, Lei Cheng, Wei Fan, Yu Deng

**Affiliations:** Biogas Institute of Ministry of Agricultural and Rural Affairs, Section 4-13, Renmin South Road, Chengdu 610041, China; Laboratory of Development and Application of Rural Renewable Energy, Ministry of Agricultural and Rural Affairs, Section 4-13, Renmin South Road, Chengdu 610041, China; Guangdong Laboratory for Lingnan Modern Agriculture (Shenzhen Branch), Genome Analysis Laboratory of the Ministry of Agriculture and Rural Affairs, Agricultural Genomics Institute at Shenzhen, Chinese Academy of Agricultural Sciences, 7 Pengfai Road, Shenzhen 518120,China; Biogas Institute of Ministry of Agricultural and Rural Affairs, Section 4-13, Renmin South Road, Chengdu 610041, China; Laboratory of Development and Application of Rural Renewable Energy, Ministry of Agricultural and Rural Affairs, Section 4-13, Renmin South Road, Chengdu 610041, China; Guangdong Laboratory for Lingnan Modern Agriculture (Shenzhen Branch), Genome Analysis Laboratory of the Ministry of Agriculture and Rural Affairs, Agricultural Genomics Institute at Shenzhen, Chinese Academy of Agricultural Sciences, 7 Pengfai Road, Shenzhen 518120, China; Guangdong Laboratory for Lingnan Modern Agriculture (Shenzhen Branch), Genome Analysis Laboratory of the Ministry of Agriculture and Rural Affairs, Agricultural Genomics Institute at Shenzhen, Chinese Academy of Agricultural Sciences, 7 Pengfai Road, Shenzhen 518120, China; Biogas Institute of Ministry of Agricultural and Rural Affairs, Section 4-13, Renmin South Road, Chengdu 610041, China; Laboratory of Development and Application of Rural Renewable Energy, Ministry of Agricultural and Rural Affairs, Section 4-13, Renmin South Road, Chengdu 610041, China; Guangdong Laboratory for Lingnan Modern Agriculture (Shenzhen Branch), Genome Analysis Laboratory of the Ministry of Agriculture and Rural Affairs, Agricultural Genomics Institute at Shenzhen, Chinese Academy of Agricultural Sciences, 7 Pengfai Road, Shenzhen 518120, China; Biogas Institute of Ministry of Agricultural and Rural Affairs, Section 4-13, Renmin South Road, Chengdu 610041, China; Laboratory of Development and Application of Rural Renewable Energy, Ministry of Agricultural and Rural Affairs, Section 4-13, Renmin South Road, Chengdu 610041, China; Guangdong Laboratory for Lingnan Modern Agriculture (Shenzhen Branch), Genome Analysis Laboratory of the Ministry of Agriculture and Rural Affairs, Agricultural Genomics Institute at Shenzhen, Chinese Academy of Agricultural Sciences, 7 Pengfai Road, Shenzhen 518120, China; Guangdong Laboratory for Lingnan Modern Agriculture (Shenzhen Branch), Genome Analysis Laboratory of the Ministry of Agriculture and Rural Affairs, Agricultural Genomics Institute at Shenzhen, Chinese Academy of Agricultural Sciences, 7 Pengfai Road, Shenzhen 518120, China; Guangdong Laboratory for Lingnan Modern Agriculture (Shenzhen Branch), Genome Analysis Laboratory of the Ministry of Agriculture and Rural Affairs, Agricultural Genomics Institute at Shenzhen, Chinese Academy of Agricultural Sciences, 7 Pengfai Road, Shenzhen 518120, China; Guangdong Laboratory for Lingnan Modern Agriculture (Shenzhen Branch), Genome Analysis Laboratory of the Ministry of Agriculture and Rural Affairs, Agricultural Genomics Institute at Shenzhen, Chinese Academy of Agricultural Sciences, 7 Pengfai Road, Shenzhen 518120, China; Guangdong Laboratory for Lingnan Modern Agriculture (Shenzhen Branch), Genome Analysis Laboratory of the Ministry of Agriculture and Rural Affairs, Agricultural Genomics Institute at Shenzhen, Chinese Academy of Agricultural Sciences, 7 Pengfai Road, Shenzhen 518120, China; Biogas Institute of Ministry of Agricultural and Rural Affairs, Section 4-13, Renmin South Road, Chengdu 610041, China; Laboratory of Development and Application of Rural Renewable Energy, Ministry of Agricultural and Rural Affairs, Section 4-13, Renmin South Road, Chengdu 610041, China; Guangdong Laboratory for Lingnan Modern Agriculture (Shenzhen Branch), Genome Analysis Laboratory of the Ministry of Agriculture and Rural Affairs, Agricultural Genomics Institute at Shenzhen, Chinese Academy of Agricultural Sciences, 7 Pengfai Road, Shenzhen 518120, China; Biogas Institute of Ministry of Agricultural and Rural Affairs, Section 4-13, Renmin South Road, Chengdu 610041, China; Laboratory of Development and Application of Rural Renewable Energy, Ministry of Agricultural and Rural Affairs, Section 4-13, Renmin South Road, Chengdu 610041, China

**Keywords:** anaerobic digestion, metagenome, manure waste, full-scale biogas plant, metagenome-assembled genomes, methanogenesis

## Abstract

**Background:**

Biogas production with anaerobic digestion (AD) is one of the most promising solutions for both renewable energy production and resolving the environmental problem caused by the worldwide increase in organic waste. However, the complex structure of the microbiome in AD is poorly understood.

**Findings:**

In this study, we constructed a microbial gene catalog of AD (22,840,185 genes) based on 1,817 Gb metagenomic data derived from digestate samples of 56 full-scale biogas plants fed with diverse feedstocks. Among the gene catalog, 73.63% and 2.32% of genes were taxonomically annotated to Bacteria and Archaea, respectively, and 57.07% of genes were functionally annotated with KEGG orthologous groups. Our results confirmed the existence of core microbiome in AD and showed that the type of feedstock (cattle, chicken, and pig manure) has a great influence on carbohydrate hydrolysis and methanogenesis. In addition, 2,426 metagenome-assembled genomes were recovered from all digestate samples, and all genomes were estimated to be ≥80% complete with ≤10% contamination.

**Conclusions:**

This study deepens our understanding of the microbial composition and function in the AD process and also provides a huge number of reference genome and gene resources for analysis of anaerobic microbiota.

## Background

In the context of global climate change, in recent years the use of biogas as a renewable form of energy has increasingly drawn the world's attention. While the vast amount of organic waste caused by population expansion, urbanization expansion, and agriculture intensification continues to severely threaten the environment [1], at the same time, anaerobic digestion (AD) of biomass is considered one of the most important solutions for both producing renewable energy and resolving the problem of organic waste, such as animal manure, crop residues, and wastewater sludge [2, 3], and to date has been applied worldwide.

AD includes 4 sequential metabolic steps, namely, hydrolysis, acidogenesis, acetogenesis, and methanogenesis, and is performed by a complex consortium of bacteria and archaea [4, 5]. The first 3 steps are predominantly fulfilled synergistically by fermentative bacteria from the phyla Firmicutes, Bacteroidetes, and Proteobacteria, while the last step is carried out by methanogenic archaea from the phylum Euryarchaeota [6]. However, the structure and performance of microbial communities in AD are strongly influenced by operating factors, such as feedstock, temperature, organic loading rate, and intermediate metabolites [5, 6]. Because the microbial communities in AD are extremely complex, the microbial compositions and interactions among microbes remain largely unclear [7].

Culture-independent technologies based on high-throughput sequencing enable the deep investigation of microbial compositions and functions. High-throughput 16S ribosomal RNA gene sequencing has been frequently used to analyze the taxonomic profile of AD microbial communities [8, 9]. Metagenomic approaches alone or coupled with metatranscriptomics, metaproteomics, and metabolomics are increasingly applied to decipher the gene functions, enzyme profiles, and metabolic processes of microbial communities in AD [10, 11]. However, most of these studies have focused only on a relatively small number of full-scale anaerobic digesters or used a small amount of sequencing data [2, 3, 12, 13]. In this study, we collected different digestate samples from 56 full-scale biogas plants (BGPs), which were operated at different temperatures, fed with diverse feedstocks, and distributed widely in geographical regions, and constructed a microbial gene catalog of AD (MGCA) by in-depth metagenome sequencing.

### Data description

To construct an MGCA, 56 full-scale BGPs located all across China ranging from northeast (45.462 N, 131.604 E) to southwest (23.351 N, 103.339 E) (Supplementary Fig. S1) were investigated. All plants were operated at ambient temperature (14–31.3°C at the time of sampling) or in mesophilic (35–45°C) conditions, at pH 7.3–9.0, and with digester volume from 12 to 8,000 m^3^ (Supplementary Table S1).

Among these BGPs, 46 were in mono-digestion process, treating a single type of livestock manure (cattle, chicken, or pig manure), and the remaining 10 BGPs treated other animal manures alone or a mixture of livestock manure and other substrates, such as straw, vegetables, or sewage water (Supplementary Table S1). According to their substrate types, these investigated BGPs were divided into 4 groups: MCA (13 cattle manure BGPs), MCH (6 chicken manure BGPs), MPI (27 pig manure BGPs), and OTH (10 BGPs with other substrates) (Table 1). A total of 41 BGPs adopted the continuous stirred tank reactor (CSTR), and other BGPs adopted the upflow solids reactor (USR), anaerobic baffled reactor (ABR), or black film digester (Supplementary Table S1). Almost all of the BGPs (53 BGPs) used a single-stage process, but 3 of them used a 2-stage process (Supplementary Table S1). Overall, these BGPs cover the typical and prevailing types used with AD and constitute a representative collection.

**Table 1:** Summary of the investigated full-scale biogas plants

Group	Feedstock type	Samples^a^	BGPs	Reactor type^b^			Operating conditions^c^	
				CSTR	USR	Others	Mesophilic	Ambient
MCA	Cattle manure	14	13	11	1	1	9	4
MCH	Chicken manure	7	6	4	1	1	5	1
MPI	Pig manure	28	27	21	3	3	8	19
OTH	Other substrates	10	10	5	3	2	6	4
Total		59	56	41	8	7	28	28

a 53 Samples from 53 single-stage BGPs and 6 samples from each stage of 3 2-stage BGPs (JSP-03, SDP-01, and AHP-01).

b CSTR: continuous stirred-tank reactor; USR: upflow anaerobic solid reactor; others: anaerobic baffled reactor (ABR), black film digester, and buried digester.

c Operating conditions include mesophilic conditions and ambient temperature.

### Sample collection

Digestate samples were collected from the fermentation tank or sampling valve. Before sampling, the reactor content was stirred and the sampling valve was opened for 5 min to flush the sampling valve and tubes. Approximately 300 mL of digestate was sampled from each BGP and transferred into 6 sterile, gastight tubes (50 mL) and frozen immediately in a cooler with dry ice, and then transported to the laboratory. Frozen samples were stored at −80°C before DNA extraction. In total, 59 digestate samples were collected, including 53 samples from 53 single-stage BGPs and 6 samples from each stage of 3 different 2-stage BGPs (JSP-03, SDP-01, and AHP-01) (Supplementary Table S1).

### DNA extraction, library preparation, and sequencing

Frozen digestate samples were removed from the −80°C freezer and thawed at room temperature. Genomic DNA was extracted in triplicate using the PowerSoil DNA Isolation Kit (Cat. No. 12888–100; MoBio Laboratories Inc., Carlsbad, CA, USA) according to the manufacturer's protocol. To increase DNA yield, an extra physical cell disruption step of repeated freeze-thaw (4 cycles of alternating between 65°C and liquid nitrogen for 5 min) was used prior to the standard protocol. The integrity of DNA extracts was checked on 0.7% (w/v) agarose gel with GelRed nucleic acid gel stain (Cat. No. 41003; Biotium, Fremont, CA, USA). DNA samples showing obvious concentrated DNA bands >15 kb in size were used for further analysis (Supplementary Fig. S2). The quality and quantity of the extracted DNA were assessed using Nanodrop (Thermo Fisher Scientific, Waltham, MA, USA) and Qubit dsDNA HS assay kit (Thermo Fisher Scientific, Waltham, MA, USA). After DNA quality checks, the 3 replicates of high-quality DNA (band length >15 kb, A260/280 1.8–2.0, double-stranded DNA [dsDNA] concentration >20 ng/μL) of each sample were pooled for library construction.

Sequencing libraries were prepared for each sample using Illumina TruSeq DNA PCR-Free Library Preparation Kit (ref. 15 037 059; Illumina, San Diego, CA, USA) according to the manufacturer's instructions. In brief, a total of 1.5 µg metagenomic DNA was sheared to 350-bp fragments using Covaris S220 (Covaris, Woburn, Massachusetts, USA), and the sheared DNA fragments were purified, blunt-end repaired, and size selected. Subsequently, a single “A” nucleotide was added to the 3′ end of the blunt fragments, and then multiple indexing adapters were ligated to the A-tailed fragments by a complementary pairing single “T” nucleotide on the 3′ end. All 59 prepared sequencing libraries were first checked for quality and quantity and then paired-end sequenced (2 × 150 bp) using Illumina Hiseq X10 platform (Illumina, San Diego, CA, USA) by Cloud Health Genomics Ltd. (Shanghai, China). In total, 1,817 Gb of raw data were generated with 30.80 ± 3.77 Gb per sample (Table   2).

**Table 2:** Statistics of metagenome sequencing, assembly, and non-redundant gene catalog (MGCA)

Statistic	Mean ± SD per sample	Total^a^
Raw data (Gb)	30.80 ± 3.77	1,817
Clean data (Gb)	18.03 ± 3.29	1,064
No. of contigs^b^	243,272 ± 74,535	18,389,093
Assembled contigs length (Gb)	0.71 ± 0.19	49.38
Contig N50 value (bp)^c^	4,021 ± 758	3,267
No. of predicted genes^d^	802,716 ± 217,466	56,953,553
No. of non-redundant genes		22,840,185
Percentage of full-length genes (%)		56.45
Mean open reading frame length (bp)		790

a Total, calculated from all data, including the data derived from independent assembly of each sample and co-assembly of all unmapped reads.

b Contigs with length shorter than 1,000 bp were filtered out.

c Contig N50 value of co-assembled contigs (1,893 bp) was obviously shorter than that of independently assembled contigs of each sample (4,021 ± 758), and thus contig N50 value of all contigs (3,267 bp) was shorter than that of independent assembled contigs.

d Genes with length shorter than 102 bp were filtered out.

### Metagenome assembly and construction of the gene catalog

The Illumina raw reads were cleaned by trimming the adapter sequences and low-quality regions using 2 in-house software packages, clean_adapter and clean_lowqual [14] with default parameters, resulting in clean reads with mean error rate <0.001 and read length ≥75 bp. In addition, unpaired reads were excluded from the clean reads. Then, we obtained a total of 1,064 Gb clean data, with a mean of 18.03 ± 3.29 Gb per sample (Table 2). First, the clean reads of each sample were assembled separately by Megahit v1.1.3 (Megahit, RRID:SCR_018551) [15] under paired-end mode, and the contigs with length <1,000 bp were filtered out. Then, the assembled contigs were subjected to gene prediction using Prodigal v2.6.3 (Prodigal, RRID:SCR_011936) [16] with parameter “-p meta,” and the predicted genes with codon sequence length <102 bp were filtered out according to a previous study [17]. As a result, we obtained a mean contig number of 243,272 ± 74,535 (with contig N50 of 4,021 ± 758 bp) and gene number of 802,716 ± 217,466 for each sample (Table 2). To improve the assembly quality for less abundant species, clean reads of each sample were first mapped onto the assembled contigs of the sample with BWA-MEM (BWA, RRID:SCR_010910) [18], and then all the unmapped reads were pooled together for co-assembly. The software and parameters used for assembly and gene prediction of pooled unmapped reads were the same as above, and we obtained 4,035,874 contigs (with contig N50 of 1,893 bp) and 9,593,300 genes in total.

All the obtained genes were pooled (a total of 56,953,553 genes) and then clustered to construct an initial non-redundant gene catalog (22,844,545 genes) using CD-HIT-EST v4.6.6 (CD-HIT, RRID:SCR_007105) [19] with parameter “-c 0.95 –n 10 –G 0 –aS 0.9,” adopting the criteria of identity ≥95% and alignment coverage ≥90% of the shorter genes (Table 2). The clean reads of each sample were mapped onto this initial gene catalog by BWA-MEM, and a total of 80.66% of qualified reads (with alignment length ≥50 bp and idenity >95%) could be mapped. However, there were 4,360 genes that did not have a qualified read mapped in any sample, which may be derived from wrong assembly or extreme low abundance, and they were removed from the gene catalog. Finally, we got the final non-redundant MGCA of full-scale BGPs containing a total of 22,840,185 genes, with a mean open reading frame length of 790 bp and a full-length gene percentage of 56.45% (Table 2).

**Figure 1:**
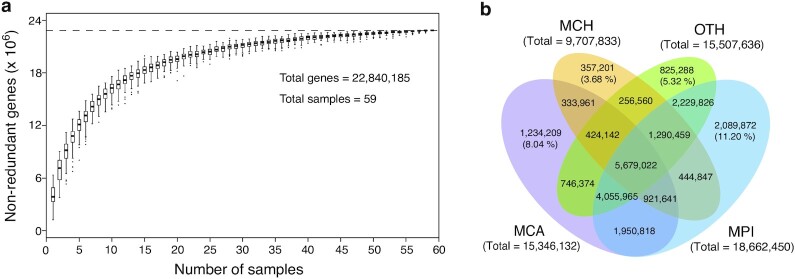
The constructed microbial gene catalog of anaerobic digestion (MGCA). **a**, Rarefaction curve of detected genes from the whole set of 59 digestate samples. The curve approaches saturation as sample number increases. The gene number of a given number of samples was calculated after 100 random samplings with replacement and plotted with a box plot. Box plots show the median ± interquartile range (IQR) and 1.5 IQR ranges (whiskers), with outliers denoted by circles. **b**, Venn diagram of shared genes among 4 groups of non-redundant genes from MCA, MCH, MPI, and OTH. Only a small proportion of genes were unique for each group. MCA: cattle manure BGPs; MCH: chicken manure BGPs; MPI: pig manure BGPs; OTH: BGPs with other substrates.

The relative gene abundances of the MGCA were calculated using the qualified reads [20, 21]. Briefly, for each sample, the total number of reads mapped to all genes (TA) equals the count of qualified reads, and the total number of reads mapped to 1 gene (TO) equals the count of qualified reads mapped to the gene. Finally, the normalized gene abundance (NGA) for each sample was calculated according the following formula: NGA = TO/(GL/1,000)/(TA/10,000,000); GL indicates the length of the gene. Rarefaction analysis was performed by counting the total number of detected genes in a given number of samples (≤59) after 100 random samplings with replacement. The rarefaction curve approached saturation with the increase of sample number (Fig. 1a), suggesting that our gene catalog covered almost all microbial genes to be found in the 56 full-scale BGPs sampled in this study. In addition, we compared the genes assigned to MCA (15,346,132 genes), MCH (9,707,833 genes), MPI (18,662,450 genes), and OTH (15,507,636 genes) and found that only a small proportion of genes (<12%) were unique in each of the 4 groups (Fig. 1b), which revealed that common microbial functions in AD were shared among different BGPs.

To assess to what extent the present MGCA could represent the microbial genes in full-scale BGPs, a more comprehensive microbial gene catalog of AD (C-MGCA) of full-scale BGPs was constructed. Besides the 59 metagenomes that were generated in this study (1,817 Gb), 39 other metagenomes (580 Gb) derived from full-scale BGPs, which were located in Germany (22 samples), United Kingdom (12 samples), Spain (4 samples), and Sweden (1 sample), were downloaded from NCBI, ENA, or MGnify database (Supplementary Table S2). All data were integrated and processed using the same pipeline for MGCA, and 25,329,366 non-redundant genes were generated for C-MGCA. On the basis of pairwise alignments of the 2 gene catalogs at gene level using BLAT (BLAT, RRID:SCR_011919) [22], we found that almost all genes in MGCA (99.99%) were shared by C-MGCA (with the criteria for shared genes that identity ≥95% and overlap ≥90% of the shorter genes), although C-MGCA only has 2,489,181 more genes than MGCA (Supplementary Fig. S3). In addition, 6 previously reported datasets derived from BGPs [23–28] were processed using the same pipeline for MGCA and compared to the 2 gene catalogs. The results showed that only 52.3 ± 9.6% of genes in these datasets were shared by MGCA, while 99.5 ± 0.7% of genes were shared by C-MGCA (Supplementary Table S3), consistent with the fact that the data of the 6 datasets were used for constructing C-MGCA. These results indicated that although MGCA contains a large proportion of genes in full-scale BGPs, the gene coverage might be further improved by collecting more diversified samples, especially for those rare genes in specific types of AD process.

### Taxonomic annotation of the gene catalog

**Figure 2:**
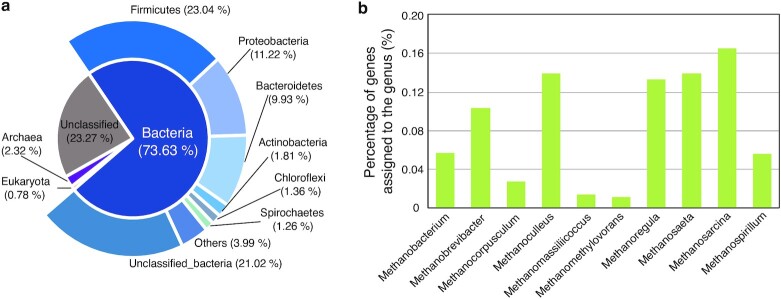
Taxonomic annotation of the microbial gene catalog of anaerobic digestion (MGCA). **a**, Taxonomic annotation of the gene catalog at the superkingdom and phylum levels. A total of 73.63% and 2.32% of genes in the gene catalog were assigned to Bacteria and Archaea, respectively. **b**, Percentage of genes assigned to the top 10 methanogenic archaea at genus level.

Taxonomic annotation of genes in MGCA was performed using CARMA3 (CARMA, RRID:SCR_004999) [29] on the basis of DIAMOND v0.8.28.90 (DIAMOND, RRID:SCR_016071) [30] alignment against the NCBI-NR database, according to a previously established method [20]. Of the 22,840,185 genes, 76.73% were taxonomically classified at the superkingdom level (Fig. 2a). Among these classified genes, 95.95% were assigned to Bacteria, and the remaining genes were assigned to Archaea (3.03%) and Eukaryota (1.02%). Firmicutes (23.04%), Proteobacteria (11.22%), and Bacteroidetes (9.93%) were the dominant phyla in the gene catalog (Fig. 2a), and Euryarchaeota (1.78%) was the predominant archaeal phylum, accounting for 76.69% of the archaeal genes. At lower taxonomic levels, only 9.62% and 0.51% of the genes were annotated to specific genera and species, respectively, highlighting the paucity of sequenced genomes of AD microbes in public databases currently. In addition, genes classified to the methanogens in BGPs include those from *Methanosarcina* (0.16%), *Methanosaeta* (0.14%), *Methanoculleus* (0.14%), *Methanoregula* (0.13%), and *Methanobrevibacter* (0.10%) (Fig. 2b). To calculate the relative abundance of different taxonomic ranks (superkingdom, phylum, class, order, family, genus, and species), the abundances of the respective genes belonging to each category according to the taxonomic assignments were added.

### Functional annotation of the gene catalog

**Figure 3:**
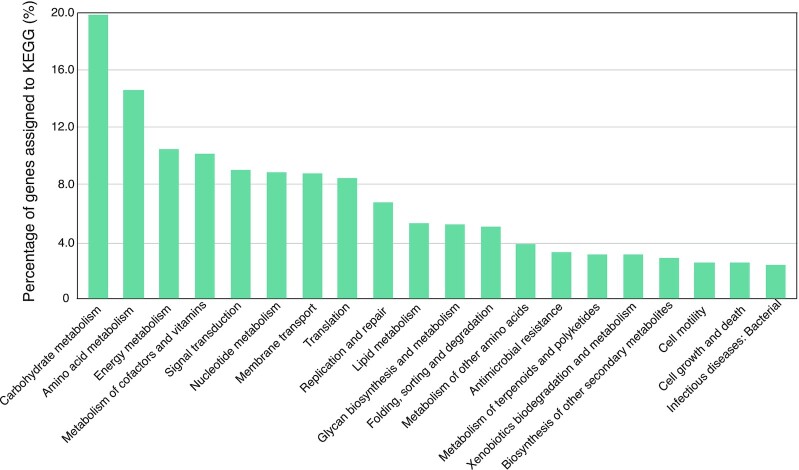
KEGG functional profile of the microbial gene catalog of anaerobic digestion (MGCA). Genes without functional annotations were excluded.

Functional annotation was performed by aligning all protein sequences in the gene catalog against the KEGG [31] database (release 79) using DIAMOND (v0.8.28.90) and taking the best hit with the coniteria of E-value < 1e−5. As a result, 57.07% of genes were annotated with KEGG orthologous groups (KOs), with a total number of 13,527 KOs that were comparable to those of the gut microbial gene catalogs of pig and chicken [20, 32]. At the KEGG pathway level, the largest categories of annotated genes were assigned to carbohydrate metabolism (19.89%), amino acid metabolism (14.61%), energy metabolism (10.52%), and metabolism of cofactors and vitamins (10.19%) (Fig. 3). In particular, 163 KOs were identified in the methane metabolism pathway, including all KOs involved in all the 3 methanogenic pathways of acetoclastic, hydrogenotrophic, and methylotrophic methanogenesis (Supplementary Fig. S4). In addition, to analyze the activities of carbohydrate hydrolysis, the genes encoding carbohydrate-active enzymes (CAZymes) were annotated by searching against the dbCAN [33] database (release 5.0) using the hmmscan program (HMMER v3.0) (HMMER, RRID:SCR_005305) [34] and taking the best hit with the criteria of E-value < 1e−18 and coverage > 0.35. A total of 1,607,960 (7.04%) genes were annotated as CAZymes. Based on the functional assignments, the relative abundance of CAZymes, KOs, and KEGG functional profiles were calculated by summing the abundance of the respective genes belonging to each category.

### Characterization of core microbial communities in full-scale biogas plants

Identifying the core microbial populations across different full-scale BGPs is important to elucidate the essential process in AD, and multiple studies have sought to define the core AD microbiome [9, 35, 36]. In the present study with the in-depth metagenomic sequencing of diverse full-scale BGPs, we found 400 genera and 6,816 KOs were shared by all the investigated samples (Supplementary Fig. S5), which accounted for ∼98.76% and 99.39% of the total relative abundance of annotated genera and KOs, respectively.

**Figure 4:**
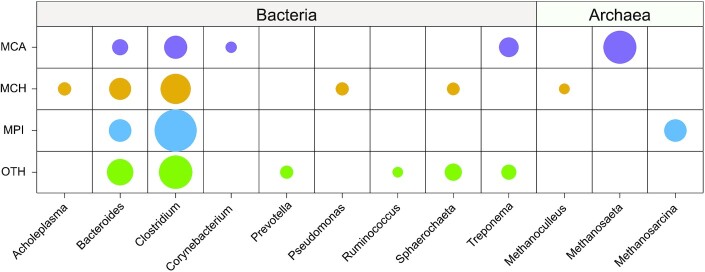
Distributions of feedstock-associated core genera among the 4 groups, MCA, MCH, MPI, and OTH. The area of each circle represents the median value of relative abundance of the corresponding genus in each group, and the non-core genera are not shown. “Core microbes” were defined as the genera most abundantly (top 30 bacterial genera and top 5 archaeal genera) detected in all studied samples. MCA: cattle manure BGPs; MCH: chicken manure BGPs; MPI: pig manure BGPs; OTH: BGPs with other substrates.

However, the majority of the common microbes were in low abundance, and only a few abundant microbes could be considered to be core members that play important roles in AD systems. Thus, we defined core microbes by including genera that were both abundant and prevalent (most abundant top 30 bacterial genera and top 5 archaeal genera that were detected in all studied samples). As a result, only *Bacteroides* and *Clostridium* (Fig. 4), within the order of Bacteroidales and Clostridiales, were identified as core microbes. The result was consistent with a previous study, which detected Bacteroidales and Clostridiales from all 29 full-scale BGPs by 16S ribosomal RNA gene amplicon sequencing [8]. However, we note that *Bacteroides* and *Clostridium* were also the abundant genera in cattle, chicken, and pig gut [20, 32, 37]. In addition, only 2 core genera were detected in all 59 samples, consistent with the phenomenon that it is hard to detect the core microbes from a high number of investigated samples [9]. To compare the difference of the 4 groups, group-specific core microbes were analyzed, which were defined by the top genera that were detected in all samples of that group. Finally, besides the genera *Bacteroides* and *Clostridium*, an additional 3 (*Corynebacterium, Treponema*, and *Methanosaeta*), 4 (*Acholeplasma, Pseudomonas, Sphaerochaeta*, and *Methanoculleus*), 1 (*Methanosarcina*), and 4 (*Prevotella, Ruminococcus, Sphaerochaeta*, and *Treponema*) genera were identified as core microbes for MCA, MCH, MPI, and OTH, respectively (Fig. 4).

### Microbial functional differentiation among BGPs with different feedstocks

**Figure 5:**
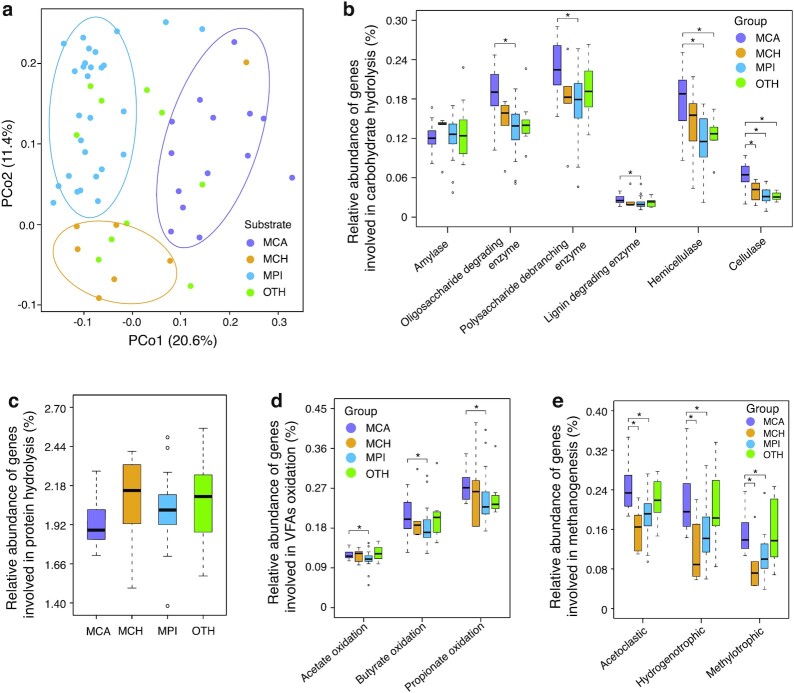
Comparisons of taxonomic and functional profiles among different biogas plants (BGPs). **a**, Principal coordinate analysis (PCoA) based on Bray-Curtis dissimilarity at the species level. The digestate samples were separated into 3 clusters (MCA, MCH, and MPI). MCA: cattle manure BGPs; MCH: chicken manure BGPs; MPI: pig manure BGPs; OTH: BGPs with other substrates. **b**, Relative abundance of genes involved in the hydrolysis of starch, oligosaccharide, polysaccharide, and lignocellulose (lignin, hemicellulose, and cellulose). **c**, Relative abundance of genes involved in protein hydrolysis. **d**, Relative abundance of genes involved in acetate, propionate, and butyrate oxidation. **e**, Relative abundance of genes involved in methanogenesis. Box plots show the median ± interquartile range (IQR) and 1.5 IQR ranges (whiskers), with outliers denoted by circles. Wilcoxon rank-sum test among different groups was performed. *P < 0.05.

Feedstock is an essential factor that drives microbial community variation in anaerobic digesters [38]. Principal coordinate analysis based on Bray-Curtis dissimilarity at the species level was performed by the R package PHYLOSEQ, revealing that digestate samples were generally separated into 3 clusters (MCA, MCH, and MPI), corresponding to the types of livestock manure (Fig. 5a). Microbial diversity (Shannon index) at the genus level also showed distinct differences among the groups, and the microbial diversity of MPI was significantly (Wilcox rank sum test *P* < 0.05) higher than those of MCA and MCH (Supplementary Fig. S6).

To find the functional differences among the 4 groups, the relative abundances of genes involved in carbohydrate hydrolysis, protein hydrolysis, volatile fatty acid (VFA) oxidation, and methanogenesis were compared. For genes involved in carbohydrate hydrolysis, we selected the CAZyme families involved in lignocellulose and starch hydrolysis and categorized them in accordance with the CAZy database and previous studies [39–43] (Supplementary Table S4). The genes involved in protein hydrolysis (with Enzyme Commission No. EC 3.4.x.x) and methanogenesis were selected on the basis of the KO annotation. The genes involved in the acetate, propionate, and butyrate oxidation pathways were selected according to the KEGG database and a previous study [44] (Supplementary Table S5).

As a result, for lignocellulose (cellulose, hemicelluloses, and lignin) degradation, the relative gene abundances were higher in MCA than in MCH (significantly higher for cellulose degradation; Wilcoxon rank-sum test, *P* < 0.05), and significantly (Wilcox rank sum test, *P* < 0.05) higher in MCA than in MPI (Fig. 5b), which is consistent with the higher content of lignocellulose in cattle manure [45]. In contrast, genes involved in starch hydrolysis have higher relative abundance in MCH and MPI (Fig. 5b). Besides, the relative abundance of genes involved in the hydrolysis of proteins was much higher in MCH (Fig. 5c), which is associated with the relatively high protein content of chicken manure [45–47]. These results are consistent with the fact that the manures of the various animals are rich in these substances because their feed is different from each other. VFAs such as acetate, propionate, and butyrate are intermediates in the anaerobic digestion process, and the accumulation of VFAs may cause acidification and result in reduced performance of the AD process. The results showed that MCH had the highest relative gene abundance involved in acetate oxidation, while MCA had significantly (Wilcoxon rank-sum test, *P* < 0.05) higher relative gene abundance involved in acetate, propionate, and butyrate oxidation than those of MPI (Fig. 5d). In addition, as one of the most important steps of biogas production, the genes involved in methanogenesis were compared, which revealed that MCH had the lowest relative gene abundance, and MCA was significantly (Wilcoxon rank-sum test, *P* < 0.05) higher than MCH and MPI (Fig. 5e). In summary, the feedstock components have great influence on the process of carbohydrate and protein hydrolysis, VFA oxidation, and methanogenesis in BGPs.

In addition, various parameters in AD also have important effects on shaping microbial communities. Several process parameters (operation temperature, pH, hydraulic retention time, and reactor volume), physicochemical characteristics of feedstock (total nitrogen, total carbon, and total solid), and intermediate metabolites (total ammonia nitrogen and VFAs) for all BGPs (Supplementary Table S1) from the groups MCA, MCH, MPI, and OTH were analyzed. Redundancy analysis at the genus level revealed that operation temperature and total ammonia nitrogen were primarily determinant parameters that influenced the microbial composition, followed by total solid, acetate, total VFAs, total nitrogren, and pH (Supplementary Fig. S7). The result was in agreement with a previous finding that total ammonia nitrogen and digester temperature were identified as the main contributing factors to cluster formation [8].

### Construction of metagenome-assembled genomes

To reconstruct the metagenome-assembled genomes (MAGs), all 59 digestate samples were included. Metagenome binning was applied to single-sample assemblies, which were performed in the “Metagenome assembly” step, and the contigs with length <1,000 bp were filtered out. BBmap v38.50 (BBmap, RRID:SCR_016965) [48] was used to map reads of each sample back to the filtered assembly with default parameters. Samtools v1.9 (Samtools, RRID:SCR_002105) [49] was used to convert SAM files to BAM format and sort the resulting BAM files. Genomes were independently recovered from each sample using MetaBAT2 v2.12.1 (MetaBAT, RRID:SCR_019134) [50], with the option –minContig 2000, and a total of 11,781 MAGs were generated from all 59 samples. The completeness (Cp) and contamination (Ct) of all MAGs were estimated using the “Lineage_wf” workflow of CheckM v1.0.7 (CheckM, RRID:SCR_016646) [51] with options lineage_wf -t 20 -x fa. After filtering for Cp ≥ 80% and Ct ≤ 10%, 3,601 MAGs were left for further de-replication.

MAG de-replication was performed using Mash v2.2 (Mash, RRID:SCR_019135) [52] on the entire genome sequences with very permissive parameters dist -d 0.05 [53], and MAGs were clustered into different groups. To determine the representative MAGs of each group, a more precise analysis was performed applying the genome-wide average nucleotide identity [54]. MAGs were considered to belong to the same species when they showed average nucleotide identity value >95% and genome coverage >50% for both strains, and the MAG with the highest CC3 value (CC3 = Cp − Ct * 3) was selected as the representative one [53]. As a result, a total of 2,426 representative MAGs were obtained, including 1,205 MAGs (49.7%) with completeness ≥90% and contamination ≤5% (Supplementary Table S6).

To estimate the degree of novelty of our study, we performed a comparison with 1,401 MAGs (Cp ≥ 70% and Ct < 10%) recovered from a previous study [53], which used 134 publicly available metagenomes derived from various biogas reactors. However, the results showed that only 108 MAGs in our study were the same species as those among the 1,401 MAGs, which could be explained by the fact that most metagenomes were derived from laboratory-scale biogas reactors and batch tests in the cited study, while all metagenomes were derived from full-scale digesters in our study. Taxonomic annotation of MAGs was performed using the GTDB-Tk v1.3.0 (GTDB-Tk, RRID:SCR_019136) [55], and 96.08% and 3.92% of MAGs were assigned to Bacteria and Archaea, respectively. In addition, Firmicutes (38.25%), Bacteroidetes (21.89%), and Proteobacteria (5.03%) were the dominant phyla in these MAGs, which was consistent with the microbial compositions at phylum level derived from the gene catalog. In summary, our study provides a huge number of MAGs for full-scale BGPs.

## Conclusions

Here, we present a microbial gene catalog of AD, by using in-depth sequencing of the digestate samples from 56 full-scale BGPs treating diverse feedstocks, and provide >22.8 million taxonomically and functionally annotated genes. Our results confirmed the existence of core microbiome in AD and showed that the type of feedstock (cattle, chicken, and pig manure) has a great influence on carbohydrate hydrolysis, VFAs oxidation, and methanogenesis. Additionally, we also provided 2,426 MAGs derived from full-scale BGPs. Compared to previously published microbial gene catalogs of different ecosystems such as soil, ocean, and animal gut and rumen [20, 32, 56–59], BGPs are man-made extremely anaerobic ecosystems where AD is performed by a complex consortium of anaerobic microbes. Hence, our gene catalog will not only serve as a useful reference database for quick analyses of AD microbiome data but also provide a huge number of microbial gene resources for the study and utilization of anaerobic microbiota.

## Data Availability

All raw sequencing data generated during the present study have been deposited at DDBJ/ENA/GenBank under project accession PRJNA533495. For details, see SRR8925713–SRR8925730, SRR8925732–SRR8925742, SRR8925747–SRR8925748, SRR8925751–SRR8925758, SRR8925797–SRR8925806, SRR8925817–SRR8925824, and SRR8925826–SRR8925827 for metagenome sequencing data of 59 digestate samples. Other supporting data, including the files of gene sequences, taxonomic and functional annotations, the abundance profile tables of the 2 gene catalogs (MGCA and C-MGCA), and metagenome-assembled genomes (MAGs) generated in this study are available in the *GigaScience* GigaDB repository [60].

## Additional Files

**Supplementary Figure S1:** Geographic distribution of 56 full-scale biogas plants (BGPs) from which the digestate samples were collected. The sampling BGPs ranged in location from the northeast (45.462 N, 131.604 E) to the southwest (23.351 N, 103.339 E) of China, including cattle manure BGPs (MCA), chicken manure BGPs (MCH), pig manure BGPs (MPI), and BGPs with other feedstocks (OTH).

**Supplementary Table S1:** Background information of the investigated 56 full-scale biogas plants (BGPs).

**Supplementary Figure S2:** Electrophoresis graph of DNA samples.

**Supplementary Table S2:** Information of the sequencing data downloaded from public database.

**Supplementary Figure S3:** Rarefaction analysis of gene catalogs MGCA and C-MGCA. The gene number of a given number of samples was calculated after 100 random samplings with replacement.

**Supplementary Table S3:** Overlap of genes between gene sets of public metagenome sequencing data and MGCA and C-MGCA.

**Supplementary Figure S4:** The KEGG methane metabolism pathway. The enzymes present in 100% of digestate samples (59 samples) are highlighted in red, the enzymes present in >90% of digestate samples are highlighted in light blue, and other enzymes annotated in the gene catalog are shown in green. The enzymes were analyzed on the basis of the KO annotation.

**Supplementary Figure S5:** The number of shared genera and KOs among biogas plants (BGPs) at different frequency thresholds.

**Supplementary Figure S6:** Shannon index of MCA, MCH, and MPI at the genus level. MCA: cattle manure biogas plants (BGPs); MCH: chicken manure BGPs; MPI: pig manure BGPs. Box plots show median ± interquartile range (IQR) and 1.5 IQR ranges (whiskers), with outliers denoted by circles. Wilcoxon rank-sum test among different groups was performed. *P < 0.05 between the 2 groups.

**Supplementary Table S4:** Categories of CAZyme families.

**Supplementary Table S5:** Genes selected for the analysis of the acetate, propionate, and butyrate oxidation pathways.

**Supplementary Figure S7:** Redundancy analysis (RDA) of microbial communities and operational parameters. Red arrows indicate the influence of process parameters (operation temperature, pH, hydraulic retention time [HRT], and reactor volume), physicochemical characteristics of feedstock (total nitrogen [TN], total carbon [TC], and total solid [TS]), and intermediate metabolites (total ammonia nitrogen [TAN] and VFAs) on microbial communities. Colored dots indicate samples of different groups of BGPs.

**Supplementary Table S6:** Statistics and taxonomic annotation of metagenome-assembled genomes (MAGs).

## Abbreviations

ABR: anaerobic baffled reactor; AD: anaerobic digestion; BGP: biogas plant; bp: base pairs; BWA: Burrows-Wheeler Aligner; CAZyme: carbohydrate-active enzyme; C-MGCA: comprehensive microbial gene catalog of AD; Cp: completeness; CSTR: continuous stirred tank reactor; Ct: contamination; DDBJ: DNA Data Bank of Japan; Gb: gigabase pairs; kb: kilobase pairs; KEGG: Kyoto Encyclopedia of Genes and Genomes; KO: KEGG orthologous group; MAG: metagenome-assembled genome; MCA: cattle manure biogas plants; MCH: chicken manure biogas plants; MGCA: microbial gene catalog of AD; MPI: pig manure biogas plants; NCBI: National Center for Biotechnology Information; OTH: biogas plants with other feedstocks; USR: upflow solids reactor; VFA: volatile fatty acid.

## Competing Interests

The authors declare that they have no competing interests.

## Funding

This project was supported by grants from Shenzhen Science and Technology Program (JCYJ20190814163805604), Agricultural Science and Technology Innovation Program (ASTIP), Chinese Academy of Agricultural Sciences (CAAS-ASTIP-2016-BIOMA), the Agricultural Science and Technology Innovation Program & The Elite Young Scientists Program of CAAS, Fundamental Research Funds for Central Non-profit Scientific Institution (No. Y2017JC01), Science and Technology Program of Sichuan Province, China (2017JY0242), the Agricultural Science and Technology Innovation Program Cooperation and Innovation Mission (CAAS-XTCX2016), the Fund of Key Laboratory of Shenzhen (ZDSYS20141118170111640), the Fundamental Research Funds for Central Non-profit Scientific Institution, China (1610012016023), and the Infrastructure and Facility Development Program of Sichuan Province (2019JDPT0012). The sponsors had no role in the design or conduct of the study; the collection, management, analysis, or interpretation of the data; the preparation, review, or approval of the manuscript; or the decision to submit the manuscript for publication.

## Authors' Contributions

S.M., Y.H., H.F., and Q.L. collected the samples, and F.J., Y.Z., L.Y., and S.L. extracted the DNA and constructed the Illumina sequencing libraries. S.M., F.J., Y.H., Y.Z., S.W., B.L., and H.W. analyzed the data. H.L. and Y.R. provided helpful suggestions. S.M., F.J., Y.H., Y.Z., and S.W. wrote the manuscript. W.F., Y.D., and L.C. conceived the study, designed the experiments, and revised the manuscript. All authors read and approved the final manuscript.

## Supplementary Material

giaa164_GIGA-D-20-00207_Original_Submission

giaa164_GIGA-D-20-00207_Revision_2

giaa164_GIGA-D-20-00207_Revision_3

giaa164_Response_to_Reviewer_Comments_Original_Submission

giaa164_Response_to_Reviewer_Comments_Revision_1

giaa164_Reviewer_1_Report_Original_Submission Christopher Hunter, Ph.D. -- 7/17/2020 Reviewed

giaa164_Reviewer_1_Report_Revision_1 Christopher Hunter, Ph.D. -- 11/13/2020 Reviewed

giaa164_Reviewer_2_Report_Original_Submission James P. J. Chong -- 8/5/2020 Reviewed

giaa164_Reviewer_3_Report_Original_Submission KornÃ©l L. KovÃ¡cs -- 8/17/2020 Reviewed

giaa164_Supplemental_Files

## References

[1] Tyagi VK, Lo SL. Sludge: A waste or renewable source for energy and resources recovery?. Renew Sust Energ Rev. 2013;25:708–28.

[2] Stolze Y, Bremges A, Rumming M, et al. Identification and genome reconstruction of abundant distinct taxa in microbiomes from one thermophilic and three mesophilic production-scale biogas plants. Biotechnol Biofuels. 2016;9:156. 27462367 10.1186/s13068-016-0565-3 PMC4960831

[3] Luo G, Fotidis IA, Angelidaki I. Comparative analysis of taxonomic, functional, and metabolic patterns of microbiomes from 14 full-scale biogas reactors by metagenomic sequencing and radioisotopic analysis. Biotechnol Biofuels. 2016;9:51. 26941838 10.1186/s13068-016-0465-6 PMC4776419

[4] Angenent LT, Karim K, Al-Dahhan MH, et al. Production of bioenergy and biochemicals from industrial and agricultural wastewater. Trends Biotechnol. 2004;22:477–85. 15331229 10.1016/j.tibtech.2004.07.001

[5] Hassa J, Maus I, Off S, et al. Metagenome, metatranscriptome, and metaproteome approaches unraveled compositions and functional relationships of microbial communities residing in biogas plants. Appl Microbiol Biotechnol. 2018;102:5045–63. 29713790 10.1007/s00253-018-8976-7 PMC5959977

[6] Schnürer A . Biogas production: Microbiology and technology. Adv Biochem Eng Biotechnol. 2016;156:195–234. 27432246 10.1007/10_2016_5

[7] Narihiro T, Nobu MK, Kim NK, et al. The nexus of syntrophy-associated microbiota in anaerobic digestion revealed by long-term enrichment and community survey. Environ Microbiol. 2015;17:1707–20. 25186254 10.1111/1462-2920.12616

[8] De Vrieze J, Saunders AM, He Y, et al. Ammonia and temperature determine potential clustering in the anaerobic digestion microbiome. Water Res. 2015;75:312–23. 25819618 10.1016/j.watres.2015.02.025

[9] Mei R, Nobu MK, Narihiro T, et al. Operation-driven heterogeneity and overlooked feed-associated populations in global anaerobic digester microbiome. Water Res. 2017;124:77–84. 28750287 10.1016/j.watres.2017.07.050

[10] Jia Y, Ng SK, Lu H, et al. Genome-centric metatranscriptomes and ecological roles of the active microbial populations during cellulosic biomass anaerobic digestion. Biotechnol Biofuels. 2018;11:117. 29713376 10.1186/s13068-018-1121-0 PMC5911951

[11] Treu L, Kougias PG, Campanaro S, et al. Deeper insight into the structure of the anaerobic digestion microbial community; the biogas microbiome database is expanded with 157 new genomes. Bioresour Technol. 2016;216:260–6. 27243603 10.1016/j.biortech.2016.05.081

[12] Campanaro S, Treu L, Kougias PG, et al. Metagenomic binning reveals the functional roles of core abundant microorganisms in twelve full-scale biogas plants. Water Res. 2018;140:123–34. 29704757 10.1016/j.watres.2018.04.043

[13] Campanaro S, Treu L, Kougias PG, et al. Metagenomic analysis and functional characterization of the biogas microbiome using high throughput shotgun sequencing and a novel binning strategy. Biotechnol Biofuels. 2016;9:26. 26839589 10.1186/s13068-016-0441-1 PMC4736482

[14] Clean_adapter and clean_lowqul GitHub repository. https://github.com/fanagislab/DBG_assembly/tree/master/clean_illumina. Accessed on 2019.

[15] Li DH, Luo RB, Liu CM, et al. MEGAHIT v1.0: A fast and scalable metagenome assembler driven by advanced methodologies and community practices. Methods. 2016;102:3–11. 27012178 10.1016/j.ymeth.2016.02.020

[16] Hyatt D, LoCascio PF, Hauser LJ, et al. Gene and translation initiation site prediction in metagenomic sequences. Bioinformatics. 2012;28:2223–30. 22796954 10.1093/bioinformatics/bts429

[17] Qin J, Li R, Raes J, et al. A human gut microbial gene catalogue established by metagenomic sequencing. Nature. 2010;464:59–65. 20203603 10.1038/nature08821 PMC3779803

[18] Li H, Durbin R. Fast and accurate short read alignment with Burrows-Wheeler transform. Bioinformatics. 2009;25:1754–60. 19451168 10.1093/bioinformatics/btp324 PMC2705234

[19] Fu LM, Niu BF, Zhu ZW, et al. CD-HIT: Accelerated for clustering the next-generation sequencing data. Bioinformatics. 2012;28:3150–52. 23060610 10.1093/bioinformatics/bts565 PMC3516142

[20] Huang P, Zhang Y, Xiao KP, et al. The chicken gut metagenome and the modulatory effects of plant-derived benzylisoquinoline alkaloids. Microbiome. 2018;6:211. 30482240 10.1186/s40168-018-0590-5 PMC6260706

[21] Qin JJ, Li YR, Cai ZM, et al. A metagenome-wide association study of gut microbiota in type 2 diabetes. Nature. 2012;490:55–60. 23023125 10.1038/nature11450

[22] Kent WJ . BLAT–the BLAST-like alignment tool. Genome Res. 2002;12:656–64. 11932250 10.1101/gr.229202 PMC187518

[23] Maus I, Koeck DE, Cibis KG, et al. Unraveling the microbiome of a thermophilic biogas plant by metagenome and metatranscriptome analysis complemented by characterization of bacterial and archaeal isolates. Biotechnol Biofuels. 2016;9:171. 27525040 10.1186/s13068-016-0581-3 PMC4982221

[24] Ortseifen V, Stolze Y, Maus I, et al. An integrated metagenome and -proteome analysis of the microbial community residing in a biogas production plant. J Biotechnol. 2016;231:268–79. 27312700 10.1016/j.jbiotec.2016.06.014

[25] Gullert S, Fischer MA, Turaev D, et al. Deep metagenome and metatranscriptome analyses of microbial communities affiliated with an industrial biogas fermenter, a cow rumen, and elephant feces reveal major differences in carbohydrate hydrolysis strategies. Biotechnol Biofuels. 2016;9:121. 27279900 10.1186/s13068-016-0534-x PMC4897800

[26] Bremges A, Maus I, Belmann P, et al. Deeply sequenced metagenome and metatranscriptome of a biogas-producing microbial community from an agricultural production-scale biogas plant. Gigascience. 2015;4:33. 26229594 10.1186/s13742-015-0073-6 PMC4520284

[27] Sun L, Muller B, Westerholm M, et al. Syntrophic acetate oxidation in industrial CSTR biogas digesters. J Biotechnol. 2014;171:39–44. 24333792 10.1016/j.jbiotec.2013.11.016

[28] Ruiz-Sanchez J, Campanaro S, Guivernau M, et al. Effect of ammonia on the active microbiome and metagenome from stable full-scale digesters. Bioresour Technol. 2018;250:513–22. 29197774 10.1016/j.biortech.2017.11.068

[29] Gerlach W, Stoye J. Taxonomic classification of metagenomic shotgun sequences with CARMA3. Nucleic Acids Res. 2011;39:e91. 21586583 10.1093/nar/gkr225 PMC3152360

[30] Buchfink B, Xie C, Huson DH. Fast and sensitive protein alignment using DIAMOND. Nat Methods. 2015;12:59–60. 25402007 10.1038/nmeth.3176

[31] Kanehisa M, Goto S, Kawashima S, et al. The KEGG resource for deciphering the genome. Nucleic Acids Res. 2004;32:D277–D80. 14681412 10.1093/nar/gkh063 PMC308797

[32] Xiao L, Estelle J, Kiilerich P, et al. A reference gene catalogue of the pig gut microbiome. Nat Microbiol. 2016;1:16161. 27643971 10.1038/nmicrobiol.2016.161

[33] Yin YB, Mao XZ, Yang JC, et al. dbCAN: A web resource for automated carbohydrate-active enzyme annotation. Nucleic Acids Res. 2012;40:W445–W51. 22645317 10.1093/nar/gks479 PMC3394287

[34] Eddy SR . Accelerated profile HMM searches. PLoS Comput Biol. 2011;7:e1002195. 22039361 10.1371/journal.pcbi.1002195 PMC3197634

[35] Mei R, Narihiro T, Nobu MK, et al. Evaluating digestion efficiency in full-scale anaerobic digesters by identifying active microbial populations through the lens of microbial activity. Sci Rep. 2016;6:34090. 27666090 10.1038/srep34090 PMC5036182

[36] Calusinska M, Goux X, Fossepre M, et al. A year of monitoring 20 mesophilic full-scale bioreactors reveals the existence of stable but different core microbiomes in bio-waste and wastewater anaerobic digestion systems. Biotechnol Biofuels. 2018;11:196. 30038663 10.1186/s13068-018-1195-8 PMC6052691

[37] Wirth R, Kadar G, Kakuk B, et al. The planktonic core microbiome and core functions in the cattle rumen by next generation sequencing. Front Microbiol. 2018;9:2285. 30319585 10.3389/fmicb.2018.02285 PMC6165872

[38] Zhang W, Werner JJ, Agler MT, et al. Substrate type drives variation in reactor microbiomes of anaerobic digesters. Bioresour Technol. 2014;151:397–401. 24183494 10.1016/j.biortech.2013.10.004

[39] Artzi L, Bayer EA, Moraïs S. Cellulosomes: Bacterial nanomachines for dismantling plant polysaccharides. Nat Rev Microbiol. 2016;15:83–95. 27941816 10.1038/nrmicro.2016.164

[40] Gharechahi J, Salekdeh GH. A metagenomic analysis of the camel rumen's microbiome identifies the major microbes responsible for lignocellulose degradation and fermentation. Biotechnol Biofuels. 2018;11:216. 30083229 10.1186/s13068-018-1214-9 PMC6071333

[41] Kougias PG, Campanaro S, Treu L, et al. Spatial distribution and diverse metabolic functions of lignocellulose-degrading uncultured bacteria as revealed by genome-centric metagenomics. Appl Environ Microbiol. 2018;84:e01244–18. 30006398 10.1128/AEM.01244-18 PMC6121989

[42] Liu N, Li H, Chevrette MG, et al. Functional metagenomics reveals abundant polysaccharide-degrading gene clusters and cellobiose utilization pathways within gut microbiota of a wood-feeding higher termite. ISME J. 2019;13:104–17. 30116044 10.1038/s41396-018-0255-1 PMC6298952

[43] Zhu N, Yang J, Ji L, et al. Metagenomic and metaproteomic analyses of a corn stover-adapted microbial consortium EMSD5 reveal its taxonomic and enzymatic basis for degrading lignocellulose. Biotechnol Biofuels. 2016;9:243. 27833656 10.1186/s13068-016-0658-z PMC5103373

[44] Mosbaek F, Kjeldal H, Mulat DG, et al. Identification of syntrophic acetate-oxidizing bacteria in anaerobic digesters by combined protein-based stable isotope probing and metagenomics. ISME J. 2016;10:2405–18. 27128991 10.1038/ismej.2016.39 PMC5030692

[45] Wang M, Li W, Li P, et al. An alternative parameter to characterize biogas materials: Available carbon-nitrogen ratio. Waste Manag. 2017;62:76–83. 28259537 10.1016/j.wasman.2017.02.025

[46] Sheu SY, Liu LP, Chen WM. *Novosphingobium bradum* sp. nov., isolated from a spring. Int J Syst Evol Microbiol. 2016;66:5083–90. 27599476 10.1099/ijsem.0.001475

[47] Zakharyuk A, Kozyreva L, Ariskina E, et al. *Alkaliphilus namsaraevii* sp. nov., an alkaliphilic iron- and sulfur-reducing bacterium isolated from a steppe soda lake. Int J Syst Evol Microbiol. 2017;67:1990–95. 28632119 10.1099/ijsem.0.001904

[48] BBMap. sourceforge. https://sourceforge.net/projects/bbmap/. Accessed on 2019.

[49] Li H, Handsaker B, Wysoker A, et al. The Sequence Alignment/Map format and SAMtools. Bioinformatics. 2009;25:2078–9. 19505943 10.1093/bioinformatics/btp352 PMC2723002

[50] Kang DD, Li F, Kirton E, et al. MetaBAT 2: An adaptive binning algorithm for robust and efficient genome reconstruction from metagenome assemblies. Peer J. 2019;7:e7359. 31388474 10.7717/peerj.7359 PMC6662567

[51] Parks DH, Imelfort M, Skennerton CT, et al. CheckM: Assessing the quality of microbial genomes recovered from isolates, single cells, and metagenomes. Genome Res. 2015;25:1043–55. 25977477 10.1101/gr.186072.114 PMC4484387

[52] Ondov BD, Treangen TJ, Melsted P, et al. Mash: Fast genome and metagenome distance estimation using MinHash. Genome Biol. 2016;17:132. 27323842 10.1186/s13059-016-0997-x PMC4915045

[53] Campanaro S, Treu L, Rodriguez RL, et al. New insights from the biogas microbiome by comprehensive genome-resolved metagenomics of nearly 1600 species originating from multiple anaerobic digesters. Biotechnol Biofuels. 2020;13:25. 32123542 10.1186/s13068-020-01679-y PMC7038595

[54] Varghese NJ, Mukherjee S, Ivanova N, et al. Microbial species delineation using whole genome sequences. Nucleic Acids Res. 2015;43:6761–71. 26150420 10.1093/nar/gkv657 PMC4538840

[55] Chaumeil PA, Mussig AJ, Hugenholtz P, et al. GTDB-Tk: A toolkit to classify genomes with the Genome Taxonomy Database. Bioinformatics. 2020;36:1925–7. 10.1093/bioinformatics/btz848 PMC7703759 31730192

[56] Li J, Zhong H, Ramayo-Caldas Y, et al. A catalog of microbial genes from the bovine rumen unveils a specialized and diverse biomass-degrading environment. Gigascience. 2020;9, doi:10.1093/gigascience/giaa057. PMC7260996 32473013

[57] Bahram M, Hildebrand F, Forslund SK, et al. Structure and function of the global topsoil microbiome. Nature. 2018;560:233–37. 30069051 10.1038/s41586-018-0386-6

[58] Sunagawa S, Coelho LP, Chaffron S, et al. Ocean plankton. Structure and function of the global ocean microbiome. Science. 2015;348:1261359. 25999513 10.1126/science.1261359

[59] Li J, Jia H, Cai X, et al. An integrated catalog of reference genes in the human gut microbiome. Nat Biotechnol. 2014;32:834–41. 24997786 10.1038/nbt.2942

[60] Ma SC, Jiang F, Huang Y, et al. Supporting data for “A microbial gene catalog of anaerobic digestion from full-scale biogas plants.”. GigaScience Database. 2020, 10.5524/100842. PMC7842101 33506264

