# Supplementary material for: A microbial gene catalog of anaerobic digestion from full-scale biogas plants
Source: Gigascience. 2021 Jan 27;10(1):giaa164. doi: 10.1093/gigascience/giaa164 (PMC7842101; doi:10.1093/gigascience/giaa164)
Supplement: giaa164_GIGA-D-20-00207_Original_Submission [file giaa164_giga-d-20-00207_original_submission.pdf]

GigaScience

A microbial gene catalog of anaerobic digestion from full-scale biogas plants

--Manuscript Draft--

**Manuscript Number:**	GIGA-D-20-00207
**Full Title:**	A microbial gene catalog of anaerobic digestion from full-scale biogas plants
**Article Type:**	Data Note

Funding Information:

Funding	Recipient
Infrastructure and Facility Development Program of Sichuan Province (2019JDPT0012)	Dr Yu Deng
Shenzhen science and technology program (JCYJ20190814163805604)	Dr Wei Fan
Agricultural Science and Technology Innovation Program (ASTIP), Chinese Academy of Agricultural Sciences (CAAS-ASTIP-2016-BIOMA)	Dr Yu Deng
Agricultural Science and Technology Innovation Program && The Elite Young Scientists Program of CAAS (None)	Dr Wei Fan
Fundamental Research Funds for Central Non-profit Scientific Institution (Y2017JC01)	Dr Wei Fan
Science and Technology Program of Sichuan Province, China (2017JY0242)	Dr Shichun Ma
Agricultural Science and Technology Innovation Program Cooperation and Innovation Mission (CAAS-XTCX2016)	Dr Wei Fan
Fund of Key Laboratory of Shenzhen (ZDSYS20141118170111640)	Not applicable
Fundamental Research Funds for Central Non-profit Scientific Institution, China (1610012016023)	Dr Shichun Ma

Abstract:

Background

Biogas production with anaerobic digestion (AD) is one of the most promising solutions for both renewable energy production and resolving the environmental problem caused by the increase in organic wastes worldwide. However, the complex structure of the microbiome in AD is less understood.

Findings

In this study, we constructed the first comprehensive microbial gene catalog of AD (22,840,185 genes), based on 1,817 gigabase (Gb) metagenomic data, derived from digestate samples of 56 full-scale biogas plants fed with diverse feedstocks. Among the gene catalog, 73.63% and 2.32% of genes were taxonomically annotated to Bacteria and Archaea, respectively and 57.07% of genes were functionally annotated with KEGG orthologous groups. In addition, our results confirmed the existence of core microbiome in AD, and showed that the type of feedstock (cattle, chicken and pig manure) has a great influence on carbohydrate and protein hydrolysis and methanogenesis.

Conclusions

This study deepens our understanding of microbial compositions and functions in AD process, and also provides a huge number of reference gene resources for analysis of anaerobic microbiota.

Corresponding Author: Wei Fan
Chinese Academy of Agricultural Sciences

Powered by Editorial Manager® and ProduXion Manager® from Aries Systems Corporation

	CHINA
**Corresponding Author Secondary Information:**	
**Corresponding Author's Institution:**	Chinese Academy of Agricultural Sciences
**Corresponding Author's Secondary Institution:**	
**First Author:**	Shichun Ma
**First Author Secondary Information:**	
**Order of Authors:**	Shichun Ma
	Fan Jiang
	Yan Huang
	Yan Zhang
	Sen Wang
	Hui Fan
	Bo Liu
	Qiang Li
	Lijuan Yin
	Hengchao Wang
	Hangwei Liu
	Yuwei Ren
	Shuqu Li
	Lei Cheng
	Wei Fan
	Yu Deng
**Order of Authors Secondary Information:**	
**Additional Information:**	
**Question**	**Response**
Are you submitting this manuscript to a special series or article collection?	No
**Experimental design and statistics**<br><br>Full details of the experimental design and statistical methods used should be given in the Methods section, as detailed in our Minimum Standards Reporting Checklist. Information essential to interpreting the data presented should be made available in the figure legends.<br><br>Have you included all the information requested in your manuscript?	Yes

**Resources**<br><br>A description of all resources used, including antibodies, cell lines, animals and software tools, with enough information to allow them to be uniquely identified, should be included in the Methods section. Authors are strongly encouraged to cite Research Resource Identifiers (RRIDs) for antibodies, model organisms and tools, where possible.<br><br>Have you included the information requested as detailed in our Minimum Standards Reporting Checklist?	Yes
**Availability of data and materials**<br><br>All datasets and code on which the conclusions of the paper rely must be either included in your submission or deposited in publicly available repositories (where available and ethically appropriate), referencing such data using a unique identifier in the references and in the "Availability of Data and Materials" section of your manuscript.<br><br>Have you have met the above requirement as detailed in our Minimum Standards Reporting Checklist?	Yes

Powered by Editorial Manager® and ProduXion Manager® from Aries Systems Corporation

Manuscript

Click here to access/download;Manuscript;Manuscript-Gigascience-data-note.docx

A microbial gene catalog of anaerobic digestion from full-scale biogas plants

Shichun Ma[1,3]*, Fan Jiang[2]*, Yan Huang[1,3]*, Yan Zhang[2], Sen Wang[2], Hui Fan[1,3], Bo liu[2], Qiang Li[1,3], Lijuan Yin[2], Hengchao Wang[2], Hangwei Liu[2], Yuwei Ren[2], Shuqu Li[2], Lei Cheng[1,3], Wei Fan[2]†, and Yu Deng[1,3]†

Affiliations:

[1]Biogas Institute of Ministry of Agricultural and Rural Affairs, Chengdu, Sichuan, 610041, China.

[2]Guangdong Laboratory for Lingnan Modern Agriculture (Shenzhen Branch), Genome Analysis Laboratory of the Ministry of Agriculture and Rural Affairs, Agricultural Genomics Institute at Shenzhen, Chinese Academy of Agricultural Sciences, Shenzhen, Guangdong, 518120, China.

[3]Laboratory of Development and Application of Rural Renewable Energy, Ministry of Agricultural and Rural Affairs, Chengdu, Sichuan, 610041, China.

Shichun Ma: mashichun@caas.cn; Fan Jiang: greatjf@163.com; Yan Huang: huangyan01@caas.cn; Yan Zhang: milrazhang@163.com; Sen Wang: wangsen1993@163.com; Hui Fan: fanhui01@caas.cn; Bo Liu: lb_bobo@aliyun.com; Qiang Li: liqiang03@caas.cn; Lijuan Yin: yinlijuan1005@163.com; Hengchao Wang: wanghengchao000@qq.com; Hangwei Liu: liuhangwei2014@163.com; Yuwei Ren: xiaoshudaxia@126.com; Shuqu Li: lishuqu1234@163.com; Lei Cheng: chenglei@caas.cn.

*These authors contributed equally to this work.

†Corresponding Authors: Wei Fan and Yu Deng

E-mail: fanwei@caas.cn and

Abstract

Background: Biogas production with anaerobic digestion (AD) is one of the most promising solutions for both renewable energy production and resolving the environmental problem caused by the increase in organic wastes worldwide. However, the complex structure of the microbiome in AD is less understood.

Findings: In this study, we constructed the first comprehensive microbial gene catalog of AD (22,840,185 genes), based on 1,817 gigabase (Gb) metagenomic data, derived from digestate samples of 56 full-scale biogas plants fed with diverse feedstocks. Among the gene catalog, 73.63% and 2.32% of genes were taxonomically annotated to Bacteria and Archaea, respectively and 57.07% of genes were functionally annotated with KEGG orthologous groups. In addition, our results confirmed the existence of core microbiome in AD, and showed that the type of feedstock (cattle, chicken and pig manure) has a great influence on carbohydrate and protein hydrolysis and methanogenesis.

Conclusions: This study deepens our understanding of microbial compositions and

functions in AD process, and also provides a huge number of reference gene resources for analysis of anaerobic microbiota.

Keywords: Anaerobic digestion, metagenome, manure waste, full-scale biogas plant, methanogenesis

Background

In recent years, renewable energy resources have become increasingly recognized as a global issue because of the shortage of fossil fuels. Meanwhile, the vast amount of organic waste caused by population expansion, urbanization expansion and agriculture intensification severely threatens the environment [1]. At the same time, production of biogas by anaerobic digestion (AD) of biomass is considered as one of the most important solutions for both producing renewable energy and resolving the problem of organic wastes, such as animal manure, crop residues and wastewater sludge [2, 3], and now has been applied worldwide over the last two decades.

Anaerobic digestion includes four sequential metabolic steps, namely hydrolysis, acidogenesis, acetogenesis, and methanogenesis, and performed by a complex

consortium of bacteria and archaea [4, 5]. The first three steps are mainly synergistically fulfilled by fermentative bacteria from the phyla *Firmicutes*, *Bacteroidetes*, and *Proteobacteria*, while the last step is carried out by methanogenic archaea from the phylum *Euryarchaeota* [6]. However, the structure and performance of microbial communities in AD were strongly influenced by operating factors, such as feedstock, temperature, organic loading rate, and intermediate metabolites[5, 6]. Since the microbial communities in AD are extremely complex, the microbial compositions and interactions among microbes remain largely unclear [7].

Culture-independent technologies based on high-throughput sequencing enable the deep investigation of microbial compositions and functions. High-throughput 16S rRNA gene sequencing has been frequently used to analyze the taxonomic profile of AD microbial communities [8, 9]. Metagenomic approaches alone or coupled with metatranscriptomics, metaproteomics, and metabolomics are increasingly applied to decipher the gene functions, enzyme profiles, and metabolic processes of microbial communities in AD [10, 11]. However, most of these studies have focused only on laboratory-scale anaerobic digesters or a relatively small number of full-scale anaerobic

digesters [2, 3, 12, 13]. In this study, we collected diverse digestate samples from 56 full-scale biogas plants (BGPs) all across China, located ranging from Northeast to Southwest, and constructed a first comprehensive microbial gene catalog of AD by in-depth metagenome sequencing.

Data description

According to national statistics, by the end of 2015, there was a total number of 110,975 biogas plants been established in China, including 6,737 large-scale and 34 extra large-scale biogas plants. The most majority of these plants（99.6%）use livestock manure as main components of feedstock. To construct a comprehensive microbial gene catalog of AD, 56 full-scale BGPs located all across China ranging from Northeast (45°27' N, 131°36' E) to Southwest (23°21' N, 131°36' E) (**Additional file 1: Fig S1**) were investigated. All plants were operated at mesophilic conditions (35-45°C) or ambient temperature, at pH 7.3-9.0, and with digester volume from 12 to 8000 m^3 (**Additional file 2: Table S1**).

Among these BGPs, 46 were in mono-digestion process, treating one of livestock

manure (cattle, chicken or pig manure) alone, and the remaining 10 BGPs treat other animal manures alone or mixture of livestock manure and other substrates, such as straw, vegetable or sewage water (**Additional file 2: Table S1**). According to their substrate types, these investigated BGPs were divided into four groups: MCA (13 cattle manure BGPs), MCH (6 chicken manure BGPs), MPI (27 pig manure BGPs), and OTH (10 BGPs with other substrates) (**Table 1**). There was a total number of 41 BGPs that adopt continuous stirred tank reactor (CSTR), and other BGPs adopt upflow solids reactor (USR), anaerobic baffled reactor (ABR), or black film digester (**Additional file 2: Table S1**). The most majority of these BGPs (53 BGPs) were in single-stage process and there were also 3 BGPs applied two-stage processes (**Additional file 2: Table S1**). Overall, these BGPs covers the typical and prevailing BGP types in China and constitute a well representative collection.

Sample collection

Digestate samples were collected from fermentation tank or sampling valve. Before sampling, the reactor content was stirred and the sampling valve was opened for 5 min

to flush the sampling valve and tubes. About 300 ml of digestate was sampled from each BGPs and transferred into 6 sterile, gastight tubes (50 ml) and frozen immediately in a cooler with dry ice, and then transported to the laboratory. Frozen samples were stored at -80℃ before DNA extraction. In total, 59 digestate samples were collected, with 53 samples from 53 single-stage BGPs and 6 samples from each stage of 3 two-stage BGPs (JSP-03, SDP-01, and AHP-01) (**Additional file 2: Table S1**).

Table 1. Summary of the investigated full-scale biogas plants

Group	Feedstock type	Sample number[#]	BGP number	Reactor types [†]			Operate conditions[‡]	
				CSTR	USR	Others	Mesophilic	Ambient
MCA	Cattle manure	14	13	11	1	1	9	4
MCH	Chicken manure	7	6	4	1	1	5	1
MPI	Pig manure	28	27	21	3	3	8	19
OTH	Other substrates	10	10	5	3	2	6	4
Total		59	56	41	8	7	28	28

[#]: 53 samples from 53 single-stage BGPs and 6 samples from each stage of 3 two-stage BGPs (JSP-03, SDP-01, and AHP-01); [†]: Reactor types including CSTR, continuous stirred-tank reactor; USR, upflow anaerobic solid reactor; Others, including anaerobic baffled reactor (ABR), black film digester, and buried digester. [‡]: Operate conditions including mesophilic conditions and ambient temperature.

DNA extraction, library preparation and sequencing

Frozen digestate samples were taken out from -80℃ refrigerator and thawed at room temperature. Genomic DNA was extracted in triplicate using the PowerSoil DNA Isolation Kit (cat. no. 12888-100; MoBio Laboratories Inc., USA) according to the manufacturer's protocol with a minor modification. Briefly, a physical lysis step of four freeze-thaw cycles (alternating between 65℃ and liquid nitrogen for 5 min) was added prior to the standard protocol. The integrity of DNA extracts was checked on 0.7% (w/v) agarose gel with GelRed nucleic acid gel stain (cat. no. 41003; Biotium, USA). The quality and quantity of the extracted DNA were assessed using Nanodrop (Thermo Fisher Scientific, USA) and Qubit dsDNA HS assay kit (Thermo Fisher Scientific, USA). After DNA quality checks, the triplicate of DNA extracts of each sample was pooled for library construction.

Sequencing libraries were prepared for each sample using Illumina TruSeq DNA PCR-Free Library Preparation Kit (ref. 15037059; Illumina, USA) according to the manufacturer's instructions. In brief, a total of 1.5 μg metagenomic DNA was sheared to 350 bp fragments using Covaris S220 (Covaris, USA), and the sheared DNA

fragments were purified, blunt-end-repaired and size selected. Subsequently, a single 'A' nucleotide was added to the 3' end of the blunt fragments, and then multiple indexing adapters were ligated to the A-tailed fragments by a complementary pairing single 'T' nucleotide on the 3' end. All 59 prepared sequencing libraries were firstly checked for quality and quantity and then paired-end sequenced (2 x 150 bp) using Illumina Hiseq X10 platform (Illumina, USA) by Cloud Health Genomics Ltd (Shanghai, China). In total, 1,817 Gb of raw data were generated with 30.80 ± 3.77 Gb per sample (**Table 2**).

Table 2. Statistics of metagenome sequencing, assembly and non-redundant gene catalog

	Average value of each sample ± SD	Total [#]
Raw data (Gb)	30.80 ± 3.77	1,817
Clean data (Gb)	18.03 ± 3.29	1,064
Number of contigs [†]	243,272 ± 74,535	18,389,093
Assembled contigs length (Gb)	0.71 ± 0.19	49.38
Contig N50 value (bp)[*]	4,021 ± 758	3,267
Number of predicted genes[‡]	802,716 ± 217,466	56,953,553
Number of non-redundant genes	-	22,840,185
Percentage of full-length genes	-	56.45%
Average open reading frame length (bp)	-	790

[#]: Total, calculated from all data, including the data derived from independent assembly of each

sample and co-assembly of all unmapped reads; †: contigs with length shorter than 1000 bp were filtered out; *: contig N50 value of co-assembled contigs (1,893 bp) were obviously shorter than that of independently assembled contigs of each sample (4,021 ± 758), and thus contig N50 value of all contigs (3,267 bp) were shorter than that of independent assembled contigs; ‡: genes with length shorter than 102 bp were filtered out.

Metagenome assembly and construction of the gene catalog

The Illumina raw reads were cleaned by trimming the adapter sequences and low-quality regions using two in-house software clean_adapter and clean_lowqual [14] with default parameters, resulting in the clean reads with average error rate < 0.001 and read length ≥ 75 bp. In addition, unpaired reads were excluded from the clean reads. Then, we obtained a total of 1,064 Gb clean data, with an average of 18.03 ± 3.29 Gb per sample (**Table 2**). Firstly, the clean reads of each sample were assembled separately by Megahit (v1.1.3) [15] under paired-end mode, and the contigs with length < 1000 bp were filtered out. Then, the assembled contigs were subjected to gene prediction using Prodigal v2.6.3 (Prodigal, RRID:SCR_011936) [16] with parameter "-p meta", and the predicted genes with codon sequence length < 102 bp were filtered out according to a previous study [17]. As a result, we obtained an average contig number of 243,272 ±

74,535 (with contig N50 of 4,021 ± 758 bp) and gene number of 802,716 ± 217,466 for each sample (**Table 2**). To improve the assembly quality for less abundant species, clean reads of each sample were firstly mapped onto the assembled contigs of the sample with BWA-MEM (BWA, RRID:SCR_010910) [18], and then all the unmapped reads were pooled together for co-assembly. The software and parameters used for assembly and gene prediction of pooled unmapped reads were the same as above, and we obtained 4,035,874 contigs (with contig N50 of 1,893 bp) and 9,593,300 genes in total.

All the obtained genes were pooled (a total of 56,953,553 genes) and then clustered to construct an initial non-redundant gene catalog (22,844,545 genes) using CD-HIT-EST (v4.6.6) [19] with parameter "-c 0.95 –n 10 –G 0 –aS 0.9", adopts the criteria of identity ≥ 95% and alignment coverage ≥ 90% of the shorter genes (**Table 2**). The clean reads of each sample were mapped onto this gene catalog by BWA-MEM, and a total of 80.66% of reads could be mapped with alignment length ≥ 50 bp and identity > 95%. With the mapped reads, we calculated the relative gene abundance as previously described [20, 21]. In addition, 4,360 genes were removed from the gene catalog as they have no read mapped. At last, we got the final non-redundant gene

catalog of full-scale BGPs containing a total of 22,840,185 genes, with an average open reading frame length of 790 bp and a full-length gene percentage of 56.45% (**Table 2**).

To assess to what extent our gene catalog could represent the microbial genes in full-scale BGPs, rarefaction analysis was performed by counting the total number of detected genes in a given number of samples (≤ 59) after 100 random samplings with replacement. The rarefaction curve approached saturation with the increase of sample number (**Fig. 1a**), suggesting that our gene catalog covered the vast majority of microbial genes in full-scale BGPs. In addition, we compared the genes assigned to MCA (15,346,132 genes), MCH (9,707,833 genes), MPI (18,662,450 genes), and OTH (15,507,636 genes), and found that most of the genes were shared among the four groups (**Fig. 1b**), which revealed widely existed genes in various types of AD. Moreover, this gene catalog was compared with a previously reported gene set (250,596 genes) derived from a deeply sequenced metagenome of an agricultural BGP (23 Gb metagenomic data, feedstock comprising of 72% maize silage and 28% liquid pig manure) [22], the size of which is much smaller. The reported genes were pairwise aligned with our gene catalog using BLAT [23], with the criteria for shared genes that

identity ≥ 95% and overlap ≥ 90% of the shorter genes. As a result, 165,580 (66.07%) of the reported genes were shared by our gene catalog (0.67%) (**Additional file 3: Fig. S2**). Although a very different feedstock was used in that BGP, there was still a large fraction of genes included in our gene catalog. Additionally, our results emphasized that analyzing one or few BGPs could only provide a limited number of genes, which is far from being complete. Here, we present a first comprehensive microbial gene catalog for AD by using many different digestate samples, however the gene coverage might be further improved by collecting more diversified samples, especially for those rare genes in specific types of AD process.

Taxonomic annotation of the gene catalog

Taxonomic annotation of genes in the gene catalog was performed using CARMA3 [24] on the basis of DIAMOND v0.8.28.90 (DIAMOND, RRID:SCR_016071) [25] alignment against the NCBI-NR database, according to a previously established method [20]. Of the 22,840,185 genes, 76.73% were taxonomically classified at the superkingdom level (**Fig. 2a**). Among these classified genes, 95.95% were assigned to

Bacteria, and the remaining genes were assigned to Archaea (3.03%) and Eukaryota (1.02%). *Firmicutes* (23.04%), *Proteobacteria* (11.22%) and *Bacteroidetes* (9.93%) were the dominant phyla in the gene catalog (**Fig. 2a**), and *Euryarchaeota* (1.78%) was the predominant archaeal phylum, accounting for 76.69% of the archaeal genes. At lower taxonomic levels, only 9.62% and 0.51% of the genes were annotated to specific genera and species, respectively, highlighting the paucity of sequenced genomes of AD microbes in the public databases currently. In addition, genes classified to the methanogens in BGPs include those from *Methanosarcina* (0.16%), *Methanosaeta* (0.14%), *Methanoculleus* (0.14%), *Methanoregula* (0.13%), and *Methanobrevibacter* (0.10%) (**Fig. 2b**). To calculate the relative abundance of different taxonomic ranks (superkingdom, phylum, class, order, family, genus and species), the abundance of the respective genes belonging to each category according to the taxonomic assignments were added.

Functional annotation of the gene catalog

Functional annotation was performed by aligning all protein sequences in the gene

catalog against the KEGG [26] database (release 79) using DIAMOND (v0.8.28.90), and taking the best hit with the criteria of E-value $< 1e-5$. As a result, 57.07% of genes were annotated with KEGG orthologous groups (KOs), with a total number of 13,527 KOs that were comparable to those of the gut microbial gene catalogs of pig and chicken [20, 27]. At the KEGG pathway level, more annotated genes were assigned to carbohydrate metabolism (19.89%), amino acid metabolism (14.61%), energy metabolism (10.52%), metabolism of cofactors and vitamins (10.19%) (**Fig. 3**). In particular, 163 KOs were identified in the methane metabolism pathway, including all KOs involved in all the three methanogenic pathways of acetoclastic, hydrogenotrophic and methylotrophic methanogenesis (**Additional file 4: Fig. S3**). In addition, to analyze the activities of carbohydrate hydrolysis, the genes encoding carbohydrate-active enzymes (CAZymes) were annotated by searching against the dbCAN [28] database (release 5.0) using hmmscan program (HMMER v3.0; HMMER, RRID:SCR_005305) [29] and taking the best hit with the criteria of E-value $< 1e-18$ and coverage > 0.35. A total of 1,607,960 (7.04%) genes were annotated as CAZymes. Based on the functional assignments, relative abundance of CAZymes, KOs, and KEGG functional profiles

were calculated by summing the abundance of the respective genes belonging to each category.

Characterization of core microbial communities in full-scale biogas plants

Identifying the core microbial populations across different full-scale biogas plants is important to understand the essential process in AD, and multiple studies have sought to define the core AD microbiome [9, 30, 31]. In the current study with the in-depth metagenomic sequencing of diverse full-scale BGPs, we found a huge number of common microbes and gene functions shared by all the investigated samples, represented by 400 genera, 6,816 KOs (**Additional file 5: Fig. S4**), accounting for about 98.76% and 99.39% of the total relative abundance of annotated genera and KOs, respectively.

However, the majority of the common microbes were in low abundance, and only a few abundant microbes could be considered as core members play important roles in AD system. Here, we defined core members as the genera that (1) are among the most abundant top 30 bacterial genera and top 5 archaeal genera in each sample and (2) exist

as top microbes in more than 80% of the studied samples. As a result, 13, 15, 12, and 14 genera were identified as core microbes of the group of MCA, MCH, MPI, and OTH, respectively (**Fig. 4**). In particular, seven core genera were shared among all groups, including five bacterial genera of *Bacteroides*, *Clostridium*, *Pseudomonas*, *Sphaerochaeta*, and *Treponema*, and two archaeal genera of *Methanosaeta* and *Methanosarcina* (**Fig. 4**). Among them, the most abundant *Clostridium* and *Bacteroides*, within the phyla of *Firmicutes* and *Bacteroidetes*, are known for their abilities to degrade complex carbohydrates to produce volatile fatty acids (VFAs) [5]. The most abundant archaeal genera of *Methanosarcina* and *Methanosaeta*, within the phylum *Euryarchaeota*, have previously been identified as the dominant methanogens in AD [32, 33].

Microbial functional differentiation among BGPs with different feedstocks

Feedstock is an essential factor that drives microbial community variation in anaerobic digesters [34]. Principal coordinate analysis (PCoA) based on Bray-Curtis dissimilarity at species level were performed by the R package PHYLOSEQ, revealing that digestate

samples were generally separated into three clusters (MCA, MCH and MPI), corresponding to the types of livestock manure (**Fig. 5a**). Microbial diversity (Shannon index) at the genus level also showed distinct differences among the three subgroups, and the microbial diversity of MPI was much higher than those of MCA and MCH (**Additional file 6: Fig. S5**).

Hydrolysis of macromolecular substances is an important step in anaerobic digestion process. To find the functional differences among groups, the relative abundance of genes involved in carbohydrate hydrolysis, protein hydrolysis, VFAs oxidation, and methanogenesis were compared. For genes involved in carbohydrate hydrolysis, we selected the CAZyme families involved in lignocellulose and starch hydrolysis and categorized them in accordance with the CAZy database and previous studies [35-39] (**Additional file 7: Table S2**). The genes involved in protein hydrolysis (with Enzyme Commission number of EC 3.4.x.x) and methanogenesis were selected based on the KO annotation. The genes involved in acetate, propionate and butyrate oxidation pathways were selected according to the KEGG database and a previous study [40] (**Additional file 8: Table S3**).

For genes involved in lignocellulose (cellulose, hemicelluloses, and lignin) degradation, the relative abundances were much higher in MCA than those in MCH and MPI (**Fig. 5b**), which is consistent with the higher content of lignocellulose in cattle manure [41]. In contrast, genes involved in starch hydrolysis have higher relative abundance in MCH and MPI (**Fig. 5b**). Besides, the relative abundance of genes involved in the hydrolysis of proteins was much higher in MCH (**Fig. 5c**), which is consistent with the relatively high protein content of chicken manure [41-43]. VFAs such as acetate, propionate and butyrate, are intermediates in anaerobic digestion process, and the accumulation of VFAs may cause acidification and result in reduced performance of AD process. For the pathway of acetate oxidation, the highest relative gene abundance was observed in MCH (**Fig. 5d**). However, for the pathways of propionate and butyrate oxidation, the relative gene abundance was highest in MCA (**Fig. 5d**). In addition, as one of the most important step of biogas production, the genes involved in methanogenesis were compared, which revealed that MCH has the lowest relative gene abundance (**Fig. 5e**). In summary, the feedstock components have great influence on the process of carbohydrate and protein hydrolysis, VFAs oxidation, and

methanogenesis in BGPs.

Conclusions

Here, we present the first comprehensive microbial gene catalog of anaerobic digestion (AD), by using in-depth sequencing of the digestate samples from 56 full-scale biogas plants (BGPs) treating diverse feedstocks, and provide over 22.8 million taxonomically and functionally annotated genes. Our results confirmed the existence of core microbiome in AD, and showed that the type of feedstock (cattle, chicken and pig manure) has a great influence on carbohydrate and protein hydrolysis, VFAs oxidation, and methanogenesis. Compared to the published microbial gene catalogs of different ecosystems such as soil, ocean, animal gut and rumen [20, 27, 44-47], biogas plants are man-made extremely anaerobic ecosystems where AD is performed by a complex consortium of anaerobic bacterial and archaeal genes. Hence, our gene catalog will not only serve as a useful reference database for quick analyses of AD microbiome data, but also provide a huge number of microbial gene resources for the study and utilization of anaerobic microbiota.

Availability of supporting data and materials

All raw sequencing data generated during the current study have been deposited at DDBJ/ENA/GenBank under project accession PRJNA533495. For detail, SRR8925713 ~ SRR8925730, SRR8925732 ~ SRR8925742, SRR8925747 ~ SRR8925748, SRR8925751 ~ SRR8925758, SRR8925797 ~ SRR8925806, SRR8925817 ~ SRR8925824, and SRR8925826 ~ SRR8925827 for metagenome sequencing data of 59 digestate samples. Other supporting data, including the gene catalog of 22.8 million genes, taxonomic and functional annotation files of the gene catalog, and the abundance profile tables generated in this study are available in a temporary FTP site [48].

Declarations

List of abbreviations

ABR: anaerobic baffled reactor; AD: anaerobic digestion; BGP: biogas plant; CAZyme: carbohydrate-active enzyme; CSTR: continuous stirred tank reactor; Gb: gigabase; KO:

KEGG orthologous group; MCA: cattle manure biogas plants; MCH: chicken manure biogas plants; MPI: pig manure biogas plants; OTH: biogas plants with other feedstocks; PCoA: principal coordinate analysis; USR: upflow solids reactor; VFA: volatile fatty acid.

Consent for publication

Not applicable.

Competing interests

The authors declare that they have no competing interests.

Funding

This project was supported by grants from Shenzhen science and technology program (JCYJ20190814163805604), Agricultural Science and Technology Innovation Program (ASTIP), Chinese Academy of Agricultural Sciences (CAAS-ASTIP-2016-BIOMA), the Agricultural Science and Technology Innovation Program && The Elite

Young Scientists Program of CAAS, Fundamental Research Funds for Central Non-profit Scientific Institution (No. Y2017JC01), Science and Technology Program of Sichuan Province, China (2017JY0242), the Agricultural Science and Technology Innovation Program Cooperation and Innovation Mission (CAAS-XTCX2016), the Fund of Key Laboratory of Shenzhen (ZDSYS20141118170111640), the Fundamental Research Funds for Central Non-profit Scientific Institution, China (1610012016023) and the Infrastructure and Facility Development Program of Sichuan Province (2019JDPT0012). The sponsors had no role in design or conduct of the study; the collection, management, analysis, or interpretation of the data; the preparation, review, or approval of the manuscript; or the decision to submit the manuscript for publication.

Authors' contributions

SM, YH, HF, and QL collected the samples, and FJ, YZ, LY, and SL extracted the DNA and constructed the Illumina sequencing libraries. SM, FJ, YH, YZ, SW, BL, and HW analyzed the data. HL and YR provide helpful suggestions. SM, FJ, YH, YZ, and SW wrote the raw manuscript. WF, YD, and LC conceived the study, designed the

experiments, and revised the manuscript. All authors read and approved the final manuscript.

Acknowledgements

We thank Jing He, Yunfei Zhang, Yanlai Liu, Xia Li, Bo Tu, Shouchao Lai, Nengmin Zhu, Lirong Dai, Lu Yang, Yinggang Zhang from Biogas Institute of Ministry of Agricultural and Rural Affairs for collecting samples. We also express our thanks to Jianjun Hu, Ling Qiu, Zuojun Liu, Liumeng Chen, Xiaomei Ye for their assistance with sample collection.

References

1. Tyagi VK and Lo SL. Sludge: A waste or renewable source for energy and resources recovery? Renew Sust Energ Rev. 2013;25:708-28.
2. Stolze Y, Bremges A, Rumming M, Henke C, Maus I, Pühler A, et al. Identification and genome reconstruction of abundant distinct taxa in microbiomes from one thermophilic and three mesophilic production-scale biogas plants. Biotechnol Biofuels. 2016;9:156.
3. Luo G, Fotidis IA, and Angelidaki I. Comparative analysis of taxonomic, functional, and metabolic patterns of microbiomes from 14 full-scale biogas

reactors by metagenomic sequencing and radioisotopic analysis. Biotechnol Biofuels. 2016;9:51.

4. Angenent LT, Karim K, Al-Dahhan MH, Wrenn BA, and Domiguez-Espinosa R. Production of bioenergy and biochemicals from industrial and agricultural wastewater. Trends Biotechnol. 2004;22:477-85.

5. Hassa J, Maus I, Off S, Pühler A, Scherer P, Klocke M, et al. Metagenome, metatranscriptome, and metaproteome approaches unraveled compositions and functional relationships of microbial communities residing in biogas plants. Appl Microbiol Biotechnol. 2018;102:5045-63.

6. Schnürer A. Biogas production: microbiology and technology. Adv Biochem Eng Biotechnol. 2016;156:195-234.

7. Narihiro T, Nobu MK, Kim NK, Kamagata Y, and Liu WT. The nexus of syntrophy-associated microbiota in anaerobic digestion revealed by long-term enrichment and community survey. Environ Microbiol. 2015;17:1707-20.

8. De Vrieze J, Saunders AM, He Y, Fang J, Nielsen PH, Verstraete W, et al. Ammonia and temperature determine potential clustering in the anaerobic digestion microbiome. Water Res. 2015;75:312-23.

9. Mei R, Nobu MK, Narihiro T, Kuroda K, Munoz Sierra J, Wu Z, et al. Operation-driven heterogeneity and overlooked feed-associated populations in global anaerobic digester microbiome. Water Res. 2017;124:77-84.

10. Jia Y, Ng SK, Lu H, Cai M, and Lee PKH. Genome-centric metatranscriptomes and ecological roles of the active microbial populations during cellulosic biomass anaerobic digestion. Biotechnol Biofuels. 2018;11:117.

11. Treu L, Kougias PG, Campanaro S, Bassani I, and Angelidaki I. Deeper insight into the structure of the anaerobic digestion microbial community; the biogas

microbiome database is expanded with 157 new genomes. Bioresour Technol. 2016;216:260-6.

12. Campanaro S, Treu L, Kougias PG, Luo G, and Angelidaki I. Metagenomic binning reveals the functional roles of core abundant microorganisms in twelve full-scale biogas plants. Water Res. 2018;140:123-34.

13. Campanaro S, Treu L, Kougias PG, De Francisci D, Valle G, and Angelidaki I. Metagenomic analysis and functional characterization of the biogas microbiome using high throughput shotgun sequencing and a novel binning strategy. Biotechnol Biofuels. 2016;9:26.

14. Clean_adapter and clean_lowqul on github. https://github.com/fanagislab/DBG_assembly/tree/master/clean_illumina

15. Li DH, Luo RB, Liu CM, Leung CM, Ting HF, Sadakane K, et al. MEGAHIT v1.0: A fast and scalable metagenome assembler driven by advanced methodologies and community practices. Methods. 2016;102:3-11.

16. Hyatt D, LoCascio PF, Hauser LJ, and Uberbacher EC. Gene and translation initiation site prediction in metagenomic sequences. Bioinformatics. 2012;28:2223-30.

17. Qin J, Li R, Raes J, Arumugam M, Burgdorf KS, Manichanh C, et al. A human gut microbial gene catalogue established by metagenomic sequencing. Nature. 2010;464:59-65.

18. Li H and Durbin R. Fast and accurate short read alignment with Burrows-Wheeler transform. Bioinformatics. 2009;25:1754-60.

19. Fu LM, Niu BF, Zhu ZW, Wu ST, and Li WZ. CD-HIT: accelerated for clustering the next-generation sequencing data. Bioinformatics. 2012;28:3150-52.

20. Huang P, Zhang Y, Xiao KP, Jiang F, Wang HC, Tang DZ, et al. The chicken gut metagenome and the modulatory effects of plant-derived benzylisoquinoline alkaloids. Microbiome. 2018;6:211.
21. Qin JJ, Li YR, Cai ZM, Li SH, Zhu JF, Zhang F, et al. A metagenome-wide association study of gut microbiota in type 2 diabetes. Nature. 2012;490:55-60.
22. Bremges A, Maus I, Belmann P, Eikmeyer F, Winkler A, Albersmeier A, et al. Deeply sequenced metagenome and metatranscriptome of a biogas-producing microbial community from an agricultural production-scale biogas plant. Gigascience. 2015;4:33.
23. Kent WJ. BLAT--the BLAST-like alignment tool. Genome Res. 2002;12:656-64.
24. Gerlach W and Stoye J. Taxonomic classification of metagenomic shotgun sequences with CARMA3. Nucleic Acids Res. 2011;39:e91.
25. Buchfink B, Xie C, and Huson DH. Fast and sensitive protein alignment using DIAMOND. Nat Methods. 2015;12:59-60.
26. Kanehisa M, Goto S, Kawashima S, Okuno Y, and Hattori M. The KEGG resource for deciphering the genome. Nucleic Acids Res. 2004;32:D277-D80.
27. Xiao L, Estelle J, Kiilerich P, Ramayo-Caldas Y, Xia ZK, Feng Q, et al. A reference gene catalogue of the pig gut microbiome. Nat Microbiol. 2016;1:16161.
28. Yin YB, Mao XZ, Yang JC, Chen X, Mao FL, and Xu Y. dbCAN: a web resource for automated carbohydrate-active enzyme annotation. Nucleic Acids Res. 2012;40:W445-W51.
29. Eddy SR. Accelerated Profile HMM Searches. Plos Comput Biol. 2011;7:e1002195.

30. Mei R, Narihiro T, Nobu MK, Kuroda K, and Liu WT. Evaluating digestion efficiency in full-scale anaerobic digesters by identifying active microbial populations through the lens of microbial activity. Scientific Reports. 2016;6:34090.

31. Calusinska M, Goux X, Fossepre M, Muller EEL, Wilmes P, and Delfosse P. A year of monitoring 20 mesophilic full-scale bioreactors reveals the existence of stable but different core microbiomes in bio-waste and wastewater anaerobic digestion systems. Biotechnol Biofuels. 2018;11:196.

32. Anne C, H David S, and John F. Growth kinetics and competition between *Methanosarcina* and *Methanosaeta* in mesophilic anaerobic digestion. Water Environ Res. 2006;78:486-96.

33. Zhang L, Loh KC, Lim JW, and Zhang JX. Bioinformatics analysis of metagenomics data of biogas-producing microbial communities in anaerobic digesters: A review. Renew Sust Energ Rev. 2019;100:110-26.

34. Zhang W, Werner JJ, Agler MT, and Angenent LT. Substrate type drives variation in reactor microbiomes of anaerobic digesters. Bioresour Technol. 2014;151:397-401.

35. Artzi L, Bayer EA, and Moraïs S. Cellulosomes: bacterial nanomachines for dismantling plant polysaccharides. Nat Rev Microbiol. 2016;15:83-95.

36. Gharechahi J and Salekdeh GH. A metagenomic analysis of the camel rumen's microbiome identifies the major microbes responsible for lignocellulose degradation and fermentation. Biotechnol Biofuels. 2018;11:216.

37. Kougias PG, Campanaro S, Treu L, Tsapekos P, Armani A, and Angelidaki I. Spatial distribution and diverse metabolic functions of lignocellulose-degrading uncultured bacteria as revealed by genome-centric metagenomics. Appl

Environ Microbiol. 2018;84:e01244-18.

38. Liu N, Li H, Chevrette MG, Zhang L, Cao L, Zhou H, et al. Functional metagenomics reveals abundant polysaccharide-degrading gene clusters and cellobiose utilization pathways within gut microbiota of a wood-feeding higher termite. ISME J. 2019;13:104-17.

39. Zhu N, Yang J, Ji L, Liu J, Yang Y, and Yuan H. Metagenomic and metaproteomic analyses of a corn stover-adapted microbial consortium EMSD5 reveal its taxonomic and enzymatic basis for degrading lignocellulose. Biotechnol Biofuels. 2016;9:243.

40. Mosbaek F, Kjeldal H, Mulat DG, Albertsen M, Ward AJ, Feilberg A, et al. Identification of syntrophic acetate-oxidizing bacteria in anaerobic digesters by combined protein-based stable isotope probing and metagenomics. ISME J. 2016;10:2405-18.

41. Wang M, Li W, Li P, Yan S, and Zhang Y. An alternative parameter to characterize biogas materials: Available carbon-nitrogen ratio. Waste Manag. 2017;62:76-83.

42. Sheu SY, Liu LP, and Chen WM. *Novosphingobium bradum* sp. nov., isolated from a spring. Int J Syst Evol Microbiol. 2016;66:5083-90.

43. Zakharyuk A, Kozyreva L, Ariskina E, Troshina O, Kopitsyn D, and Shcherbakova V. *Alkaliphilus namsaraevii* sp. nov., an alkaliphilic iron- and sulfur-reducing bacterium isolated from a steppe soda lake. Int J Syst Evol Microbiol. 2017;67:1990-95.

44. Li J, Zhong H, Ramayo-Caldas Y, Terrapon N, Lombard V, Potocki-Veronese G, et al. A catalog of microbial genes from the bovine rumen unveils a specialized and diverse biomass-degrading environment. Gigascience.

2020;9:1-15.
45. Bahram M, Hildebrand F, Forslund SK, Anderson JL, Soudzilovskaia NA, Bodegom PM, et al. Structure and function of the global topsoil microbiome. Nature. 2018;560:233-37.
46. Sunagawa S, Coelho LP, Chaffron S, Kultima JR, Labadie K, Salazar G, et al. Ocean plankton. Structure and function of the global ocean microbiome. Science. 2015;348:1261359.
47. Li J, Jia H, Cai X, Zhong H, Feng Q, Sunagawa S, et al. An integrated catalog of reference genes in the human gut microbiome. Nat Biotechnol. 2014;32:834-41.
48. Ma SC, Jiang F, Huang Y, Zhang Y, et al. Supporting data for "A microbial gene catalog of anaerobic digestion from full-scale biogas plants. ". Temporary FTP site: ftp://ftp.agis.org.cn/~fanwei/Anaerobic_digestion_metagenome

Figure legends and supplementary files

Fig. 1 The constructed microbial gene catalog of anaerobic digestion. **a** Rarefaction curve of detected genes from the whole set of 59 digestate samples. The curve approaches saturation as sample number increases. The gene number of a given number of samples was calculated after 100 random samplings with replacement and plotted with a box plot. Boxplots show the median ± interquartile range (IQR) and 1.5 IQR ranges (whiskers), with outliers denoted by circles. **b** Venn diagram of shared genes

among four groups of non-redundant genes from MCA, MCH, MPI and OTH. Only a small proportion of genes were unique for each group.

Fig. 2 Taxonomic annotation of the gene catalog. **a** Taxonomic annotation of the gene catalog at the superkingdom and phylum levels. A total of 73.63% and 2.32% of genes in the gene catalog were assigned to Bacteria and Archaea, respectively. **b** Percentage of genes assigned to the top 10 methanogenic archaea at genus level.

Fig. 3 KEGG functional profile of the gene catalog. Genes without functional annotations were excluded.

Fig. 4 Distributions of feedstock-associated core genera among four group of MCA, MCH, MPI, and OTH. The area of each circle represents the median value of relative abundance of the corresponding genus in each group, and the non-core genera were not presented. “Core microbes” were defined as the genera that (1) are among the top 30 bacterial genera and top 5 archaeal genera in each sample and (2) exist as top microbes

in more than 80% of the samples in each group.

Fig. 5 Comparisons of taxonomic and functional profiles among different biogas plants (BGPs). **a** Principal coordinate analysis (PCoA) based on Bray-Curtis dissimilarity at the species level. The digestate samples were separated into three clusters (MCA, MCH and MPI). MCA, cattle manure BGPs; MCH, chicken manure BGPs; MPI, pig manure BGPs. **b** Relative abundance of genes involved in the hydrolysis of starch, oligosaccharide, polysaccharide, and lignocellulose (lignin, hemicellulose, and cellulose) hydrolysis. **c** Relative abundance of genes involved in protein hydrolysis. **d** Relative abundance of genes involved in acetate, propionate, and butyrate oxidation. **e** Relative abundance of genes involved in methanogenesis. Boxplots show the median ± interquartile range (IQR) and 1.5 IQR ranges (whiskers), with outliers denoted by circles.

Additional file 1: Fig. S1 Geographic distribution of 56 full-scale biogas plants (BGPs) from which the digestate samples were collected. The sampling BGPs ranged in

location from the Northeast (45°27' N，131°36' E) to the Southwest (23°21' N, 103°20' E) China, including cattle manure BGPs (MCA), chicken manure BGPs (MCH), pig manure BGPs (MPI), and BGPs with other feedstocks (OTH). (PDF 5500K)

Additional file 2: Table S1 Background information of the investigated 56 full-scale biogas plants (BGPs). (XLSX 23K)

Additional file 3: Fig. S2 Genes shared between our gene catalog and gene set reported by Bremges et al. (2015). (PDF 1600K)

Additional file 4: Fig. S3 The KEGG methane metabolism pathway. The enzymes present in 100% of digestate samples (59 samples) were highlighted in red, the enzymes present in more than 90% of digestate samples were highlighted in light blue, and other enzymes annotated in the gene catalog were shown in green. The enzymes analyzed based on the KO annotation. (PDF 962K)

Additional file 5: Fig. S4 The number of shared genera and KOs among biogas plants (BGPs) at different frequency thresholds. (PDF 873K)

Additional file 6: Fig. S5 Shannon index of MCA, MCH and MPI at the genus level. MCA, cattle manure biogas plants (BGPs); MCH, chicken manure BGPs; MPI, pig manure BGPs. Boxplots show median ± interquartile range (IQR) and 1.5 IQR ranges (whiskers), with outliers denoted by circles. (PDF 852K)

Additional file 7: Table S2 Categories of CAZyme families. (XLSX 13K)

Additional file 8: Table S3 Genes selected for the analysis of the acetate, propionate and butyrate oxidation pathways. (XLSX 13K)

Figure 1

Click here to access/download;Figure;Figure 1.pdf

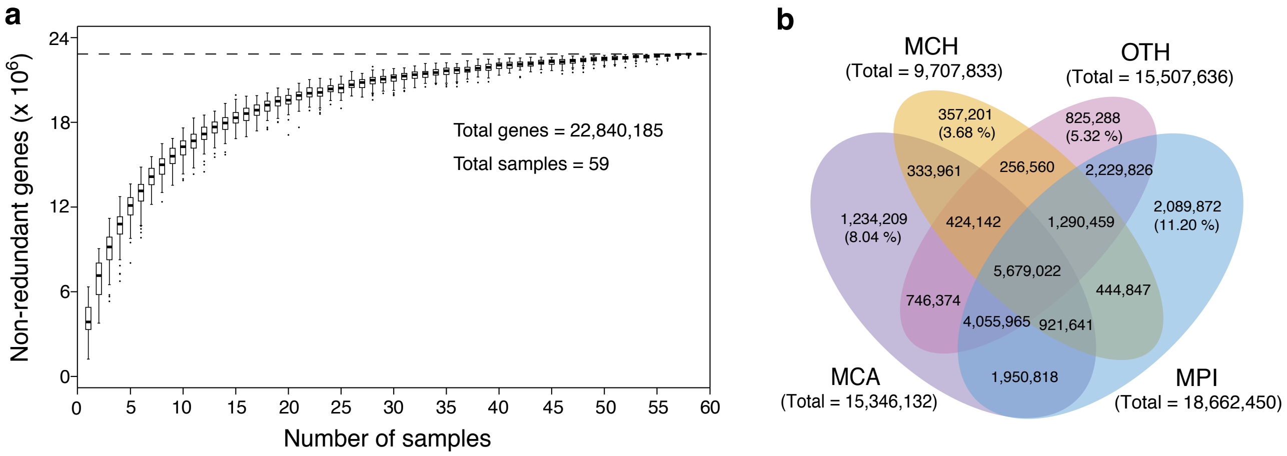

Figure 2

Click here to access/download;Figure;Figure 2.pdf

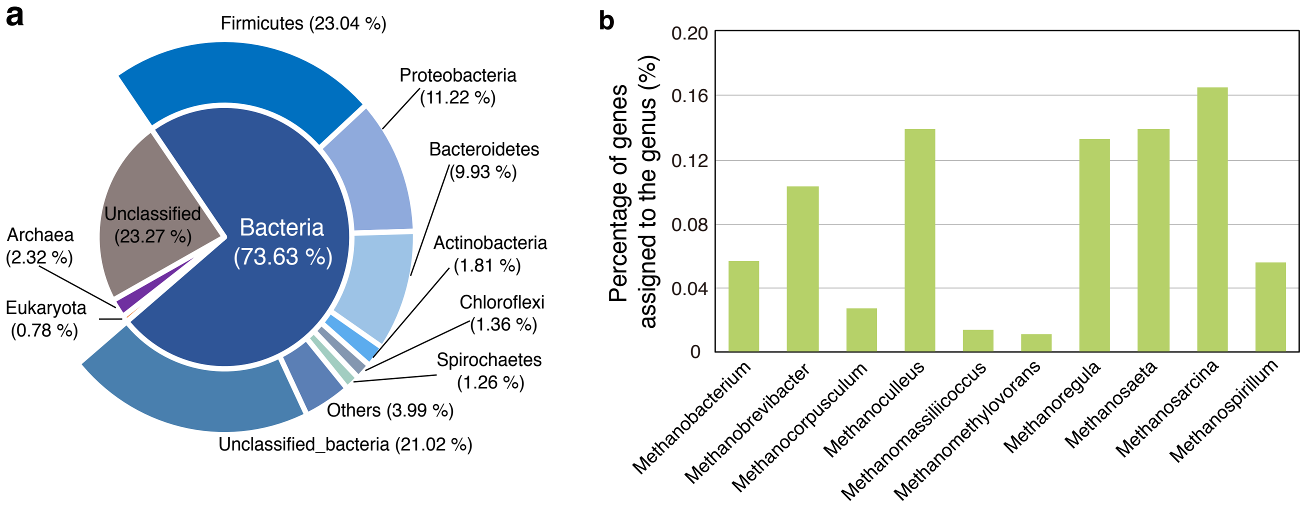

 Click here to access/download;Figure;Figure 3.pdf

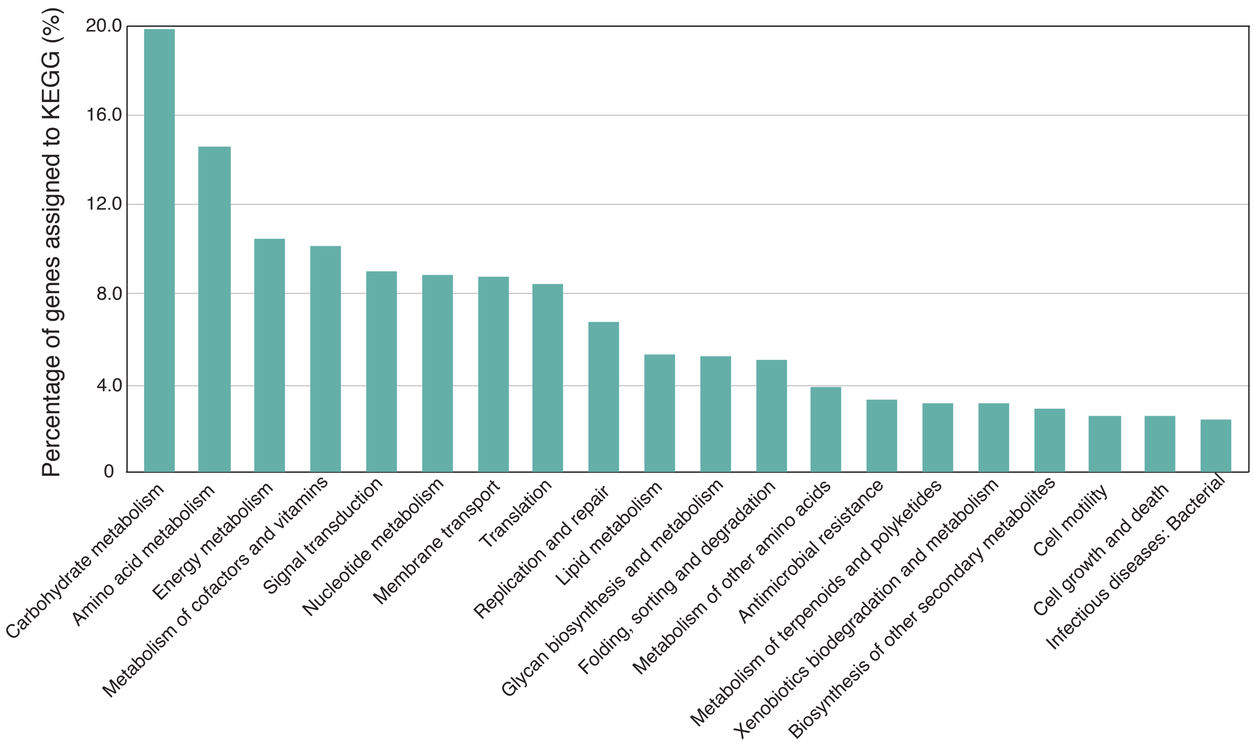

Figure 4

Click here to access/download;Figure;Figure 4.pdf

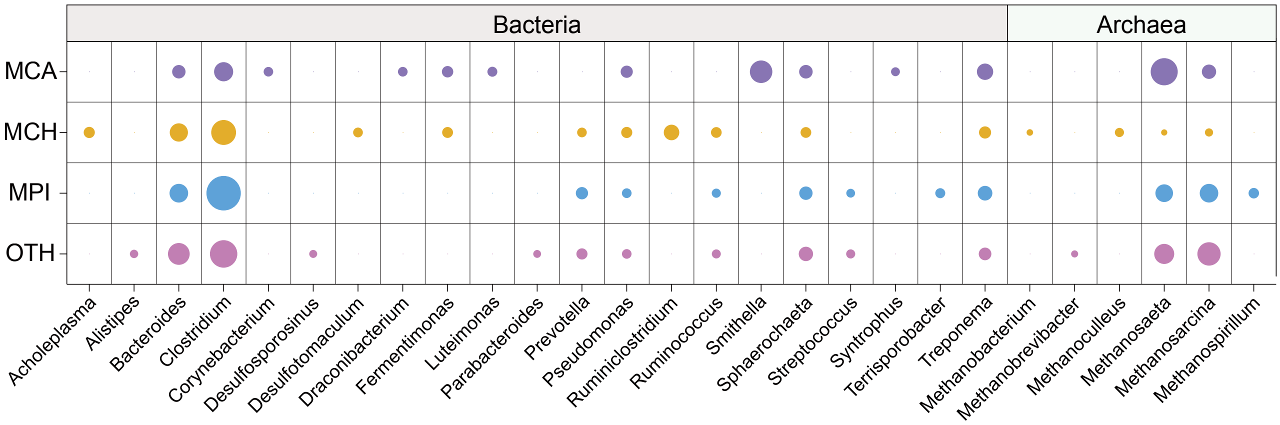

Figure 5

Click here to access/download;Figure;Figure 5.pdf

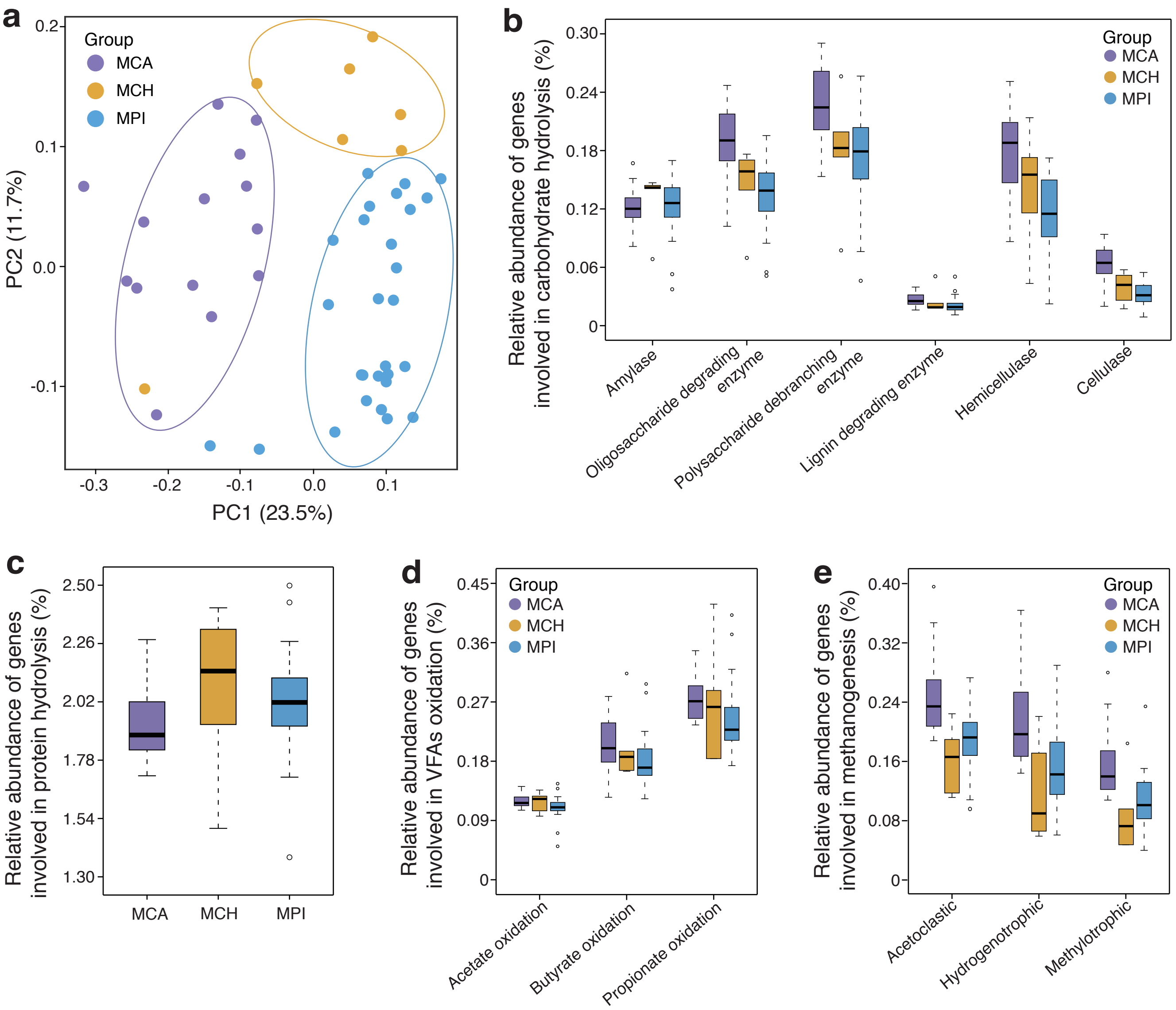

Additional file 1

Click here to access/download
Supplementary Material
Additional file 1-Fig. S1.pdf

Additional file 2

Click here to access/download
Supplementary Material
Additional file 2-Table S1.xlsx

Additional file 3

Click here to access/download
Supplementary Material
Additional file 3-Fig. S2.pdf

Additional file 4

Click here to access/download
Supplementary Material
Additional file 4-Fig. S3.pdf

Additional file 5

Click here to access/download
Supplementary Material
Additional file 5-Fig. S4.pdf

Additional file 6

Click here to access/download
Supplementary Material
Additional file 6-Fig. S5.pdf

Additional file 7

Click here to access/download
Supplementary Material
Additional file 7-Table S2.xlsx

Additional file 8

Click here to access/download
Supplementary Material
Additional file 8-Table S3.xlsx
