# Supplementary material for: A microbial gene catalog of anaerobic digestion from full-scale biogas plants
Source: Gigascience. 2021 Jan 27;10(1):giaa164. doi: 10.1093/gigascience/giaa164 (PMC7842101; doi:10.1093/gigascience/giaa164)
Supplement: giaa164_GIGA-D-20-00207_Revision_3 [file giaa164_giga-d-20-00207_revision_3.pdf]

GigaScience

A microbial gene catalog of anaerobic digestion from full-scale biogas plants

--Manuscript Draft--

Manuscript Number:	GIGA-D-20-00207R2
Full Title:	A microbial gene catalog of anaerobic digestion from full-scale biogas plants
Article Type:	Data Note
Funding Information:	Infrastructure and Facility Development Program of Sichuan Province (2019JDPT0012) — Dr Yu Deng<br>Shenzhen science and technology program (JCYJ20190814163805604) — Dr Wei Fan<br>Agricultural Science and Technology Innovation Program (ASTIP), Chinese Academy of Agricultural Sciences (CAAS-ASTIP-2016-BIOMA) — Dr Yu Deng<br>Agricultural Science and Technology Innovation Program && The Elite Young Scientists Program of CAAS (None) — Dr Wei Fan<br>Fundamental Research Funds for Central Non-profit Scientific Institution (Y2017JC01) — Dr Wei Fan<br>Science and Technology Program of Sichuan Province, China (2017JY0242) — Dr Shichun Ma<br>Agricultural Science and Technology Innovation Program Cooperation and Innovation Mission (CAAS-XTCX2016) — Dr Wei Fan<br>Fund of Key Laboratory of Shenzhen (ZDSYS20141118170111640) — Not applicable<br>Fundamental Research Funds for Central Non-profit Scientific Institution, China (1610012016023) — Dr Shichun Ma
Abstract:	Background: Biogas production with anaerobic digestion (AD) is one of the most promising solutions for both renewable energy production and resolving the environmental problem caused by the increase in organic wastes worldwide. However, the complex structure of the microbiome in AD is less understood.<br>Findings: In this study, we constructed a microbial gene catalog of AD (22,840,185 genes), based on 1,817 gigabase (Gb) metagenomic data, derived from digestate samples of 56 full-scale biogas plants fed with diverse feedstocks. Among the gene catalog, 73.63% and 2.32% of genes were taxonomically annotated to Bacteria and Archaea, respectively and 57.07% of genes were functionally annotated with KEGG orthologous groups. Our results confirmed the existence of core microbiome in AD, and showed that the type of feedstock (cattle, chicken and pig manure) has a great influence on carbohydrate hydrolysis and methanogenesis. In addition, 2,426 metagenome-assembled genomes (MAGs) were recovered from all digestate samples, and all genomes were estimated to be ≥ 80% complete ≤ 10% contamination.<br>Conclusions: This study deepens our understanding of microbial compositions and functions in AD process, and alsoprovides a huge number of reference genome and gene resources for analysis of anaerobic microbiota.
Corresponding Author:	Wei Fan<br>Chinese Academy of Agricultural Sciences<br>CHINA
Corresponding Author Secondary Information:	
Corresponding Author's Institution:	Chinese Academy of Agricultural Sciences
Corresponding Author's Secondary	

Powered by Editorial Manager® and ProduXion Manager® from Aries Systems Corporation

**Institution:**	
**First Author:**	Shichun Ma
**First Author Secondary Information:**	
**Order of Authors:**	Shichun Ma
	Fan Jiang
	Yan Huang
	Yan Zhang
	Sen Wang
	Hui Fan
	Bo Liu
	Qiang Li
	Lijuan Yin
	Hengchao Wang
	Hangwei Liu
	Yuwei Ren
	Shuqu Li
	Lei Cheng
	Wei Fan
	Yu Deng
**Order of Authors Secondary Information:**	
**Response to Reviewers:**	Response to the comments of the Editor and reviewers<br>Thank you for sending us the feedback on our manuscript A microbial gene catalog of anaerobic digestion from full-scale biogas plants [Paper # GIGA-D-20-00207].<br>We are now submitting a new version to address the remaining concerns of reviewers. Followings are our specific responses:<br><br>Response to Reviewer #1:<br>I would like to thank the authors for addressing all of my points in there response to reviewers document this made the process of review much easier.<br><br>I can confirm that the revisions do address all of my concerns and I am happy to now recommend this manuscript for acceptance.<br><br>The document would benefit from a fresh set of eyes checking over the wording and grammar and I list here a couple of examples that could be made easier to read/understand:<br><br>(line 55)<br>"In the context of global climate change, biogas as a renewable energy form has become increasingly attractive to the world's attention in recent years." - > "In the context of global climate change, biogas as a renewable energy form has increasingly drawn the world's attention in recent years.<br>Response: We have revised the sentence according to reviewer's suggestion to "In the context of global climate change, biogas as a renewable energy form has increasingly drawn the world's attention in recent years". (page 4)<br><br>(line 134)<br>The integrity of DNA extracts was checked on 0.7% (w/v) agarose gel with GelRed nucleic acid gel stain (cat. no. 41003; Biotium, USA), and DNA samples with obvious concentrated DNA band and the fragment length of the band > 15 kb were used for further analysis (Additional file 3: Fig S2).<br>->

The integrity of DNA extracts was checked on 0.7% (w/v) agarose gel with GelRed nucleic acid gel stain (cat. no. 41003; Biotium, USA). DNA samples showing obvious concentrated DNA bands >15 kb in size were used for further analysis (Additional file 3: Fig S2).
Response: We have revised the sentence according to reviewer's suggestion to "The integrity of DNA extracts was checked on 0.7% (w/v) agarose gel with GelRed nucleic acid gel stain (cat. no. 41003; Biotium, USA). DNA samples showing obvious concentrated DNA bands > 15 kb in size were used for further analysis". (page 9)

In addition, we have made other revisions about the wording and grammar:

1. (line 63) Anaerobic digestion includes four sequential metabolic steps, namely hydrolysis, acidogenesis, acetogenesis, and methanogenesis, and are performed by a complex consortium of bacteria and archaea [4, 5].
->
Anaerobic digestion includes four sequential metabolic steps, namely hydrolysis, acidogenesis, acetogenesis, and methanogenesis, and is performed by a complex consortium of bacteria and archaea [4, 5]. (page 4-5)

2. (line 138) After DNA quality checks, the three replicates of high-quality DNA (band length > 15 kb, A260/280 1.8-2.0, dsDNA concentration > 20 ng/μL) of each sample was pooled for library construction.
->
After DNA quality checks, the three replicates of high-quality DNA (band length > 15 kb, A260/280 1.8-2.0, dsDNA concentration > 20 ng/μL) of each sample were pooled for library construction. (page 9)

3. (line 330) Besides, the relative abundance of genes involved in the hydrolysis of proteins was much higher in MCH (Fig. 5c), which is consistent with the relatively high protein content of chicken manure [45-47].
->
Besides, the relative abundance of genes involved in the hydrolysis of proteins was much higher in MCH (Fig. 5c), which is associated with the relatively high protein content of chicken manure [45-47]. (page 21)

4. (line 346) In addition, various parameters in AD also have important effects on shaping microbial communities, and several process parameters (operation temperature; pH; hydraulic retention time, HRT, and reactor volume), physicochemical characteristics of feedstock (total nitrogen, TN; total carbon, TC; and total solid, TS) and intermediate metabolites (total ammonia nitrogen, TAN; and VFAs) for all BGPs (Additional file 2: Table S1) from the groups MCA, MCH, MPI, and OTH were analyzed.
->
In addition, various parameters in AD also have important effects on shaping microbial communities. Several process parameters (operation temperature; pH; hydraulic retention time, HRT, and reactor volume), physicochemical characteristics of feedstock (total nitrogen, TN; total carbon, TC; and total solid, TS) and intermediate metabolites (total ammonia nitrogen, TAN; and VFAs) for all BGPs (Additional file 2: Table S1) from the groups MCA, MCH, MPI, and OTH were analyzed. (page 22-23)

5. (line 352) Redundancy analysis (RDA) at the genus level revealed that operation temperature and TAN were primarily determinant parameters that influenced the microbial composition, and then followed by TS, acetate, total VFAs, acetate, TN, and pH (Additional file 12: Fig. S7). The result was consistent with a previous study that TAN and digester temperature were identified as the main contributing factors to cluster formation [8].
->
Redundancy analysis (RDA) at the genus level revealed that operation temperature and TAN were primarily determinant parameters that influenced the microbial composition, and then followed by TS, acetate, total VFAs, TN, and pH (Additional file 12: Fig. S7). The result was agreed with a previous finding that TAN and digester temperature were identified as the main contributing factors to cluster formation [8]. (page 23)

6. (line 407) In addition, we also provided 2,426 MAGs derived from full-scale biogas

Powered by Editorial Manager® and ProduXion Manager® from Aries Systems Corporation

	plants.<br>-> Additionally, we also provided 2,426 MAGs derived from full-scale biogas plants. (page 26)
**Additional Information:**	
**Question**	**Response**
Are you submitting this manuscript to a special series or article collection?	No
**Experimental design and statistics**<br><br>Full details of the experimental design and statistical methods used should be given in the Methods section, as detailed in our Minimum Standards Reporting Checklist. Information essential to interpreting the data presented should be made available in the figure legends.<br><br>Have you included all the information requested in your manuscript?	Yes
**Resources**<br><br>A description of all resources used, including antibodies, cell lines, animals and software tools, with enough information to allow them to be uniquely identified, should be included in the Methods section. Authors are strongly encouraged to cite Research Resource Identifiers (RRIDs) for antibodies, model organisms and tools, where possible.<br><br>Have you included the information requested as detailed in our Minimum Standards Reporting Checklist?	Yes
**Availability of data and materials**<br><br>All datasets and code on which the conclusions of the paper rely must be either included in your submission or deposited in publicly available repositories (where available and ethically appropriate), referencing such data using a unique identifier in the references and in	Yes

the “Availability of Data and Materials” section of your manuscript.<br><br>Have you have met the above requirement as detailed in our Minimum Standards Reporting Checklist?	

Powered by Editorial Manager® and ProduXion Manager® from Aries Systems Corporation

Manuscript

Click here to access/download;Manuscript;Manuscript-Gigascience(1).docx

A microbial gene catalog of anaerobic digestion from full-scale biogas plants

Shichun Ma[1,3]*, Fan Jiang[2]*, Yan Huang[1,3]*, Yan Zhang[2], Sen Wang[2], Hui Fan[1,3], Bo liu[2], Qiang Li[1,3], Lijuan Yin[2], Hengchao Wang[2], Hangwei Liu[2], Yuwei Ren[2], Shuqu Li[2], Lei Cheng[1,3], Wei Fan[2]†, and Yu Deng[1,3]†

Affiliations:

[1]Biogas Institute of Ministry of Agricultural and Rural Affairs, Chengdu, Sichuan, 610041, China.

[2]Guangdong Laboratory for Lingnan Modern Agriculture (Shenzhen Branch), Genome Analysis Laboratory of the Ministry of Agriculture and Rural Affairs, Agricultural Genomics Institute at Shenzhen, Chinese Academy of Agricultural Sciences, Shenzhen, Guangdong, 518120, China.

[3]Laboratory of Development and Application of Rural Renewable Energy, Ministry of Agricultural and Rural Affairs, Chengdu, Sichuan, 610041, China.

Shichun Ma: mashichun@caas.cn; Fan Jiang: greatjf@163.com; Yan Huang: huangyan01@caas.cn; Yan Zhang: milrazhang@163.com; Sen Wang: wangsen1993@163.com; Hui Fan: fanhui01@caas.cn; Bo Liu: lb_bobo@aliyun.com; Qiang Li: liqiang03@caas.cn; Lijuan Yin: yinlijuan1005@163.com; Hengchao Wang: wanghengchao000@qq.com; Hangwei Liu: liuhangwei2014@163.com; Yuwei Ren: xiaoshudaxia@126.com; Shuqu Li: lishuqu1234@163.com; Lei Cheng: chenglei@caas.cn.

*These authors contributed equally to this work.

†Corresponding Authors: Wei Fan and Yu Deng

E-mail: fanwei@caas.cn and dengyu@caas.cn

ORCID details:

Ma, Shichun [0000-0003-1608-9970]; Jiang, Fan [0000-0003-1359-0970]; Huang, Yan [0000-0001-5822-5585]; Zhang, Yan [0000-0003-2281-7807]; Wang, Sen [0000-0001-9793-4472]; Fan, Hui [0000-0003-0104-2870]; Liu, Bo [0000-0002-7840-9450]; Li, Qiang [0000-0001-9700-1429]; Yin, Lijuan [0000-0003-2678-9719]; Wang, Hengchau [0000-0002-8754-4195]; Liu, Hangwei [0000-0002-4931-1307]; Ren, Yuwei [0000-0002-5548-4490]; Li, Shuqu [0000-0003-3199-7029]; Cheng, Lei [0000-0003-1178-8190]; Fan, Wei [0000-0001-5036-8733]; Deng, Yu [0000-0003-3467-4540]

Abstract

Background: Biogas production with anaerobic digestion (AD) is one of the most promising solutions for both renewable energy production and resolving the environmental problem caused by the worldwide increase in organic waste. However, the complex structure of the microbiome in AD is currently poorly understood.

Findings: In this study, we constructed a microbial gene catalog of AD (22,840,185 genes), based on 1,817 gigabase (Gb) metagenomic data, derived from digestate samples of 56 full-scale biogas plants fed with diverse feedstocks. Among the gene catalog, 73.63% and 2.32% of genes were taxonomically annotated to Bacteria and Archaea, respectively and 57.07% of genes were functionally annotated with KEGG orthologous groups. Our results confirmed the existence of core microbiome in AD, and showed that the type of feedstock (cattle, chicken and pig manure) has a great influence on carbohydrate hydrolysis and methanogenesis. In addition, 2,426 metagenome-assembled genomes (MAGs) were recovered from all digestate samples, and all genomes were estimated to be ≥ 80% complete ≤ 10% contamination.

Conclusions: This study deepens our understanding of the microbial composition and

function in the AD process, and also provides a huge number of reference genome and gene resources for analysis of anaerobic microbiota.

Keywords: Anaerobic digestion, metagenome, manure waste, full-scale biogas plant, metagenome-assembled genomes, methanogenesis

Background

In the context of global climate change, in recent years the use of biogas as a renewable form of energy has increasingly drawn the world's attention. While the vast amount of organic waste caused by population expansion, urbanization expansion and agriculture intensification continues to severely threaten the environment [1]. At the same time, anaerobic digestion (AD) of biomass is considered as one of the most important solutions for both producing renewable energy and resolving the problem of organic waste, such as animal manure, crop residues and wastewater sludge [2, 3], and to date has been applied worldwide.

Anaerobic digestion includes four sequential metabolic steps, namely hydrolysis, acidogenesis, acetogenesis, and methanogenesis, and is performed by a complex

consortium of bacteria and archaea [4, 5]. The first three steps are predominantly fulfilled synergistically by fermentative bacteria from the phyla *Firmicutes*, *Bacteroidetes*, and *Proteobacteria*, while the last step is carried out by methanogenic archaea from the phylum *Euryarchaeota* [6]. However, the structure and performance of microbial communities in AD are strongly influenced by operating factors, such as feedstock, temperature, organic loading rate, and intermediate metabolites [5, 6]. Since the microbial communities in AD are extremely complex, the microbial compositions and interactions among microbes remain largely unclear [7].

Culture-independent technologies based on high-throughput sequencing enable the deep investigation of microbial compositions and functions. High-throughput 16S rRNA gene sequencing has been frequently used to analyze the taxonomic profile of AD microbial communities [8, 9]. Metagenomic approaches alone or coupled with metatranscriptomics, metaproteomics, and metabolomics are increasingly applied to decipher the gene functions, enzyme profiles, and metabolic processes of microbial communities in AD [10, 11]. However, most of these studies have focused only on a relatively small number of full-scale anaerobic digesters or with a small amount of

sequencing data [2, 3, 12, 13]. In this study, we collected different digestate samples from 56 full-scale biogas plants (BGPs), which were operated at different temperatures, fed with diverse feedstocks, and distributed widely in geographical regions, and constructed a microbial gene catalog of AD by in-depth metagenome sequencing.

Data description

To construct a Microbial Gene Catalog of AD (MGCA), 56 full-scale BGPs located all across China ranging from Northeast (45°27' N, 131°36' E) to Southwest (23°21' N, 131°36' E) (**Additional file 1: Fig S1**) were investigated. All plants were operated at ambient temperature (14-31.3°C at the time of sampling) or mesophilic (35-45°C) conditions, at pH 7.3-9.0, and with digester volume from 12 to 8000 m^3 (**Additional file 2: Table S1**).

Among these BGPs, 46 were in mono-digestion process, treating one of livestock manure (cattle, chicken or pig manure) alone, and the remaining 10 BGPs treat other animal manures alone or mixture of livestock manure and other substrates, such as

straw, vegetable or sewage water (**Additional file 2: Table S1**). According to their substrate types, these investigated BGPs were divided into four groups: MCA (13 cattle manure BGPs), MCH (6 chicken manure BGPs), MPI (27 pig manure BGPs), and OTH (10 BGPs with other substrates) (**Table 1**). There was a total number of 41 BGPs that adopt continuous stirred tank reactor (CSTR), and other BGPs adopt upflow solids reactor (USR), anaerobic baffled reactor (ABR), or black film digester (**Additional file 2: Table S1**). The most majority of these BGPs (53 BGPs) were in single-stage process and there were also 3 BGPs applied two-stage processes (**Additional file 2: Table S1**). Overall, these BGPs covers the typical and prevailing BGP types of AD and constitute a well representative collection.

Sample collection

Digestate samples were collected from fermentation tank or sampling valve. Before sampling, the reactor content was stirred and the sampling valve was opened for 5 min to flush the sampling valve and tubes. About 300 ml of digestate was sampled from each BGPs and transferred into 6 sterile, gastight tubes (50 ml) and frozen

immediately in a cooler with dry ice, and then transported to the laboratory. Frozen samples were stored at -80℃ before DNA extraction. In total, 59 digestate samples were collected, with 53 samples from 53 single-stage BGPs and 6 samples from each stage of 3 two-stage BGPs (JSP-03, SDP-01, and AHP-01) (**Additional file 2: Table S1**).

Table 1. Summary of the investigated full-scale biogas plants

Group	Feedstock type	Sample number[#]	BGP number	Reactor types[†]			Operate conditions[‡]	
				CSTR	USR	Others	Mesophilic	Ambient
MCA	Cattle manure	14	13	11	1	1	9	4
MCH	Chicken manure	7	6	4	1	1	5	1
MPI	Pig manure	28	27	21	3	3	8	19
OTH	Other substrates	10	10	5	3	2	6	4
Total		59	56	41	8	7	28	28

[#]: 53 samples from 53 single-stage BGPs and 6 samples from each stage of 3 two-stage BGPs (JSP-03, SDP-01, and AHP-01); [†]: Reactor types including CSTR, continuous stirred-tank reactor; USR, upflow anaerobic solid reactor; Others, including anaerobic baffled reactor (ABR), black film digester, and buried digester. [‡]: Operate conditions including mesophilic conditions and ambient temperature.

DNA extraction, library preparation and sequencing

Frozen digestate samples were taken out from -80℃ refrigerator and thawed at room

temperature. Genomic DNA was extracted in triplicate using the PowerSoil DNA Isolation Kit (cat. no. 12888-100; MoBio Laboratories Inc., USA) according to the manufacturer's protocol. To increase DNA yield, an extra physical cell disruption step of repeated freeze-thaw (four times of alternating between 65℃ and liquid nitrogen for 5 min) was employed prior to the standard protocol. The integrity of DNA extracts was checked on 0.7% (w/v) agarose gel with GelRed nucleic acid gel stain (cat. no. 41003; Biotium, USA). DNA samples showing obvious concentrated DNA bands > 15 kb in size were used for further analysis (**Additional file 3: Fig S2**). The quality and quantity of the extracted DNA were assessed using Nanodrop (Thermo Fisher Scientific, USA) and Qubit dsDNA HS assay kit (Thermo Fisher Scientific, USA). After DNA quality checks, the three replicates of high-quality DNA (band length > 15 kb, A260/280 1.8-2.0, dsDNA concentration > 20 ng/μL) of each sample were pooled for library construction.

Sequencing libraries were prepared for each sample using Illumina TruSeq DNA PCR-Free Library Preparation Kit (ref. 15037059; Illumina, USA) according to the manufacturer's instructions. In brief, a total of 1.5 μg metagenomic DNA was sheared

to 350 bp fragments using Covaris S220 (Covaris, USA), and the sheared DNA fragments were purified, blunt-end-repaired and size selected. Subsequently, a single ‘A’ nucleotide was added to the 3’ end of the blunt fragments, and then multiple indexing adapters were ligated to the A-tailed fragments by a complementary pairing single ‘T’ nucleotide on the 3’ end. All 59 prepared sequencing libraries were firstly checked for quality and quantity and then paired-end sequenced (2 x 150 bp) using Illumina Hiseq X10 platform (Illumina, USA) by Cloud Health Genomics Ltd (Shanghai, China). In total, 1,817 Gb of raw data were generated with 30.80 ± 3.77 Gb per sample (**Table 2**).

Metagenome assembly and construction of the gene catalog

The Illumina raw reads were cleaned by trimming the adapter sequences and low-quality regions using two in-house software clean_adapter and clean_lowqual [14] with default parameters, resulting in the clean reads with average error rate < 0.001 and read length ≥ 75 bp. In addition, unpaired reads were excluded from the clean reads. Then, we obtained a total of 1,064 Gb clean data, with an average of 18.03 ±

3.29 Gb per sample (**Table 2**). Firstly, the clean reads of each sample were assembled separately by Megahit v1.1.3 (Megahit, RRID:SCR_018551) [15] under paired-end mode, and the contigs with length < 1000 bp were filtered out. Then, the assembled

Table 2. Statistics of metagenome sequencing, assembly and non-redundant gene catalog (MGCA)

	Average value of each sample ± SD	Total [#]
Raw data (Gb)	30.80 ± 3.77	1,817
Clean data (Gb)	18.03 ± 3.29	1,064
Number of contigs [†]	243,272 ± 74,535	18,389,093
Assembled contigs length (Gb)	0.71 ± 0.19	49.38
Contig N50 value (bp)[*]	4,021 ± 758	3,267
Number of predicted genes[‡]	802,716 ± 217,466	56,953,553
Number of non-redundant genes	-	22,840,185
Percentage of full-length genes	-	56.45%
Average open reading frame length (bp)	-	790

[#]: Total, calculated from all data, including the data derived from independent assembly of each sample and co-assembly of all unmapped reads; [†]: contigs with length shorter than 1000 bp were filtered out; [*]: contig N50 value of co-assembled contigs (1,893 bp) were obviously shorter than that of independently assembled contigs of each sample (4,021 ± 758), and thus contig N50 value of all contigs (3,267 bp) were shorter than that of independent assembled contigs; [‡]: genes with length shorter than 102 bp were filtered out.

contigs were subjected to gene prediction using Prodigal v2.6.3 (Prodigal,

RRID:SCR_011936) [16] with parameter "-p meta", and the predicted genes with codon sequence length < 102 bp were filtered out according to a previous study [17]. As a result, we obtained an average contig number of 243,272 ± 74,535 (with contig N50 of 4,021 ± 758 bp) and gene number of 802,716 ± 217,466 for each sample (**Table 2**). To improve the assembly quality for less abundant species, clean reads of each sample were firstly mapped onto the assembled contigs of the sample with BWA-MEM (BWA, RRID:SCR_010910) [18], and then all the unmapped reads were pooled together for co-assembly. The software and parameters used for assembly and gene prediction of pooled unmapped reads were the same as above, and we obtained 4,035,874 contigs (with contig N50 of 1,893 bp) and 9,593,300 genes in total.

All the obtained genes were pooled (a total of 56,953,553 genes) and then clustered to construct an initial non-redundant gene catalog (22,844,545 genes) using CD-HIT-EST v4.6.6 (CD-HIT, RRID:SCR_007105) [19] with parameter "-c 0.95 –n 10 –G 0 –aS 0.9", adopts the criteria of identity ≥ 95% and alignment coverage ≥ 90% of the shorter genes (**Table 2**). The clean reads of each sample were mapped onto this initial gene catalog by BWA-MEM, and a total of 80.66% of qualified reads (with

alignment length ≥ 50 bp and identity > 95%) could be mapped. However, there were 4,360 genes having no qualified read mapped in any sample, which may be derived from wrong assembly or extreme low abundance, and they were removed from the gene catalog. At last, we got the final non-redundant MGCA of full-scale BGPs containing a total of 22,840,185 genes, with an average open reading frame length of 790 bp and a full-length gene percentage of 56.45% (**Table 2**).

The relative gene abundance of MGCA were calculated using the qualified reads [20, 21]. Briefly, for each sample, total number of reads mapped to all genes (TA) equal to the count of qualified reads, total number of reads mapped to one gene (TO) equal to the count of qualified reads mapped to the gene. At last, the normalized gene abundance (NGA) for each sample was calculated according the following formula: NGA = TO / (GL / 1,000) / (TA / 10,000,000); GL means the length of the gene. Rarefaction analysis was performed by counting the total number of detected genes in a given number of samples (≤ 59) after 100 random samplings with replacement. The rarefaction curve approached saturation with the increase of sample number (**Fig. 1a**), suggesting that our gene catalog covered the vast majority of microbial genes to be

found in the 56 full-scale BGPs sampled in this study. In addition, we compared the genes assigned to MCA (15,346,132 genes), MCH (9,707,833 genes), MPI (18,662,450 genes), and OTH (15,507,636 genes), and found that only a small proportion of genes (less than 12%) were unique in each of the four groups (**Fig. 1b**), which revealed that common microbial functions in AD were shared among different BGPs.

To assess to what extent that MGCA could represent the microbial genes in full-scale BGPs, a more Comprehensive Microbial Gene Catalog of AD (C-MGCA) of full-scale biogas plants was constructed. Except for the 59 metagenomes that generated in this study (1,817 Gb), other 39 metagenomes (580 Gb) derived from full-scale biogas plants, which located in Germany (22 samples), United Kingdom (12 samples), Spain (4 samples) and Sweden (1 sample), were downloaded from NCBI, ENA, or MGnify database (**Additional files 4: Table S2**). All data were integrated and processed using the same pipeline for MGCA, and 25,329,366 non-redundant genes were generated for C-MGCA. Based on pairwise alignments of the two gene catalogs at gene level using BLAT (BLAT, RRID:SCR_011919) [22], we found that

almost all genes in MGCA (99.99%) were shared by C-MGCA (with the criteria for shared genes that identity ≥ 95% and overlap ≥ 90% of the shorter genes), though C-MGCA only have 2,489,181 genes more than those of MGCA (**Additional files 5: Fig. S3**). In addition, six previously reported datasets derived from biogas plants [23-28] were processed using the same pipeline for MGCA and compared to the two gene catalogs. The results showed that only 52.3 ± 9.6% of genes in these datasets were shared by MGCA, while 99.5 ± 0.7% of genes were shared by C-MGCA (**Additional file 6: Table S3**), which were consistent with the fact that the data of the six datasets were used for constructing of C-MGCA. These results indicated that though MGCA contains a large proportion of genes in full-scale biogas plants, the gene coverage might be further improved by collecting more diversified samples, especially for those rare genes in specific types of AD process.

Taxonomic annotation of the gene catalog

Taxonomic annotation of genes in MGCA was performed using CARMA3 (CARMA, RRID:SCR_004999) [29] on the basis of DIAMOND v0.8.28.90 (DIAMOND,

RRID:SCR_016071) [30] alignment against the NCBI-NR database, according to a previously established method [20]. Of the 22,840,185 genes, 76.73% were taxonomically classified at the superkingdom level (**Fig. 2a**). Among these classified genes, 95.95% were assigned to Bacteria, and the remaining genes were assigned to Archaea (3.03%) and Eukaryota (1.02%). *Firmicutes* (23.04%), *Proteobacteria* (11.22%) and *Bacteroidetes* (9.93%) were the dominant phyla in the gene catalog (**Fig. 2a**), and *Euryarchaeota* (1.78%) was the predominant archaeal phylum, accounting for 76.69% of the archaeal genes. At lower taxonomic levels, only 9.62% and 0.51% of the genes were annotated to specific genera and species, respectively, highlighting the paucity of sequenced genomes of AD microbes in the public databases currently. In addition, genes classified to the methanogens in BGPs include those from *Methanosarcina* (0.16%), *Methanosaeta* (0.14%), *Methanoculleus* (0.14%), *Methanoregula* (0.13%), and *Methanobrevibacter* (0.10%) (**Fig. 2b**). To calculate the relative abundance of different taxonomic ranks (superkingdom, phylum, class, order, family, genus and species), the abundance of the respective genes belonging to each category according to the taxonomic assignments were added.

Functional annotation of the gene catalog

Functional annotation was performed by aligning all protein sequences in the gene catalog against the KEGG [31] database (release 79) using DIAMOND (v0.8.28.90), and taking the best hit with the criteria of E-value < 1e-5. As a result, 57.07% of genes were annotated with KEGG orthologous groups (KOs), with a total number of 13,527 KOs that were comparable to those of the gut microbial gene catalogs of pig and chicken [20, 32]. At the KEGG pathway level, more annotated genes were assigned to carbohydrate metabolism (19.89%), amino acid metabolism (14.61%), energy metabolism (10.52%), metabolism of cofactors and vitamins (10.19%) (**Fig. 3**). In particular, 163 KOs were identified in the methane metabolism pathway, including all KOs involved in all the three methanogenic pathways of acetoclastic, hydrogenotrophic and methylotrophic methanogenesis (**Additional file 7: Fig. S4**). In addition, to analyze the activities of carbohydrate hydrolysis, the genes encoding carbohydrate-active enzymes (CAZymes) were annotated by searching against the dbCAN [33] database (release 5.0) using hmmscan program (HMMER v3.0;

HMMER, RRID:SCR_005305) [34] and taking the best hit with the criteria of E-value < 1e-18 and coverage > 0.35. A total of 1,607,960 (7.04%) genes were annotated as CAZymes. Based on the functional assignments, relative abundance of CAZymes, KOs, and KEGG functional profiles were calculated by summing the abundance of the respective genes belonging to each category.

Characterization of core microbial communities in full-scale biogas plants

Identifying the core microbial populations across different full-scale biogas plants is important to understand the essential process in AD, and multiple studies have sought to define the core AD microbiome [9, 35, 36]. In the current study with the in-depth metagenomic sequencing of diverse full-scale BGPs, we found 400 genera and 6,816 KOs were shared by all the investigated samples (**Additional file 8: Fig. S5**), which accounted for about 98.76% and 99.39% of the total relative abundance of annotated genera and KOs, respectively.

However, the majority of the common microbes were in low abundance, and only a few abundant microbes could be considered as core members play important

roles in AD system. Thus, we defined core microbes by including genera that were both abundant and prevalent (most abundant top 30 bacterial genera and top 5 archaeal genera that were detected in all studied samples). As a result, only *Bacteroides* and *Clostridium* (**Fig. 4**), within the order of *Bacteroidales* and *Clostridiales*, were identified as core microbes. The result was consistent with previous study which detected *Bacteroidales* and *Clostridiales* from all 29 full-scale BGPs by 16S rRNA gene amplicon sequencing [8]. However, we should notice that *Bacteroides* and *Clostridium* were also the abundant genera in cattle, chicken and pig gut [20, 32, 37]. In addition, only two core genera were detected in all 59 samples, which were consistent with the phenomenon that it is hard to detect the core microbes from high number of investigated samples [9]. To compare the difference of the four groups, group-specific core microbes were analyzed, which were defined by the top genera that were detected in all samples of that group. At last, except for the genus *Bacteroides* and *Clostridium*, other 3 (*Corynebacterium*, *Treponema*, and *Methanosaeta*), 4 (*Acholeplasma*, *Pseudomonas*, *Sphaerochaeta*, and *Methanoculleus*), 1 (*Methanosarcina*), and 4 (*Prevotella*, *Ruminococcus*,

Sphaerochaeta, and *Treponema*) genera were identified as core microbes for MCA, MCH, MPI, and OTH, respectively (**Fig. 4**).

Microbial functional differentiation among BGPs with different feedstocks

Feedstock is an essential factor that drives microbial community variation in anaerobic digesters [38]. Principal coordinate analysis (PCoA) based on Bray-Curtis dissimilarity at species level were performed by the R package PHYLOSEQ, revealing that digestate samples were generally separated into three clusters (MCA, MCH and MPI), corresponding to the types of livestock manure (**Fig. 5a**). Microbial diversity (Shannon index) at the genus level also showed distinct differences among the groups, and the microbial diversity of MPI was significant (Wilcox rank sum test $P < 0.05$) higher than those of MCA and MCH (**Additional file 9: Fig. S6**).

To find the functional differences among the four groups, the relative abundance of genes involved in carbohydrate hydrolysis, protein hydrolysis, VFAs oxidation, and methanogenesis were compared. For genes involved in carbohydrate hydrolysis, we selected the CAZyme families involved in lignocellulose and starch hydrolysis and

categorized them in accordance with the CAZy database and previous studies [39-43] (**Additional file 10: Table S4**). The genes involved in protein hydrolysis (with Enzyme Commission number of EC 3.4.x.x) and methanogenesis were selected based on the KO annotation. The genes involved in acetate, propionate and butyrate oxidation pathways were selected according to the KEGG database and a previous study [44] (**Additional file 11: Table S5**).

As a result, for lignocellulose (cellulose, hemicelluloses, and lignin) degradation, the relative gene abundances were higher in MCA than those in MCH (significant higher for cellulose degradation; Wilcox rank sum test, $P < 0.05$), and significant (Wilcox rank sum test, $P < 0.05$) higher in MCA than those in MPI (**Fig. 5b**), which is consistent with the higher content of lignocellulose in cattle manure [45]. In contrast, genes involved in starch hydrolysis have higher relative abundance in MCH and MPI (**Fig. 5b**). Besides, the relative abundance of genes involved in the hydrolysis of proteins was much higher in MCH (**Fig. 5c**), which is associated with the relatively high protein content of chicken manure [45-47]. These results are consistent with the fact that the manures of the various animals are rich in these substances because their

feed is different from each other. VFAs such as acetate, propionate and butyrate, are intermediates in anaerobic digestion process, and the accumulation of VFAs may cause acidification and result in reduced performance of AD process. The results showed that MCH had the highest relative gene abundance involved in acetate oxidation, while MCA had significant (Wilcox rank sum test, $P < 0.05$) higher relative gene abundance involved in acetate, propionate and butyrate oxidation than those of MPI (**Fig. 5d**). In addition, as one of the most important step of biogas production, the genes involved in methanogenesis were compared, which revealed that MCH has the lowest relative gene abundance, and MCA was significant (Wilcox rank sum test, $p < 0.05$) higher than MCH and MPI (**Fig. 5e**). In summary, the feedstock components have great influence on the process of carbohydrate and protein hydrolysis, VFAs oxidation, and methanogenesis in BGPs.

In addition, various parameters in AD also have important effects on shaping microbial communities. Several process parameters (operation temperature; pH; hydraulic retention time, HRT, and reactor volume), physicochemical characteristics of feedstock (total nitrogen, TN; total carbon, TC; and total solid, TS) and

intermediate metabolites (total ammonia nitrogen, TAN; and VFAs) for all BGPs (**Additional file 2: Table S1**) from the groups MCA, MCH, MPI, and OTH were analyzed. Redundancy analysis (RDA) at the genus level revealed that operation temperature and TAN were primarily determinant parameters that influenced the microbial composition, and then followed by TS, acetate, total VFAs, TN, and pH (**Additional file 12: Fig. S7**). The result was agreed with a previous finding that TAN and digester temperature were identified as the main contributing factors to cluster formation [8].

Construction of metagenome-assembled genomes

To reconstruct the metagenome-assembled genomes (MAGs), all 59 digestate samples were included. Metagenome binning was applied to single-sample assemblies, which were performed in "Metagenome assembly" step, and the contigs with length < 1000 bp were filtered out. BBmap v38.50 (BBmap, RRID:SCR_016965) [48] was used to map reads of each sample back to the filtered assembly with default parameters. Samtools v1.9 (Samtools, RRID:SCR_002105) [49] was used to convert SAM files to

BAM format and sort the resulting BAM files. Genomes were independently recovered from each sample using MetaBAT2 v2.12.1 (MetaBAT, RRID:SCR_019134) [50], with the option --minContig 2000, and a total of 11,781 MAGs were generated from all 59 samples. The completeness (Cp) and contamination (Ct) of all MAGs were estimated using “Lineage_wf” workflow of CheckM v1.0.7 (CheckM, RRID:SCR_016646) [51] with options lineage_wf -t 20 -x fa. After filtering for Cp $\geq$ 80% and Ct $\leq$ 10%, 3,601 MAGs were left for further de-replication.

MAGs de-replication was performed using Mash v2.2 (Mash, RRID:SCR_019135) [52] on the entire genome sequences with very permissive parameters dist -d 0.05 [53], and MAGs were clustered into different groups. To determine the representative MAGs of each group, a more precise analysis was performed applying the genome-wide Average Nucleotide Identity (ANI) [54]. MAGs were considered as belonging to the same species when they showed ANI value higher than 95% and genome coverage higher than 50% for both strains, and the MAG with the highest CC3 value (CC3 = Cp – Ct * 3) was selected as the

representative one [53]. As a result, a total of 2,426 representative MAGs were obtained, including 1,205 MAGs (49.7%) with completeness ≥ 90% and contamination ≤ 5% (**Additional file 13: Table S6**).

To estimate the degree of novelty of our study, we performed a comparison with 1401 MAGs (Cp ≥ 70% and Ct < 10%) recovered from a previous study [53], which using 134 publicly available metagenomes derived from various biogas reactors. However, the results showed only 108 MAGs in our study were the same species to those in 1401 MAGs, which were consistent with the fact that most metagenomes were derived from lab-scale biogas reactors and batch tests in cited study, while all metagenomes were derived from full-scale digesters in our study. Taxonomic annotation of MAGs was performed using the GTDB-Tk v1.3.0 (GTDB-Tk, RRID:SCR_019136) [55], and 96.08% and 3.92 % of MAGs were assigned to Bacteria and Archaea, respectively. In addition, *Firmicutes* (38.25%), *Bacteroidetes* (21.89%), and *Proteobacteria* (5.03%) were the dominant phyla in these MAGs, which was consistent with the microbial compositions at phylum level derived from gene catalog. In summary, our study provides a huge number of MAGs for full-scale

biogas plants.

Conclusions

Here, we present a microbial gene catalog of anaerobic digestion (AD), by using in-depth sequencing of the digestate samples from 56 full-scale biogas plants (BGPs) treating diverse feedstocks, and provide over 22.8 million taxonomically and functionally annotated genes. Our results confirmed the existence of core microbiome in AD, and showed that the type of feedstock (cattle, chicken and pig manure) has a great influence on carbohydrate hydrolysis, VFAs oxidation, and methanogenesis. Additionally, we also provided 2,426 MAGs derived from full-scale biogas plants. Compared to previously published microbial gene catalogs of different ecosystems such as soil, ocean, animal gut and rumen [20, 32, 56-59], biogas plants are man-made extremely anaerobic ecosystems where AD is performed by a complex consortium of anaerobic microbes. Hence, our gene catalog will not only serve as a useful reference database for quick analyses of AD microbiome data, but also provide a huge number of microbial gene resources for the study and utilization of anaerobic

microbiota.

Data Availability

All raw sequencing data generated during the current study have been deposited at DDBJ/ENA/GenBank under project accession PRJNA533495. For detail, SRR8925713 ~ SRR8925730, SRR8925732 ~ SRR8925742, SRR8925747 ~ SRR8925748, SRR8925751 ~ SRR8925758, SRR8925797 ~ SRR8925806, SRR8925817 ~ SRR8925824, and SRR8925826 ~ SRR8925827 for metagenome sequencing data of 59 digestate samples. Other supporting data, including the files of gene sequences, taxonomic and functional annotations, and the abundance profile tables of the two gene catalogs (MGCA and C-MGCA), and metagenome-assembled genomes (MAGs) generated in this study are available in a the *GigaScience* GigaDB repository [60].

Declarations

List of abbreviations

ABR: anaerobic baffled reactor; AD: anaerobic digestion; BGP: biogas plant; CAZyme: carbohydrate-active enzyme; C-MGCA: comprehensive microbial gene catalog of AD; CSTR: continuous stirred tank reactor; Gb: gigabase; HRT: hydraulic retention time; KO: KEGG orthologous group; MAG: metagenome-assembled genome; MCA: cattle manure biogas plants; MCH: chicken manure biogas plants; MGCA: microbial gene catalog of AD; MPI: pig manure biogas plants; OTH: biogas plants with other feedstocks; PCoA: principal coordinate analysis; TAN: total ammonia nitrogen; TC: total carbon; TN: total nitrogen; TS: total solid; USR: upflow solids reactor; VFA: volatile fatty acid.

Consent for publication

Not applicable.

Competing interests

The authors declare that they have no competing interests.

Funding

This project was supported by grants from Shenzhen science and technology program (JCYJ20190814163805604), Agricultural Science and Technology Innovation Program (ASTIP), Chinese Academy of Agricultural Sciences (CAAS-ASTIP-2016-BIOMA), the Agricultural Science and Technology Innovation Program && The Elite Young Scientists Program of CAAS, Fundamental Research Funds for Central Non-profit Scientific Institution (No. Y2017JC01), Science and Technology Program of Sichuan Province, China (2017JY0242), the Agricultural Science and Technology Innovation Program Cooperation and Innovation Mission (CAAS-XTCX2016), the Fund of Key Laboratory of Shenzhen (ZDSYS20141118170111640), the Fundamental Research Funds for Central Non-profit Scientific Institution, China (1610012016023) and the Infrastructure and Facility Development Program of Sichuan Province (2019JDPT0012). The sponsors had no role in design or conduct of the study; the collection, management, analysis, or interpretation of the data; the preparation, review, or approval of the manuscript; or the decision to submit the manuscript for publication.

Authors’ contributions

SM, YH, HF, and QL collected the samples, and FJ, YZ, LY, and SL extracted the DNA and constructed the Illumina sequencing libraries. SM, FJ, YH, YZ, SW, BL, and HW analyzed the data. HL and YR provide helpful suggestions. SM, FJ, YH, YZ, and SW wrote the raw manuscript. WF, YD, and LC conceived the study, designed the experiments, and revised the manuscript. All authors read and approved the final manuscript.

Acknowledgements

We thank Jing He, Yunfei Zhang, Yanlai Liu, Xia Li, Bo Tu, Shouchao Lai, Nengmin Zhu, Lirong Dai, Lu Yang, Yinggang Zhang from Biogas Institute of Ministry of Agricultural and Rural Affairs for collecting samples. We also express our thanks to Jianjun Hu, Ling Qiu, Zuojun Liu, Liumeng Chen, Xiaomei Ye for their assistance with sample collection.

References

1. Tyagi VK and Lo SL. Sludge: A waste or renewable source for energy and resources recovery? Renew Sust Energ Rev. 2013;25:708-28.

2. Stolze Y, Bremges A, Rumming M, Henke C, Maus I, Pühler A, et al. Identification and genome reconstruction of abundant distinct taxa in microbiomes from one thermophilic and three mesophilic production-scale biogas plants. Biotechnol Biofuels. 2016;9:156.

3. Luo G, Fotidis IA, and Angelidaki I. Comparative analysis of taxonomic, functional, and metabolic patterns of microbiomes from 14 full-scale biogas reactors by metagenomic sequencing and radioisotopic analysis. Biotechnol Biofuels. 2016;9:51.

4. Angenent LT, Karim K, Al-Dahhan MH, Wrenn BA, and Domiguez-Espinosa R. Production of bioenergy and biochemicals from industrial and agricultural wastewater. Trends Biotechnol. 2004;22:477-85.

5. Hassa J, Maus I, Off S, Pühler A, Scherer P, Klocke M, et al. Metagenome, metatranscriptome, and metaproteome approaches unraveled compositions and functional relationships of microbial communities residing in biogas plants.

Appl Microbiol Biotechnol. 2018;102:5045-63.

6. Schnürer A. Biogas production: microbiology and technology. Adv Biochem Eng Biotechnol. 2016;156:195-234.

7. Narihiro T, Nobu MK, Kim NK, Kamagata Y, and Liu WT. The nexus of syntrophy-associated microbiota in anaerobic digestion revealed by long-term enrichment and community survey. Environ Microbiol. 2015;17:1707-20.

8. De Vrieze J, Saunders AM, He Y, Fang J, Nielsen PH, Verstraete W, et al. Ammonia and temperature determine potential clustering in the anaerobic digestion microbiome. Water Res. 2015;75:312-23.

9. Mei R, Nobu MK, Narihiro T, Kuroda K, Munoz Sierra J, Wu Z, et al. Operation-driven heterogeneity and overlooked feed-associated populations in global anaerobic digester microbiome. Water Res. 2017;124:77-84.

10. Jia Y, Ng SK, Lu H, Cai M, and Lee PKH. Genome-centric metatranscriptomes and ecological roles of the active microbial populations during cellulosic biomass anaerobic digestion. Biotechnol Biofuels. 2018;11:117.

11. Treu L, Kougias PG, Campanaro S, Bassani I, and Angelidaki I. Deeper insight into the structure of the anaerobic digestion microbial community; the biogas microbiome database is expanded with 157 new genomes. Bioresour Technol. 2016;216:260-6.

12. Campanaro S, Treu L, Kougias PG, Luo G, and Angelidaki I. Metagenomic binning reveals the functional roles of core abundant microorganisms in twelve full-scale biogas plants. Water Res. 2018;140:123-34.

13. Campanaro S, Treu L, Kougias PG, De Francisci D, Valle G, and Angelidaki I. Metagenomic analysis and functional characterization of the biogas microbiome using high throughput shotgun sequencing and a novel binning strategy. Biotechnol Biofuels. 2016;9:26.

14. Clean_adapter and clean_lowqul github repository. https://github.com/fanagislab/DBG_assembly/tree/master/clean_illumina

15. Li DH, Luo RB, Liu CM, Leung CM, Ting HF, Sadakane K, et al. MEGAHIT v1.0: A fast and scalable metagenome assembler driven by advanced methodologies and community practices. Methods. 2016;102:3-11.

16. Hyatt D, LoCascio PF, Hauser LJ, and Uberbacher EC. Gene and translation initiation site prediction in metagenomic sequences. Bioinformatics. 2012;28:2223-30.

17. Qin J, Li R, Raes J, Arumugam M, Burgdorf KS, Manichanh C, et al. A human gut microbial gene catalogue established by metagenomic sequencing. Nature. 2010;464:59-65.

18. Li H and Durbin R. Fast and accurate short read alignment with Burrows-Wheeler transform. Bioinformatics. 2009;25:1754-60.

19. Fu LM, Niu BF, Zhu ZW, Wu ST, and Li WZ. CD-HIT: accelerated for clustering the next-generation sequencing data. Bioinformatics. 2012;28:3150-52.

20. Huang P, Zhang Y, Xiao KP, Jiang F, Wang HC, Tang DZ, et al. The chicken gut metagenome and the modulatory effects of plant-derived benzylisoquinoline alkaloids. Microbiome. 2018;6:211.

21. Qin JJ, Li YR, Cai ZM, Li SH, Zhu JF, Zhang F, et al. A metagenome-wide association study of gut microbiota in type 2 diabetes. Nature. 2012;490:55-60.

22. Kent WJ. BLAT--the BLAST-like alignment tool. Genome Res. 2002;12:656-64.

23. Maus I, Koeck DE, Cibis KG, Hahnke S, Kim YS, Langer T, et al. Unraveling the microbiome of a thermophilic biogas plant by metagenome and metatranscriptome analysis complemented by characterization of bacterial and archaeal isolates. Biotechnol Biofuels. 2016;9:171.

24. Ortseifen V, Stolze Y, Maus I, Sczyrba A, Bremges A, Albaum SP, et al. An integrated metagenome and -proteome analysis of the microbial community residing in a biogas production plant. J Biotechnol. 2016;231:268-79.

25. Gullert S, Fischer MA, Turaev D, Noebauer B, Ilmberger N, Wemheuer B, et al. Deep metagenome and metatranscriptome analyses of microbial communities affiliated with an industrial biogas fermenter, a cow rumen, and elephant feces reveal major differences in carbohydrate hydrolysis strategies. Biotechnol Biofuels. 2016;9:121.

26. Bremges A, Maus I, Belmann P, Eikmeyer F, Winkler A, Albersmeier A, et al. Deeply sequenced metagenome and metatranscriptome of a biogas-producing

microbial community from an agricultural production-scale biogas plant. Gigascience. 2015;4:33.

27. Sun L, Muller B, Westerholm M, and Schnürer A. Syntrophic acetate oxidation in industrial CSTR biogas digesters. J Biotechnol. 2014;171:39-44.

28. Ruiz-Sanchez J, Campanaro S, Guivernau M, Fernandez B, and Prenafeta-Boldu FX. Effect of ammonia on the active microbiome and metagenome from stable full-scale digesters. Bioresour Technol. 2018;250:513-22.

29. Gerlach W and Stoye J. Taxonomic classification of metagenomic shotgun sequences with CARMA3. Nucleic Acids Res. 2011;39:e91.

30. Buchfink B, Xie C, and Huson DH. Fast and sensitive protein alignment using DIAMOND. Nat Methods. 2015;12:59-60.

31. Kanehisa M, Goto S, Kawashima S, Okuno Y, and Hattori M. The KEGG resource for deciphering the genome. Nucleic Acids Res. 2004;32:D277-D80.

32. Xiao L, Estelle J, Kiilerich P, Ramayo-Caldas Y, Xia ZK, Feng Q, et al. A reference gene catalogue of the pig gut microbiome. Nat Microbiol. 2016;1:16161.

33. Yin YB, Mao XZ, Yang JC, Chen X, Mao FL, and Xu Y. dbCAN: a web resource for automated carbohydrate-active enzyme annotation. Nucleic Acids Res. 2012;40:W445-W51.

34. Eddy SR. Accelerated Profile HMM Searches. Plos Comput Biol. 2011;7:e1002195.

35. Mei R, Narihiro T, Nobu MK, Kuroda K, and Liu WT. Evaluating digestion efficiency in full-scale anaerobic digesters by identifying active microbial populations through the lens of microbial activity. Scientific Reports. 2016;6:34090.

36. Calusinska M, Goux X, Fossepre M, Muller EEL, Wilmes P, and Delfosse P. A year of monitoring 20 mesophilic full-scale bioreactors reveals the existence of stable but different core microbiomes in bio-waste and wastewater anaerobic digestion systems. Biotechnol Biofuels. 2018;11:196.

37. Wirth R, Kadar G, Kakuk B, Maroti G, Bagi Z, Szilagyi A, et al. The Planktonic Core Microbiome and Core Functions in the Cattle Rumen by Next Generation Sequencing. Front Microbiol. 2018;9:2285.

38. Zhang W, Werner JJ, Agler MT, and Angenent LT. Substrate type drives variation in reactor microbiomes of anaerobic digesters. Bioresour Technol. 2014;151:397-401.

39. Artzi L, Bayer EA, and Moraïs S. Cellulosomes: bacterial nanomachines for dismantling plant polysaccharides. Nat Rev Microbiol. 2016;15:83-95.

40. Gharechahi J and Salekdeh GH. A metagenomic analysis of the camel rumen's microbiome identifies the major microbes responsible for lignocellulose degradation and fermentation. Biotechnol Biofuels. 2018;11:216.

41. Kougias PG, Campanaro S, Treu L, Tsapekos P, Armani A, and Angelidaki I. Spatial distribution and diverse metabolic functions of lignocellulose-degrading uncultured bacteria as revealed by genome-centric metagenomics. Appl Environ Microbiol. 2018;84:e01244-18.

42. Liu N, Li H, Chevrette MG, Zhang L, Cao L, Zhou H, et al. Functional metagenomics reveals abundant polysaccharide-degrading gene clusters and cellobiose utilization pathways within gut microbiota of a wood-feeding higher termite. ISME J. 2019;13:104-17.

43. Zhu N, Yang J, Ji L, Liu J, Yang Y, and Yuan H. Metagenomic and metaproteomic analyses of a corn stover-adapted microbial consortium EMSD5 reveal its taxonomic and enzymatic basis for degrading lignocellulose. Biotechnol Biofuels. 2016;9:243.

44. Mosbaek F, Kjeldal H, Mulat DG, Albertsen M, Ward AJ, Feilberg A, et al. Identification of syntrophic acetate-oxidizing bacteria in anaerobic digesters by combined protein-based stable isotope probing and metagenomics. ISME J. 2016;10:2405-18.

45. Wang M, Li W, Li P, Yan S, and Zhang Y. An alternative parameter to characterize biogas materials: Available carbon-nitrogen ratio. Waste Manag. 2017;62:76-83.

46. Sheu SY, Liu LP, and Chen WM. *Novosphingobium bradum* sp. nov., isolated from a spring. Int J Syst Evol Microbiol. 2016;66:5083-90.

47. Zakharyuk A, Kozyreva L, Ariskina E, Troshina O, Kopitsyn D, and Shcherbakova V. *Alkaliphilus namsaraevii* sp. nov., an alkaliphilic iron- and sulfur-reducing bacterium isolated from a steppe soda lake. Int J Syst Evol

Microbiol. 2017;67:1990-95.

48. BBMap on sourceforge. https://sourceforge.net/projects/bbmap/

49. Li H, Handsaker B, Wysoker A, Fennell T, Ruan J, Homer N, et al. The Sequence Alignment/Map format and SAMtools. Bioinformatics. 2009;25:2078-9.

50. Kang DD, Li F, Kirton E, Thomas A, Egan R, An H, et al. MetaBAT 2: an adaptive binning algorithm for robust and efficient genome reconstruction from metagenome assemblies. PeerJ. 2019;7:e7359.

51. Parks DH, Imelfort M, Skennerton CT, Hugenholtz P, and Tyson GW. CheckM: assessing the quality of microbial genomes recovered from isolates, single cells, and metagenomes. Genome Res. 2015;25:1043-55.

52. Ondov BD, Treangen TJ, Melsted P, Mallonee AB, Bergman NH, Koren S, et al. Mash: fast genome and metagenome distance estimation using MinHash. Genome Biol. 2016;17:132.

53. Campanaro S, Treu L, Rodriguez RL, Kovalovszki A, Ziels RM, Maus I, et al. New insights from the biogas microbiome by comprehensive genome-resolved

metagenomics of nearly 1600 species originating from multiple anaerobic digesters. Biotechnol Biofuels. 2020;13:25.

54. Varghese NJ, Mukherjee S, Ivanova N, Konstantinidis KT, Mavrommatis K, Kyrpides NC, et al. Microbial species delineation using whole genome sequences, Nucleic Acids Res. 2015;43:6761-71.

55. Chaumeil PA, Mussig AJ, Hugenholtz P, and Parks DH. GTDB-Tk: a toolkit to classify genomes with the Genome Taxonomy Database. Bioinformatics. 2020;36:1925-1927.

56. Li J, Zhong H, Ramayo-Caldas Y, Terrapon N, Lombard V, Potocki-Veronese G, et al. A catalog of microbial genes from the bovine rumen unveils a specialized and diverse biomass-degrading environment. Gigascience. 2020;9:1-15.

57. Bahram M, Hildebrand F, Forslund SK, Anderson JL, Soudzilovskaia NA, Bodegom PM, et al. Structure and function of the global topsoil microbiome. Nature. 2018;560:233-37.

58. Sunagawa S, Coelho LP, Chaffron S, Kultima JR, Labadie K, Salazar G, et al.

Ocean plankton. Structure and function of the global ocean microbiome. Science. 2015;348:1261359.

59. Li J, Jia H, Cai X, Zhong H, Feng Q, Sunagawa S, et al. An integrated catalog of reference genes in the human gut microbiome. Nat Biotechnol. 2014;32:834-41.

60. Ma SC; Jiang F; Huang Y; Zhang Y; Wang S; Fan H; Liu B; Li Q; Yin LJ; Wang HC; Liu HW; Ren YW; Li SQ; Cheng L; Fan W; Deng Y (2020): Supporting data for "A microbial gene catalog of anaerobic digestion from full-scale biogas plants" GigaScience Database. http://dx.doi.org/10.5524/100842

Figure legends and supplementary files

Fig. 1 The constructed microbial gene catalog of anaerobic digestion (MGCA). **a** Rarefaction curve of detected genes from the whole set of 59 digestate samples. The curve approaches saturation as sample number increases. The gene number of a given number of samples was calculated after 100 random samplings with replacement and

plotted with a box plot. Boxplots show the median ± interquartile range (IQR) and 1.5 IQR ranges (whiskers), with outliers denoted by circles. **b** Venn diagram of shared genes among four groups of non-redundant genes from MCA, MCH, MPI and OTH. Only a small proportion of genes were unique for each group. MCA, cattle manure BGPs; MCH, chicken manure BGPs; MPI, pig manure BGPs; OTH, BGPs with other substrates.

Fig. 2 Taxonomic annotation of the microbial gene catalog of anaerobic digestion (MGCA). **a** Taxonomic annotation of the gene catalog at the superkingdom and phylum levels. A total of 73.63% and 2.32% of genes in the gene catalog were assigned to Bacteria and Archaea, respectively. **b** Percentage of genes assigned to the top 10 methanogenic archaea at genus level.

Fig. 3 KEGG functional profile of the microbial gene catalog of anaerobic digestion (MGCA). Genes without functional annotations were excluded.

Fig. 4 Distributions of feedstock-associated core genera among four group of MCA, MCH, MPI, and OTH. The area of each circle represents the median value of relative abundance of the corresponding genus in each group, and the non-core genera were not presented. “Core microbes” were defined as the genera most abundant top 30 bacterial genera and top 5 archaeal genera that were detected in all studied samples. MCA, cattle manure BGPs; MCH, chicken manure BGPs; MPI, pig manure BGPs; OTH, BGPs with other substrates.

Fig. 5 Comparisons of taxonomic and functional profiles among different biogas plants (BGPs). **a** Principal coordinate analysis (PCoA) based on Bray-Curtis dissimilarity at the species level. The digestate samples were separated into three clusters (MCA, MCH and MPI). MCA, cattle manure BGPs; MCH, chicken manure BGPs; MPI, pig manure BGPs; OTH, BGPs with other substrates. **b** Relative abundance of genes involved in the hydrolysis of starch, oligosaccharide, polysaccharide, and lignocellulose (lignin, hemicellulose, and cellulose) hydrolysis. **c** Relative abundance of genes involved in protein hydrolysis. **d** Relative abundance of

genes involved in acetate, propionate, and butyrate oxidation. **e** Relative abundance of genes involved in methanogenesis. Boxplots show the median ± interquartile range (IQR) and 1.5 IQR ranges (whiskers), with outliers denoted by circles. Wilcox rank sum test among different groups were performed, and asterisks denote significant difference ($P < 0.05$) between the two groups.

Additional file 1: Fig. S1 Geographic distribution of 56 full-scale biogas plants (BGPs) from which the digestate samples were collected. The sampling BGPs ranged in location from the Northeast (45°27' N , 131°36' E) to the Southwest (23°21' N, 103°20' E) China, including cattle manure BGPs (MCA), chicken manure BGPs (MCH), pig manure BGPs (MPI), and BGPs with other feedstocks (OTH). (PDF 5500K)

Additional file 2: Table S1 Background information of the investigated 56 full-scale biogas plants (BGPs). (XLSX 26K)

Additional file 3: Fig. S2 Electrophoresis graph of DNA samples. (PDF 696K)

Additional file 4: Table S2 Information of the sequencing data downloaded from public database. (XLSX 56K)

Additional file 5: Fig. S3 Rarefaction analysis of gene catalogs MGCA and C-MGCA. The gene number of a given number of samples was calculated after 100 random samplings with replacement. (PDF 852K)

Additional file 6: Table S3 Overlap of genes between gene sets of public metagenome sequencing data and MGCA and C-MGCA. (XLSX 35K)

Additional file 7: Fig. S4 The KEGG methane metabolism pathway. The enzymes present in 100% of digestate samples (59 samples) were highlighted in red, the enzymes present in more than 90% of digestate samples were highlighted in light blue, and other enzymes annotated in the gene catalog were shown in green. The enzymes

analyzed based on the KO annotation. (PDF 962K)

Additional file 8: Fig. S5 The number of shared genera and KOs among biogas plants (BGPs) at different frequency thresholds. (PDF 873K)

Additional file 9: Fig. S6 Shannon index of MCA, MCH and MPI at the genus level. MCA, cattle manure biogas plants (BGPs); MCH, chicken manure BGPs; MPI, pig manure BGPs. Boxplots show median ± interquartile range (IQR) and 1.5 IQR ranges (whiskers), with outliers denoted by circles. Wilcox rank sum test among different groups were performed, and asterisks denote significant difference ($P < 0.05$) between the two groups. (PDF 867K)

Additional file 10: Table S4 Categories of CAZyme families. (XLSX 13K)

Additional file 11: Table S5 Genes selected for the analysis of the acetate, propionate and butyrate oxidation pathways. (XLSX 13K)

Additional file 12: Fig. S7 Redundancy analysis (RDA) of microbial communities and operational parameters. Red arrows indicate the influence of process parameters (operation temperature; pH; hydraulic retention time, HRT, and reactor volume), physicochemical characteristics of feedstock (total nitrogen, TN; total carbon, TC; and total solid, TS) and intermediate metabolites (total ammonia nitrogen, TAN; and VFAs) on microbial communities. Colored dots indicate samples of different groups of BGPs. (PDF 136K)

Additional file 13: Table S6 Statistics and taxonomic annotation of metagenome-assembled genomes (MAGs). (XLSX 136K)

Figure 1

Click here to access/download;Figure;Figure 1.pdf

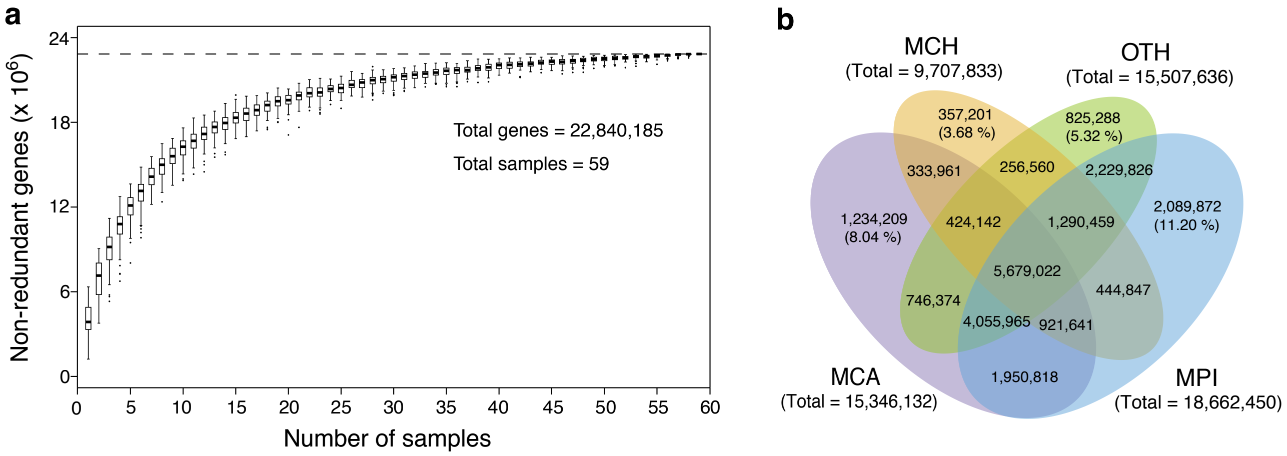

Figure 2

Click here to access/download;Figure;Figure 2.pdf

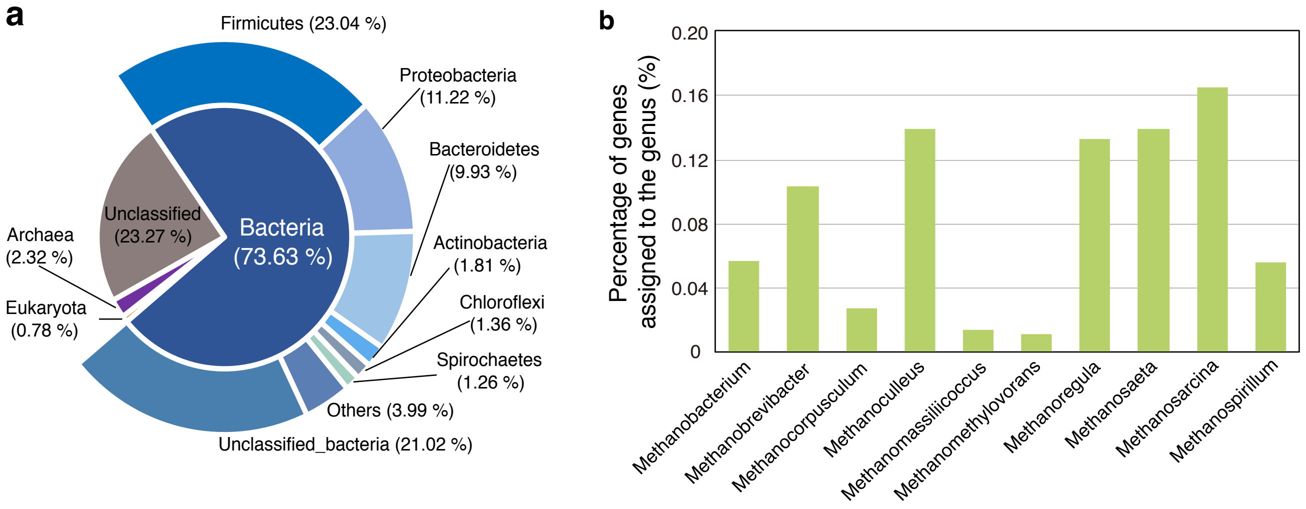

Figure 3

Click here to access/download;Figure;Figure 3.pdf

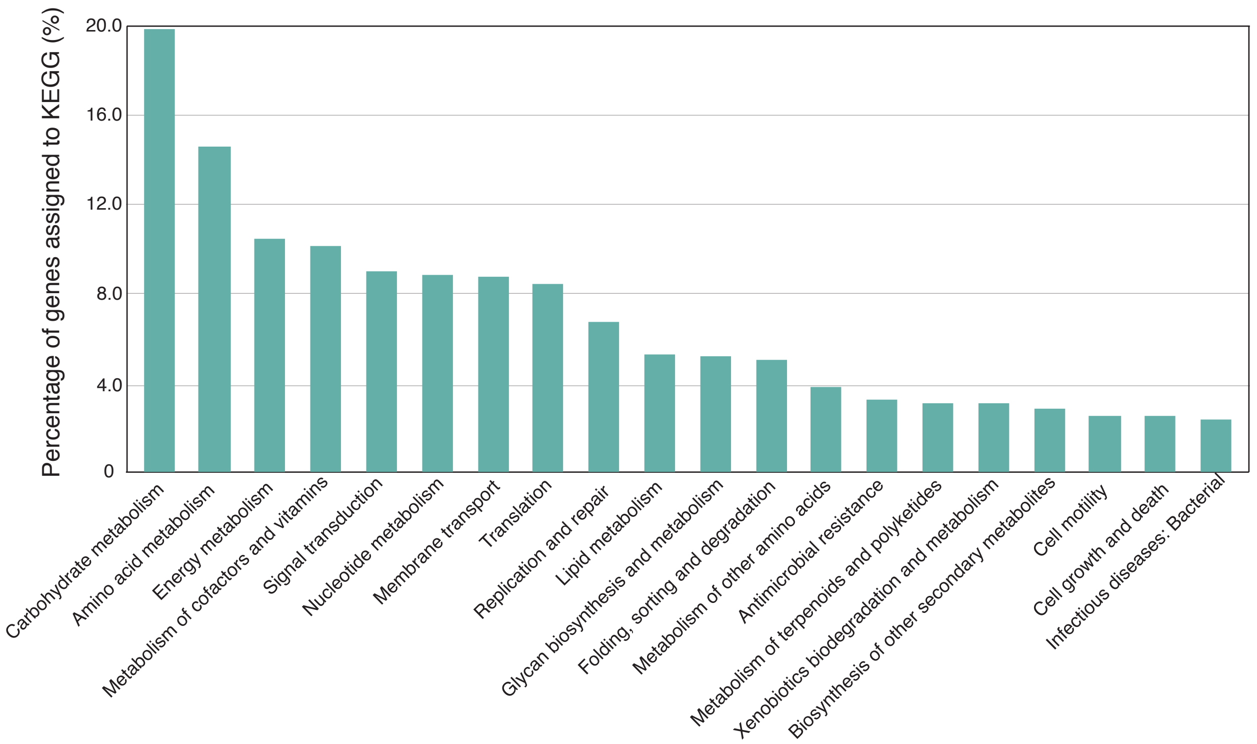

Figure 4

Click here to access/download;Figure;Figure 4.pdf

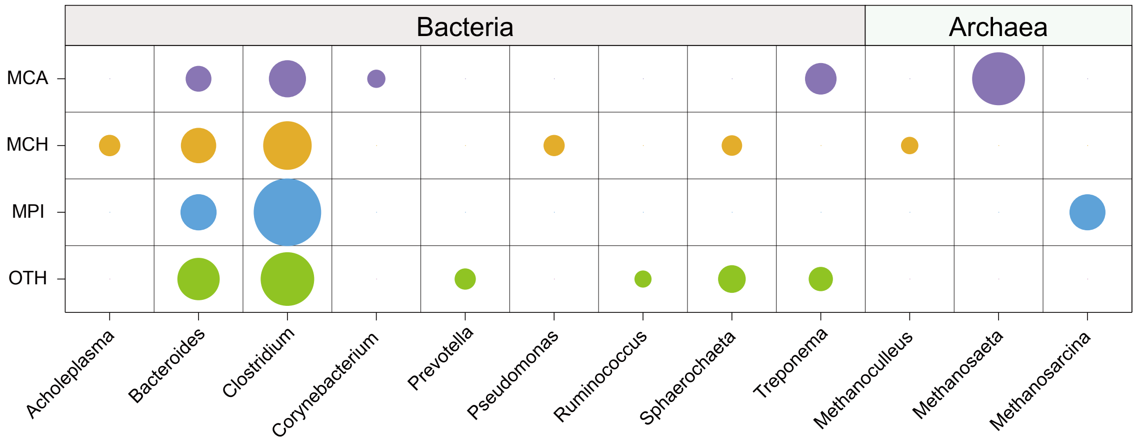

Figure 5

Click here to access/download;Figure;Figure 5.pdf

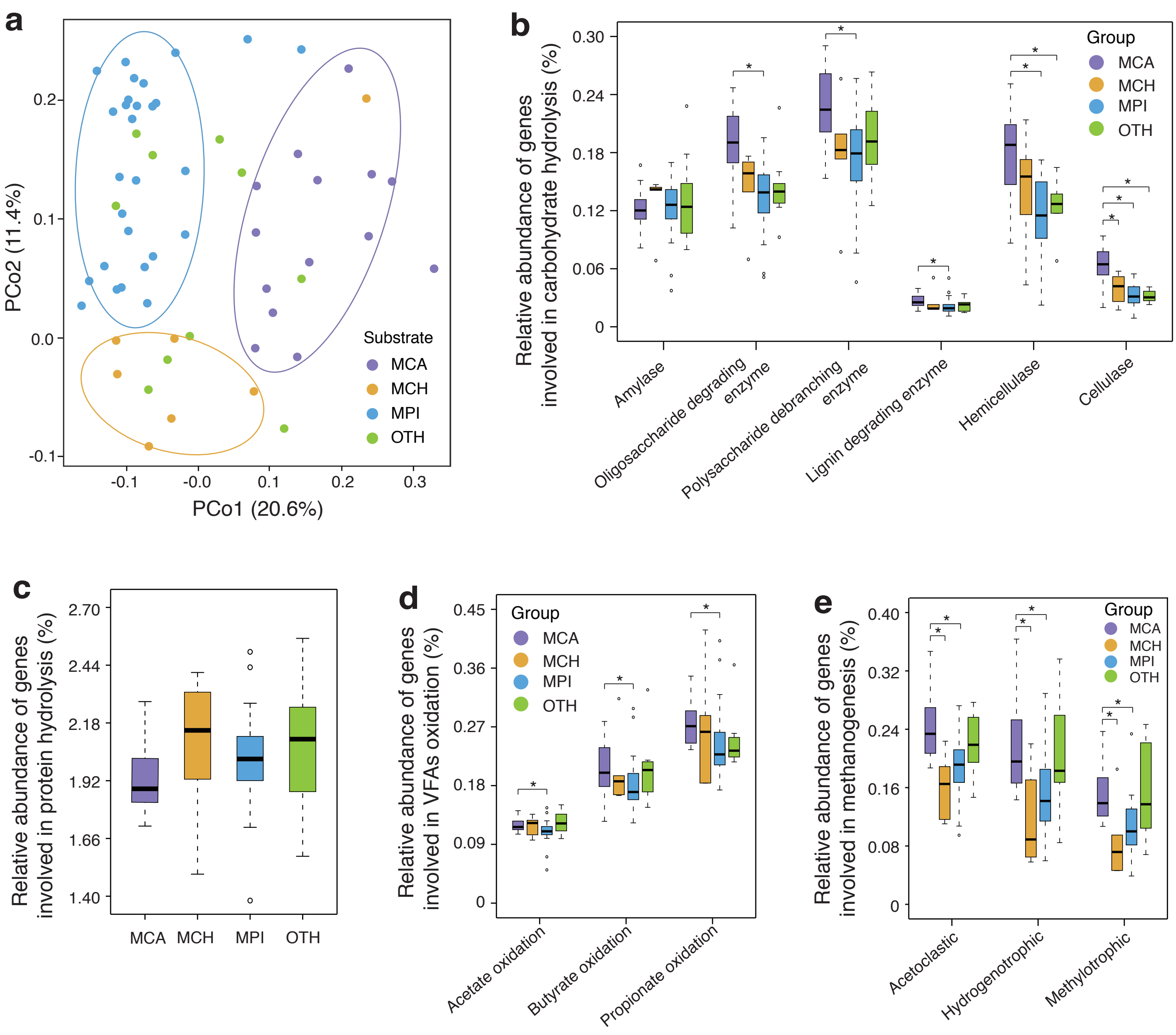

Response to reviewer comments

Click here to access/download
Supplementary Material
ResponsetoReviewerComments.docx

Additional file 1

Click here to access/download
Supplementary Material
Additional file 1-Fig. S1.pdf

Additional file 2

Click here to access/download

Supplementary Material

Additional file 2-Table S1.xlsx

Additional file 3

Click here to access/download
Supplementary Material
Additional file 3-Fig S2.pdf

Click here to access/download
Supplementary Material
Additional file 4-Table S2.xlsx

Additional file 5

Click here to access/download
Supplementary Material
Additional file 5-Fig. S3.pdf

Click here to access/download
Supplementary Material
Additional file 6-Table S3.xlsx

Additional file 7

Click here to access/download
Supplementary Material
Additional file 7-Fig. S4.pdf

Additional file 8

Click here to access/download
Supplementary Material
Additional file 8-Fig. S5.pdf

Additional file 9

Click here to access/download
Supplementary Material
Additional file 9-Fig. S6.pdf

Additional file 10

Click here to access/download
Supplementary Material
Additional file 10-Table S4.xlsx

Additional file 11

Click here to access/download
Supplementary Material
Additional file 11-Table S5.xlsx

Additional file 12

Click here to access/download
Supplementary Material
Additional file 12-Fig. S7.pdf

Additional file 13

Click here to access/download
Supplementary Material
Additional file 13-Table S6.xlsx
