# Supplementary figures and images for: A microbial gene catalog of anaerobic digestion from full-scale biogas plants
Source: Gigascience. 2021 Jan 27;10(1):giaa164. doi: 10.1093/gigascience/giaa164 (PMC7842101; doi:10.1093/gigascience/giaa164)

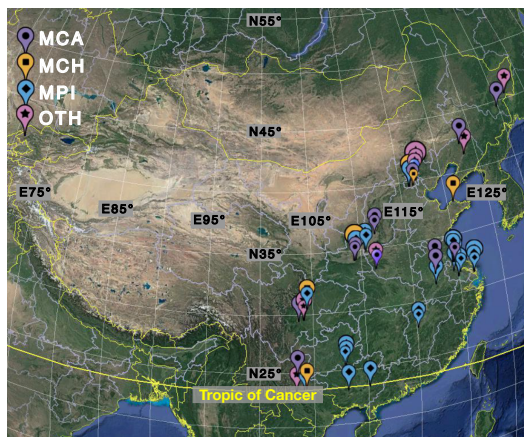

Supplement: giaa164_Supplemental_Files [file giaa164_supplemental_files.zip › Additional file 1-Fig. S1.pdf]

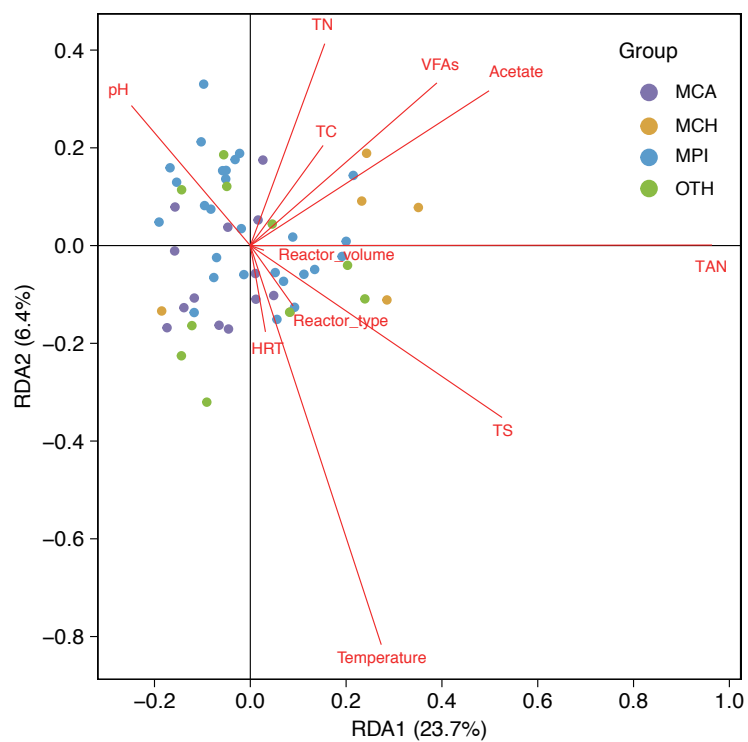

Supplement: giaa164_Supplemental_Files [file giaa164_supplemental_files.zip › Additional file 12-Fig. S7.pdf]

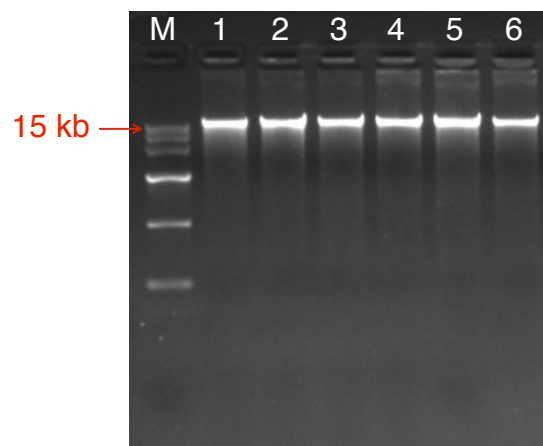

Supplement: giaa164_Supplemental_Files [file giaa164_supplemental_files.zip › Additional file 3-Fig S2.pdf]

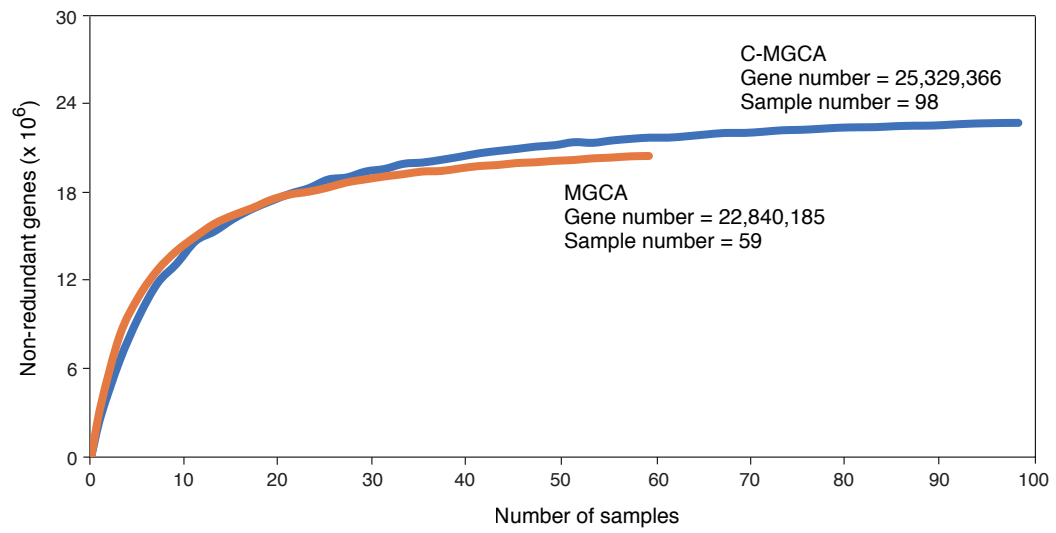

Supplement: giaa164_Supplemental_Files [file giaa164_supplemental_files.zip › Additional file 5-Fig. S3.pdf]

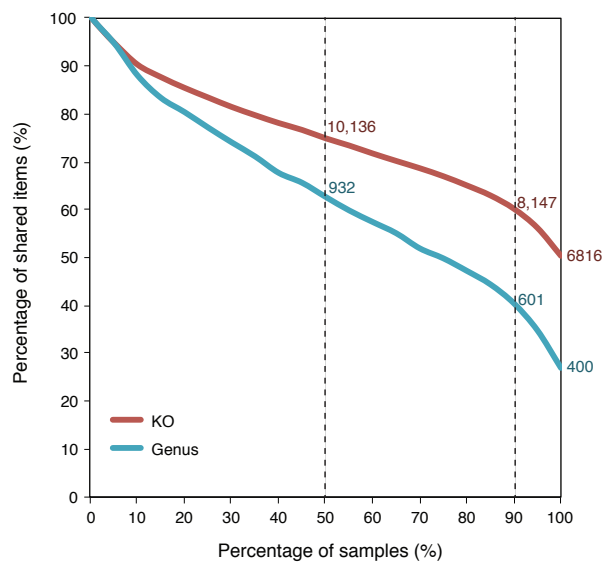

Supplement: giaa164_Supplemental_Files [file giaa164_supplemental_files.zip › Additional file 8-Fig. S5.pdf]

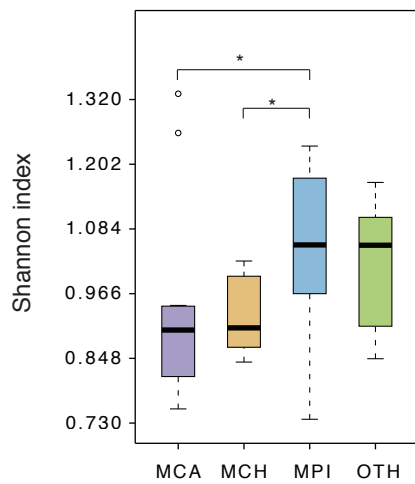

Supplement: giaa164_Supplemental_Files [file giaa164_supplemental_files.zip › Additional file 9-Fig. S6.pdf]
