# Supplementary material for: A microbial gene catalog of anaerobic digestion from full-scale biogas plants
Source: Gigascience. 2021 Jan 27;10(1):giaa164. doi: 10.1093/gigascience/giaa164 (PMC7842101; doi:10.1093/gigascience/giaa164)
Supplement: giaa164_Supplemental_Files [file Additional file 7-Fig. S4.pdf › Additional file 7-Fig. S4.pdf]

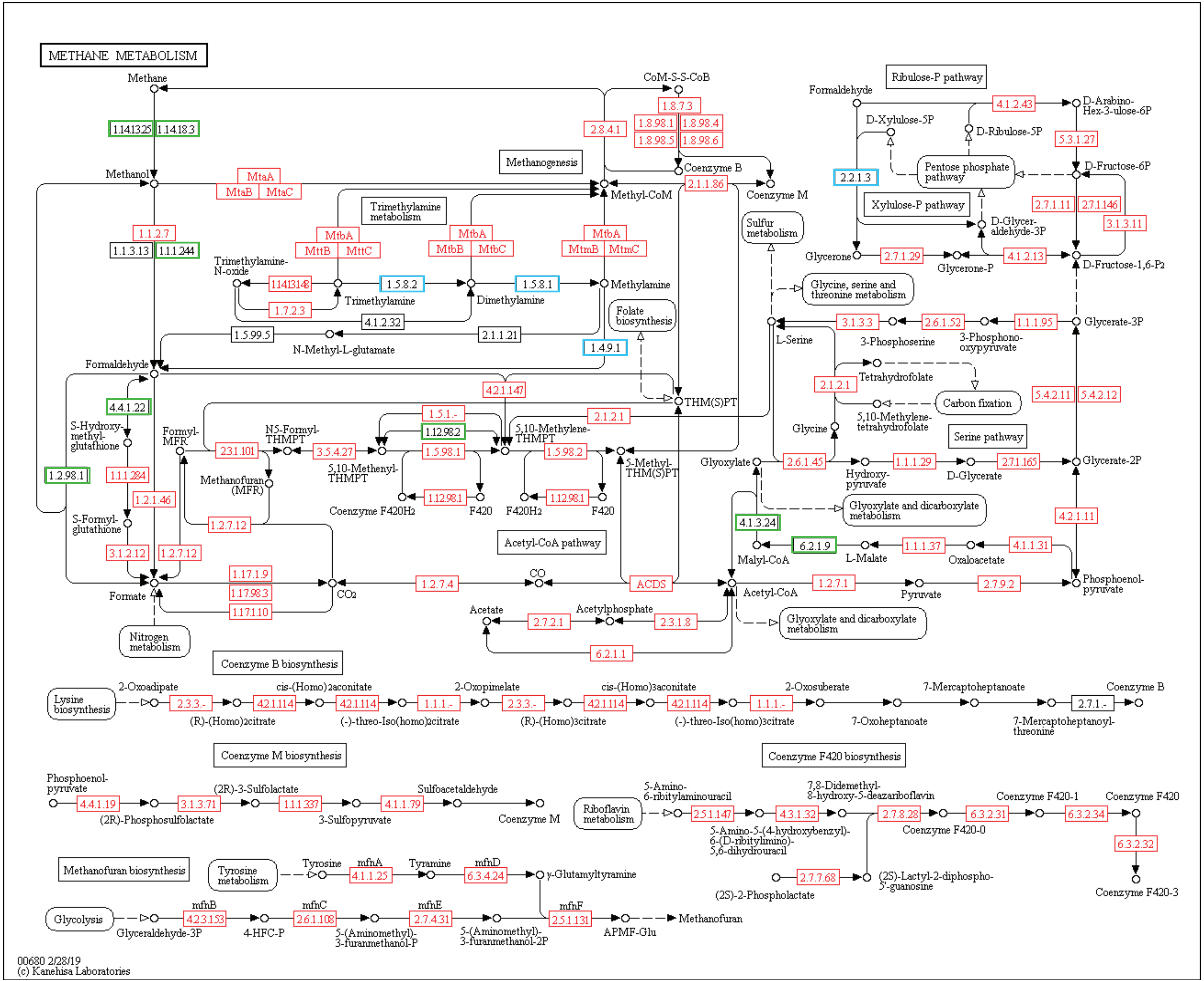

METHANE METABOLISM
Methane
1.14.13.25
1.14.18.3
Methanol
1.1.2.7
1.1.3.13
1.1.2.44
MtaA
MtaB
MtaC
Trimethylamine metabolism
Methanogenesis
CoM-S-S-CoB
1.8.7.3
1.8.98.1
1.8.98.4
1.8.98.5
1.8.98.6
2.8.4.1
Coenzyme B
2.1.1.86
Coenzyme M
Methyl-CoM
Sulfur metabolism
MttB
MttC
MtbA
MtbB
MtbC
MtmB
MtmC
Trimethylamine-N-oxide
1.14.13.148
1.7.2.3
Trimethylamine
1.5.8.2
Dimethylamine
1.5.8.1
Methylamine
4.1.2.32
1.5.99.5
N-Methyl-L-glutamate
2.1.1.21
1.4.9.1
Folate biosynthesis
Formaldehyde
4.4.1.22
S-Hydroxy-methyl-glutathione
1.2.98.1
1.1.1.284
1.2.1.46
S-Formyl-glutathione
3.1.2.12
1.2.7.12
Formyl-MFR
2.3.1.101
N5-Formyl-THMPT
3.5.4.27
5,10-Methenyl-THMPT
1.5.1.-
1.12.98.2
1.5.98.1
4.2.1.147
2.1.2.1
5,10-Methylene-THMPT
1.5.98.2
5-Methyl-THM(S)PT
THM(S)PT
Methanofuran (MFR)
Coenzyme F420H2
1.12.98.1
F420
F420H2
Acetyl-CoA pathway
Formate
CO2
CO
1.17.1.9
1.17.98.3
1.17.1.10
1.2.7.4
ACDS
Nitrogen metabolism
Acetate
2.7.2.1
Acetylphosphate
2.3.1.8
6.2.1.1
Acetyl-CoA
1.2.7.1
Pyruvate
2.7.9.2
Phosphoenol-pyruvate
Glyoxylate and dicarboxylate metabolism
Ribulose-P pathway
Formaldehyde
4.1.2.43
D-Arabino-Hex-3-ulose-6P
D-Xylulose-5P
D-Ribulose-5P
5.3.1.27
2.2.1.3
Pentose phosphate pathway
D-Fructose-6P
Xylulose-P pathway
2.7.1.11
2.7.1.146
3.1.3.11
D-Glycer-aldehyde-3P
Glycerone
2.7.1.29
Glycerone-P
4.1.2.13
D-Fructose-1,6-P2
Glycine, serine and threonine metabolism
L-Serine
3.1.3.3
3-Phosphoserine
2.6.1.52
3-Phosphono-oxypyruvate
1.1.1.95
Glycerate-3P
2.1.2.1
Tetrahydrofolate
Carbon fixation
5,10-Methylene-tetrahydrofolate
5.4.2.11
5.4.2.12
Glycine
Serine pathway
Glyoxylate
2.6.1.45
Hydroxy-pyruvate
1.1.1.29
D-Glycerate
2.7.1.165
Glycerate-2P
4.1.3.24
4.2.1.11
Malyl-CoA
6.2.1.9
L-Malate
1.1.1.37
Oxaloacetate
4.1.1.31
Coenzyme B biosynthesis
Lysine biosynthesis
2-Oxoadipate
2.3.3.-
(R)-(Homo)2citrate
4.2.1.114
cis-(Homo)2aconitate
(-)-threo-Iso(homo)2citrate
1.1.1.-
2-Oxopimelate
(R)-(Homo)3citrate
cis-(Homo)3aconitate
(-)-threo-Iso(homo)3citrate
2-Oxosuberate
7-Oxoheptanoate
7-Mercaptoheptanoate
7-Mercaptoheptanoyl-threonine
2.7.1.-
Coenzyme B
Coenzyme M biosynthesis
Phosphoenol-pyruvate
4.4.1.19
(2R)-Phosphosulfolactate
3.1.3.71
(2R)-3-Sulfolactate
1.1.1.337
3-Sulfopyruvate
4.1.1.79
Sulfoacetaldehyde
Coenzyme M
Coenzyme F420 biosynthesis
Riboflavin metabolism
5-Amino-6-ribitylaminouracil
2.5.1.147
5-Amino-5-(4-hydroxybenzyl)-6-(D-ribitylimino)-5,6-dihydrouracil
4.3.1.32
7,8-Didemethyl-8-hydroxy-5-deazariboflavin
2.7.8.28
Coenzyme F420-0
6.3.2.31
Coenzyme F420-1
6.3.2.34
Coenzyme F420
6.3.2.32
Coenzyme F420-3
(2S)-2-Phospholactate
2.7.7.68
(2S)-Lactyl-2-diphospho-5'-guanosine
Methanofuran biosynthesis
Tyrosine metabolism
Tyrosine
mfnA
4.1.1.25
Tyramine
mfnD
6.3.4.24
γ-Glutamyltyramine
Glycolysis
Glyceraldehyde-3P
mfnB
4.2.3.153
4-HFC-P
mfnC
2.6.1.108
5-(Aminomethyl)-3-furanmethanol-P
mfnE
2.7.4.31
5-(Aminomethyl)-3-furanmethanol-2P
mfnF
2.5.1.131
APMF-Glu
Methanofuran
00680 2/28/19
(c) Kanehisa Laboratories
