# Supplementary material for: A microbial gene catalog of anaerobic digestion from full-scale biogas plants
Source: Gigascience. 2021 Jan 27;10(1):giaa164. doi: 10.1093/gigascience/giaa164 (PMC7842101; doi:10.1093/gigascience/giaa164)
Supplement: giaa164_Response_to_Reviewer_Comments_Original_Submission [file giaa164_response_to_reviewer_comments_original_submission.pdf]

Author's Response To Reviewer Comments

Close

Response to the comments of the Editor and reviewers
Thank you for sending us the feedback on our manuscript A microbial gene catalog of anaerobic digestion from full-scale biogas plants [Paper # GIGA-D-20-00207].
We are now submitting a new version to address the remaining concerns of reviewers. Followings are our specific responses:

Response to Reviewer #1:
These data provide a nice addition to the current public availability of metagenomic data for anaerobic digestion, but the authors do not appear to have looked for existing biogas plant datasets to compare against or include in the catalog. As such I feel that there needs to be some major revisions to the work to show that they have made every effort to include existing data and therefore have a representative gene catalog of all biogas plants, not just those sampled here.
Response: We have downloaded 39 relevant metagenomes (580 Gb) derived from full-scale biogas plants, which located in Germany (22 samples), United Kingdom (12 samples), Spain (4 samples) and Sweden (1 sample) from NCBI, ENA, or MGnify database, and provided a more comprehensive microbial gene catalog of AD (C-MGCA), which containing 25,329,366 non-redundant genes (ftp://ftp.agis.org.cn/~fanwei/Anaerobic_digestion_metagenome).
We have added a paragraph of “To assess to what extent that MGCA could represent the microbial genes in full-scale BGPs, a more Comprehensive Microbial Gene Catalog of AD (C-MGCA) of full-scale biogas plants was constructed. Except for the 59 metagenomes that generated in this study (1,817 Gb), other 39 metagenomes (580 Gb) derived from full-scale biogas plants, which located in Germany (22 samples), United Kingdom (12 samples), Spain (4 samples) and Sweden (1 sample), were downloaded from NCBI, ENA, or MGnify database (Additional files 4: Table S2). All data were integrated and processed using the same pipeline for MGCA, and 25,329,366 non-redundant genes were generated for C-MGCA. Based on pairwise alignments of the two gene catalogs at gene level using BLAT (BLAT, RRID:SCR_011919) [22], we found that almost all genes in MGCA (99.99%) were shared by C-MGCA (with the criteria for shared genes that identity ≥ 95% and overlap ≥ 90% of the shorter genes), though C-MGCA only have 2,489,181 genes more than those of MGCA (Additional files 5: Fig. S3). In addition, six previously reported datasets derived from biogas plants [23-28] were processed using the same pipeline for MGCA and compared to the two gene catalogs. The results showed that only 52.3 ± 9.6% of genes in these datasets were shared by MGCA, while 99.5 ± 0.7% of genes were shared by C-MGCA (Additional file 6: Table S3), which were consistent with the fact that the data of the six datasets were used for constructing of C-MGCA. These results indicated that though MGCA contains a large proportion of genes in full-scale biogas plants, the gene coverage might be further improved by collecting more diversified samples, especially for those rare genes in specific types of AD process.” in the revised manuscript. (page 14-15, line 212-233).

(Line 320) "Here, we present the first comprehensive microbial gene catalog of anaerobic digestion (AD), …". Given that the catalog does not include data from any previous anaerobic digestion sequencing projects and <1% of large biogas plants in 1 country were sampled. I think it is naïve to describe this as a comprehensive gene catalog of AD. It maybe a comprehensive gene catalog of Chinese biogas plants?
Response: The reason why we used “comprehensive” is that we think that our gene catalog derived from digestate samples of diverse feedstocks, different temperature, and distributed widely in geographical regions. We agree to tune down the conclusion, and have deleted the word of “comprehensive”, and changed the sentence to “Here, we present a microbial gene catalog of anaerobic digestion (AD)” in the revised manuscript. (page 26, line 401).

There are other large sequencing datasets on biogas plants available in public domain databases such as the MGnify database or MG-RAST, e.g. In this study (released over 3 years ago) there are >600m clean reads from 12 samples https://www.ebi.ac.uk/metagenomics/studies/MGYS00001781. I am sure there will be others if the authors look for them. In order to state that this is a comprehensive gene catalog there must be some evidence that they have incorporated previous work.
Response: We have downloaded the dataset mentioned here from MGnify database, which containing

about 118 Gb of sequencing data (12 metagenomes). In addition, we also downloaded other 27 metagenomes derived from full-scale biogas plants from NCBI, ENA, or MGnify database. In summary, about 580 Gb (4,225 million) reads were downloaded. By integrating these data with our data, we generated a more comprehensive microbial gene catalog of AD (C-MGCA), which containing 25,329,366 non-redundant genes.
(ftp://ftp.agis.org.cn/~fanwei/Anaerobic_digestion_metagenome).

Linked to the inclusion of other datasets, there should be a better description of the methods used for abundance calculations and normalization of abundance across samples within this study and those from external studies to be included.
Response: To calculate the relative gene abundance of each sample, the clean reads of each sample were mapped separately onto the gene catalog by BWA-MEM, and the reads with alignment length ≥ 50 bp and identity > 95% were defined as qualified reads, which were used for calculating the relative gene abundance.
We have added the description of the methods in the revised manuscript: "The relative gene abundance of MGCA were calculated using the qualified reads [20, 21]. Briefly, for each sample, total number of reads mapped to all genes (TA) equal to the count of qualified reads, total number of reads mapped to one gene (TO) equal to the count of qualified reads mapped to the gene. At last, the normalized gene abundance (NGA) for each sample was calculated according the following formula: NGA = TO / (GL / 1,000) / (TA / 10,000,000); GL means the length of the gene.". (page 13, line 196-201).

(line 186) "With the mapped reads, we calculated the relative gene abundance as previously described [20, 21]. In addition, 4,360 genes were removed from the gene catalog as they have no read mapped". I am not an expert statistician but I have concerns that the relative abundance of genes and species in this way is inaccurate. I also do not have access to the cited manuscript (Nature paywall), so I cannot see how the normalization was done between samples. The authors should include a summary of their methods (better still add them to protocols.io). I would appreciate someone with a greater experience in statistics to take a look at these methods to confirm their suitability for the purpose.
Response: Here we define "no read mapped" as no qualified read (with alignment length ≥ 50 bp and identity > 95%) in any sample can be mapped to these 4,360 genes, so there is no matter with relative abundance or normalization method among samples. These genes may be derived from wrong assembly or extreme low abundance, so we think it is better to filter them from the reference gene catalog. In addition, we have added the method for calculating and normalizing the relative gene abundance to protocols.io (dx.doi.org/10.17504/protocols.io.bpivmke6).
The sentence has been changed to "The clean reads of each sample were mapped onto this initial gene catalog by BWA-MEM, and a total of 80.66% of qualified reads (with alignment length ≥ 50 bp and identity > 95%) could be mapped. However, there were 4,360 genes having no qualified read mapped in any sample, which may be derived from wrong assembly or extreme low abundance, and they were removed from the gene catalog". (page 12-13, line 188-193).

In addition, there are a number of minor revisions that should also be considered:

1 - The data description states "of 110,975 biogas plants been established in China, including 6,737 large-scale and 34 extra large-scale biogas plants", but then goes on to describe the 56 they selected as "full-scale", does that mean those 56 are large or extra-large?
Response: In this study, 56 biogas plants comprise 10 extra large-scale, 15 large-scale, 12 medium-scale, 18 small-scale biogas plants and 1 rural household digester. In addition, "full-scale" refers to production-oriented, which is relative to laboratory-scale to pilot-scale, while "large" and "extra-large" are classification of biogas plants according to the volume of digesters.
To reduce the confusion caused by using "large" and "extra-large" in the text, we have deleted the words "According to national statistics, by the end of 2015, there was a total number of 110,975 biogas plants been established in China, including 6,737 large-scale and 34 extra large-scale biogas plants" in the revised manuscript.

2 - (line 133)
It would be nice to have the gel images for "The integrity of DNA extracts was checked on 0.7% (w/v) agarose gel with GelRed nucleic acid gel stain".
Response: We have added a gel image in supplementary files (Additional file 3: Fig S2). In the gel image, we can see an obvious concentrated DNA band and the band length > 15 kb (the highest band length of the marker is 15 kb). (Additional file 3: Fig S2)
We have added a sentence "and DNA samples with obvious concentrated DNA band and the fragment length of the band > 15 kb were used for further analysis (Additional file 3: Fig S2)" in revised

manuscript. (page 9, line 134-136).

3 - (line 163)
"The Illumina raw reads were cleaned by trimming the adapter sequences and low-quality regions using two in-house software clean_adapter and clean_lowqual [14]". Ref[14] = Clean_adapter and clean_lowqul on github. https://github.com/fanagislab/DBG_assembly/tree/master/clean_illumina.
This github link gives an ERROR 404, we need access to this, and some indication of its suitability to the task, why was an in-house script written instead of using an existing tool?
Response: We made a mistake in adding an extra space in the link website (the space between fanagislab/ and DBG_assembly/), and we have deleted it in the revised manuscript.
The software clean_adapter and clean_lowqual were developed by our team, and the functions were to filter adaptor and low quality sequences, respectively. Since the functions to filter adaptor and low quality sequences were simple, and the results generated from the two software were comparable to those from some existing tools (eg: FastQC). In addition, the software has been used by our team and colleagues for many years, and cited in many published papers (Nat Commun, 2020;11:340; Microbiome, 2018;6:211; GigaScience; 2018;7:1). So, we prefer to use the two software to clean the raw reads in our research.

4 - (line 194)
"The rarefaction curve approached saturation with the increase of sample number (Fig. 1a), suggesting that our gene catalog covered the vast majority of microbial genes in full-scale BGPs." This is a little misleading, the saturation of sampling is not indicative of the level of coverage of all BGPs, only that you have captured the majority of genes present within your sampled BGPs. You should adjust the sentence to: "The rarefaction curve approached saturation with the increase of sample number (Fig. 1a), suggesting that our gene catalog covered the vast majority of microbial genes to be found in the 56 full-scale BGPs sampled in this study."
Response: We have revised the sentence according to reviewer’s suggestion to “The rarefaction curve approached saturation with the increase of sample number (Fig. 1a), suggesting that our gene catalog covered the vast majority of microbial genes to be found in the 56 full-scale BGPs sampled in this study”. (page 13-14, line 203-206).

More comparison to existing datasets would need to be done to evaluate the coverage of the catalog for all BGPs. Even the one example that has been compared to, shows that ~40% of that comparison dataset are not present in the catalog.
Response: Other five existing datasets derived from full-scale biogas plants were compared to the gene catalog, and the results showed that only 52.3 ± 9.6% of genes were shared by our gene catalog (MGCA) (Additional file 6: Table S3).
We have revised the sentence to “In addition, six previously reported datasets derived from biogas plants [23-28] were processed using the same pipeline for MGCA and compared to the two gene catalogs. The results showed that only 52.3 ± 9.6% of genes in these datasets were shared by MGCA, while 99.5 ± 0.7% of genes were shared by C-MGCA (Additional file 6: Table S3), which were consistent with the fact that the data of the six datasets were used for constructing of C-MGCA” in the revised manuscript. (page 15, line 225-230; Additional file 6: Table S3).

5 - (Line 263)
"represented by 400 genera, 6,816 KOs (Additional file 5: Fig. S4), accounting for about 98.76% and 99.39% of the total relative abundance of annotated genera and KOs". This sentence doesn't make sense to me, please clarify its meaning.
Response: We have revised the sentence to “In the current study with the in-depth metagenomic sequencing of diverse full-scale BGPs, we found 400 genera and 6,816 KOs were shared by all the investigated samples (Additional file 8: Fig. S5), which accounted for about 98.76% and 99.39% of the total relative abundance of annotated genera and KOs, respectively”. (page 18, line 279-283).

6 - Fig 5a - Why were "Other" category BGPs excluded from this analysis?
Response: We have added “Other” category BGPs in PCoA analysis (Fig 5a) in revised Fig 5. Since group MCA, MCH, and MPI includes BGPs fed with cattle manure, chicken manure, and pig manure, respectively. However, to increase the completeness of the gene catalog, BGPs fed with diverse feedstocks (such as bear manure, pigeon manure, mixture of cattle manure and straw, mixture of pig manure and sewage water) were also included, and these BGPs formed group OTH. So, it is hard to generalize a common characteristic of this group. Correspondingly, PCoA analysis (Fig 5a) also showed that samples from group OTH (green color) were distributed in other groups.

7 - (line 281)
Section on "Microbial functional differentiation among BGPs with different feedstocks". This section is also not required as part of a data-note describing the data as it goes into the analysis of the data, it could be removed entirely. As it in this section is lacking in any statistical evaluation and the discussion makes bold statements about some groups having higher/lower relative abundance of things that when you look at the Fig5 are not at all obvious with the interquartile ranges overlapping considerably. e.g. "the relative abundance of genes involved in the hydrolysis of proteins was much higher in MCH (Fig. 5c)". Fig 5c shows an increase median abundance value for MCH, but all 3 have drastically overlapping IQRs. Some indication of the significance or robustness of these findings would be appropriate. Please also see my previous comment on the suitability of the abundance calculations and normalization.
Response: We hope to retain this part in the manuscript, as it provides a basic analysis of the dataset, though we also agree to remove it if the reviewer persist that this part should be removed from the manuscript.
In the revised manuscript, we added Wilcox rank sum test among different groups, and marked "*" in the Figure 5 (b, c, d, and e) when differences were significant ($p < 0.05$) between the two groups. For example, for genes involved in lignin, hemicelluloses, and cellulose degradation, the relative abundances were significantly ($p < 0.05$) higher in MCA than those in MPI.

8 - If analysis of the data is to be included, another point that should be addressed is that of the pH of the plants. One of the major environmental influences on bacterial activity in anaerobic digestion (AD) is pH (See here for ref 10.1186/1754-6834-5-39.), the pH of the reactors is recorded in the metadata, but there has been no mention of its effects within the discussion, how does the pH correlate with the 4 reactor classes MCA (13 cattle manure BGPs), MCH (6 chicken manure BGPs), MPI (27 pig manure BGPs), and OTH (10 BGPs with other substrates)? How does pH effect beta diversity of samples? etc...
Response: The pH is an important process parameter for the management of the biogas processes. In this study, the median pH value of group MCA and group MCH were higher than those of group MPI and OTH. However, the differences among the four groups were not significant (Wilcox rank sum test; $P > 0.05$). Similarly, the differences of beta-diversity (Bray-Curtis distance) among the four groups were also not significant (Wilcox rank sum test; $P > 0.05$), and we cannot find correlations between pH value and beta-diversity of the samples. In addition, Redundancy analysis (RDA) at the genus level revealed that pH was also an important determinant parameter that influenced the microbial composition, though it is hard to differentiate the groups of MCA and MPI.

Response to Reviewer #2:
This manuscript reports the metagenomic sequencing of a large number of anaerobic digestion (AD) plants in China.

The methods for carrying out this work are appropriate and generally well described. However, there are a few points that should be addressed:
- It is not clear what the rationale or effect of the freeze thaw steps were on DNA extraction.
Response: Freeze-thaw is a physical method for cell disruption, and added it prior to the standard protocol can increase DNA yield. In our test, the yield of DNA increased by 16.9% by adding an extra repeated freeze-thaw step.
In addition, we have revised the sentence to "To increase DNA yield, an extra physical cell disruption step of repeated freeze-thaw (four times of alternating between 65℃ and liquid nitrogen for 5 min) was employed prior to the standard protocol" in the revised manuscript. (page 9, line 130-132).

- There is no mention of what the quality criteria were that DNA samples had to pass in order to be sequenced.
Response: For integrity, there should be an obvious concentrated DNA band and the band length > 15 kb in electrophoresis graph. For DNA quality and quantity, the ratio of A260/280 should between 1.8 and 2.0 and dsDNA concentration should higher than 20 ng/μL.
We have revised the sentence to "After DNA quality checks, the three replicates of high-quality DNA (band length > 15 kb, A260/280 1.8-2.0, dsDNA concentration > 20 ng/μL) of each sample were pooled for library construction." in the revised manuscript. (page 9, line 138-140).

- There do not appear to be biological replicates of the samples, so it is not clear how representative the samples are of each digester.
Response: We have 14, 7, and 28 biological replicates for samples collected from biogas plants of group MCA (fed with cattle manure), MCH (fed with chicken manure), and MPI (fed with pig manure), respectively, and the conclusions derived from these replicated samples were strengthened by statistical analysis. In addition, to acquire the representative sample for each digester, the digestate in each

digester was stirred thoroughly before sampling.

- It is not clear whether technical replicates of DNA extraction were carried out, or how consistent this process was.
Response: There were three technical replicates of DNA extraction in this study, and it was described in "Genomic DNA was extracted in triplicate using the PowerSoil DNA Isolation Kit (cat. no. 12888-100; MoBio Laboratories Inc., USA) according to the manufacturer's protocol". (page 9, line 128-130)
In addition, to maintain the consistence of each replicate of DNA extraction, three replicates of each sample were performed in parallel by the same person at the same time. At last, the three replicates of high-quality DNA of each sample were pooled for library construction.

I'm not sure I agree with some of the conclusions:
- The authors claim this is a "comprehensive microbial gene catalog of anaerobic digestion" (line 320), but Fig S2 shows that their dataset does not include 34% of a related, but smaller dataset. Surely if these genes are missing from the current work then this cannot be "comprehensive"? It could perhaps be claimed to be "comprehensive for AD plants located in China", although with an N50 of ~ 4 kb and 56% of genes reported by the authors as less than full length, this is also perhaps overstating their claim.
Response: We have deleted the word of "comprehensive", and the reason why we used "comprehensive" is that we think that our gene catalog derived from digestate samples of diverse feedstocks, different temperature, and distributed widely in geographical regions.
We have revised the sentence to "Here, we present a microbial gene catalog of anaerobic digestion (AD)," in the revised manuscript. (page 25, line 401).

- The AD core genera claim is difficult to substantiate without showing that the genera identified are different from those found for the core genera in the feedstock i.e. those found in cattle, chicken and pig gut microbiomes. It would be good to add such a comparison using published data.
Response: We agree with the reviewer's suggestion that the core genera in AD should different from those found for the core genera in the feedstock. However, because of the similar anaerobic environment in animal gut and digester, there was partial overlap between them in microbial composition. In addition, to increase the credibility of our results for the core genera, we have changed the requirements for defining core microbiome from "present in more than 80% of the studied samples" to "present in all investigated samples" in revised manuscript.
We have revised the words to "Thus, we defined core microbes by including genera that were both abundant and prevalent (most abundant top 30 bacterial genera and top 5 archaeal genera that were detected in all studied samples). As a result, only Bacteroides and Clostridium (Fig. 4), within the order of Bacteroidales and Clostridiales, were identified as core microbes. The result was consistent with previous study which detected Bacteroidales and Clostridiales from all 29 full-scale BGPs by 16S rRNA gene amplicon sequencing [8]. However, we should notice that Bacteroides and Clostridium were also the abundant genera in cattle, chicken and pig gut [20, 32, 37]" in revised manuscript. (page 19, line 286-294).

The quality of the language in the manuscript is generally good. Statistics are appropriately used.

Additional clarification would be useful where the authors claim 56 "full-scale" biogas plants were sampled, but don't define what "full-scale" represents. They mention "large" and "extra large" scales, but none of these terms are defined with respect to volume. Samples are taken from digesters ranging from 12 m3 to 8000 m3. If 12 m3 is considered "full scale" (this is not the case in many parts of the world), what range of reactors are "large scale" compared to "extra-large" scale?
Response: "Full-scale" refers to production-oriented, which is relative to laboratory-scale to pilot-scale, while "large" and "extra-large" are classification of biogas plants according to the volume of digesters by Chinese.
Based on the indicators in Classification Standard of Biogas Plant Scale (NY/T 667-2011) issued by Ministry of Agriculture and Rural Affairs, People's Republic of China, "extra-large scale" should satisfied the conditions that the volume of individual digester (V1) ≥ 2500 m3 and total volume of digesters (V2) ≥ 5000 m3; while 2500 > V1 ≥ 500 m3 and 5000 > V2 ≥ 500 m3 for "large scale", 500 > V1 ≥ 300 m3 and 1000 > V2 ≥ 300 m3 for "medium scale"; 300 > V1 ≥ 20 m3 and 600 > V2 ≥ 20 m3 for "small scale".
In this study, 56 biogas plants mentioned in this study include 10 extra large-scale, 15 large-scale, 12 medium-scale, 18 small-scale biogas plants and 1 rural household digester (12 m3). However, 12 m3 rural household digester can also be considered as "full scale", which is not lab- or pilot scale. In addition, to more accurately, the rural household digester should term "full-scale anaerobic digester".

To reduce the confusion caused by using "large" and "extra-large" in the text, we have deleted the words "According to national statistics, by the end of 2015, there was a total number of 110,975 biogas plants been established in China, including 6,737 large-scale and 34 extra large-scale biogas plants" in the revised manuscript.

There is little operational information concerning the digesters from which the metagenomes were sequenced. There is no indication of hydraulic retention time, feedstock composition (e.g. COD), or biogas volume / composition, which means little biological insight is possible, as it is impossible to correlate how well each digester was functioning and therefore how well the metagenome associated with each digester was performing. It could be that many of the species identified do not contribute to AD but are rather competing for resources within the digesters, or not metabolically active at all.
Response: We have added other process parameter (hydraulic retention time, HRT), physicochemical characteristics of feedstock (total nitrogen, TN; total carbon, TC; and total solid, TS) and intermediate metabolites (total ammonia nitrogen, TAN; and VFAs) in Table S1. In addition, redundancy analysis (RDA) based on these parameters revealed that operation temperature and TAN were primarily determinant parameters that influenced the microbial composition (Fig. S7).
We have added the paragraph of "In addition, various parameters in AD also have important effects on shaping microbial communities, and several process parameters (operation temperature; pH; hydraulic retention time, HRT, and reactor volume), physicochemical characteristics of feedstock (total nitrogen, TN; total carbon, TC; and total solid, TS) and intermediate metabolites (total ammonia nitrogen, TAN; and VFAs) for all BGPs (Additional file 2: Table S1) from the groups MCA, MCH, MPI, and OTH were analyzed. Redundancy analysis (RDA) at the genus level revealed that operation temperature and TAN were primarily determinant parameters that influenced the microbial composition, and then followed by TS, acetate, total VFAs, acetate, TN, and pH (Additional file 12: Fig. S7). The result was consistent with a previous study that TAN and digester temperature were identified as the main contributing factors to cluster formation [8]" in revised manuscript. (page 22-23, line 346-357).

Response to Reviewer #3:
The paper describes an impressively large sequencing project involving the microbiome of 59 full scale biogas facilities. The collection of more than 22 thousand AD genes is useful for further research, this justifies the work done. However, the paper draws disappointingly poor conclusions at the end of the data analysis. There is no innovative, "take home", new message for the readers. The only aspect discussed briefly is the clustering of the metagenomes according to the employed AD substrate, which is not surprising or novel in AD microbiology at all. If this is indeed the only conclusion that the authors could extract from this work, the paper is not suitable and not acceptable for publication in GigaScience, which expects novel ideas, discoveries to be communicated from the metagenomic studies. The paper needs very thorough revision before it can be considered for publication in GIGA.
Response: We have made some major revisions according to reviewers' suggestions:
(1) downloaded other 39 relevant metagenomes (580 Gb) derived from full-scale biogas plants, which located in Germany, United Kingdom, Spain and Sweden, and constructed a more comprehensive microbial gene catalog of full-scale biogas plants (C-MGCA, 25,329,366 genes);
(2) added metagenome binning analysis in the revised manuscript, and constructed 2,426 metagenome-assembled genomes (MAGs).

Specific major comments:
1. You should check if a number of other process parameters, e.g. reactor size and geometry, mixing, additional substrate biomasses, residence times, loading rates, biogas yields, product methane contents, etc. would correlate with the metagenome data. Alterations caused by feeding the AD reactors with various types of animal manure is trivial and expectable. Did you screen co-fermentation systems?
Response: In the revised manuscript, the correlation of operational parameters (hydraulic retention time, pH, reactor volume, and operation temperature), feedstock compositions (total nitrogen, TN; total carbon, TC; and total solid, TS) and intermediate metabolites (total ammonia nitrogen, TAN; and volatile fatty acids, VFAs) of anaerobic digestion were analyzed by Redundancy analysis (RDA) at the genus level. The result revealed that operation temperature, TAN, TS, Acetate, and total VFAs were primarily determinant parameters that influenced the microbial composition (Fig. S7).
In addition, there were 7 biogas plants were processed with co-fermentation systems, which co-digested with animal manure and straw or other materials, and the PCoA results indicated that microbial compositions were more similar to the those of the plants feed with corresponding animal manure.
We have added the paragraph of "In addition, various parameters in AD also have important effects on shaping microbial communities, and several process parameters (operation temperature; pH; hydraulic retention time, HRT, and reactor volume), physicochemical characteristics of feedstock (total nitrogen, TN; total carbon, TC; and total solid, TS) and intermediate metabolites (total ammonia nitrogen, TAN;

and VFAs) for all BGPs (Additional file 2: Table S1) from the groups MCA, MCH, MPI, and OTH were analyzed. Redundancy analysis (RDA) at the genus level revealed that operation temperature and TAN were primarily determinant parameters that influenced the microbial composition, and then followed by TS, acetate, total VFAs, acetate, TN, and pH (Additional file 12: Fig. S7). The result was consistent with a previous study that TAN and digester temperature were identified as the main contributing factors to cluster formation [8]" in revised manuscript. (page 22-23, line 346-357).

2. You should compare your data with literature data more extensively regarding the results obtained by others using sequencing and bioinformatics methods, substrates, geographical sites, temperatures, etc. There have been numerous relevant studies published in Europe (Germany, Austria, Hungary) to consider.
Response: In the revised manuscript, we compared our gene catalog with more published datasets, the results showed that only 52.3 ± 9.6% of genes from these published datasets were shared by our gene catalog, and we have changed the words to "In addition, six previously reported datasets derived from biogas plants [23-28] were processed using the same pipeline for MGCA and compared to the two gene catalogs. The results showed that only 52.3 ± 9.6% of genes in these datasets were shared by MGCA, while 99.5 ± 0.7% of genes were shared by C-MGCA (Additional file 6: Table S3), which were consistent with the fact that the data of the six datasets were used for constructing of C-MGCA" in revised manuscript. (page 15, line 225-230).
In addition, we also added more comparison about the "core microbiome" in revised manuscript.

3. In the bioinformatics workflow you should compare results obtained by using reference databases other than NCBI-NR.
Response: In this study, NCBI-NR database were only used for taxonomic annotation of the microbial gene catalog by using software CARMA3, which using NCBI-NR database as default database. (page 15-16, line 236-239). In addition, functional annotation was performed using KEGG and dbCAN databases.

4. Similarly, it is a major flaw that genome-based evaluation (binning) of the data is not included. This would validate the read-based bioinformatics. In addition, binning would allow you species level resolution of the microbiota, which could be more informative than looking at the microbiomes at genus level.
Response: Metagenome binning analysis was added in the revised manuscript, and 2,426 metagenome-assembled genomes (MAGs) were constructed, including 1,205 MAGs (49.7%) with completeness ≥ 90% and contamination ≤ 5%. Taxonomic annotation revealed that 96.08% and 3.92 % of MAGs were assigned to Bacteria and Archaea, respectively. In addition, Firmicutes (38.25%), Bacteroidetes (21.89%), and Proteobacteria (5.03%) were the dominant phyla in these MAGs.
We have added a part of "Construction of metagenome-assembled genomes" in revised manuscript. (page 23-26, line 359-398).

Additional corrections needed:
1. L.56. There is no substantial "shortage" of fossil fuels. It is the environmental global climate change effect that drives research into renewable.
Response: We have revised the sentence to "In the context of global climate change, biogas as a renewable energy form has become increasingly attractive to the world's attention in recent years" in revised manuscript. (page 4, line 55-56).

2. L.62. How do you calculate "over the last two decades"? Industrial biogas technology older than that.
Response: We have delete "over the last two decades" in the revised manuscript.

3. L.67-69. Adjust the tenses of the verbs.
Response: We have changed the word "were" to "are" in revised manuscript. (page 5, line 69).

4. L.80-81. There have been many more studies on large scale AD microbiomes.
Response: We have revised the sentence to "However, most of these studies have a relatively small number of full-scale anaerobic digesters or with a small amount of sequencing data" in the revised manuscript. (page 5-6, line 79-81).

5. L.87-88. L. 89. Grammar!
Response: Thank you for your suggestion. To reduce the confusion caused by using "large" and "extra-large" in the sentence, we have deleted the words "According to national statistics, by the end of 2015, there was a total number of 110,975 biogas plants been established in China, including 6,737 large-scale and 34 extra large-scale biogas plants" in the revised manuscript.

6. L.93. "Ambient" temperature varies probably a great deal across China. What are the real values?
Response: We have added the real values of ambient temperature (at the time of sampling) to the Additional file 2: Table S1, and revised the sentence to "All plants were operated at ambient temperature (14-31.3°C at the time of sampling) or mesophilic (35-45°C) conditions" in the revised manuscript. (page 6, line 90-92).

7. L.198. "most of the genes were shared"… Fig 1.b. does not corroborate this statement.
Response: We have revised the sentence to "and found that only a small proportion of genes (less than 12%) were unique in each of the four groups (Fig. 1b)" in the revised manuscript. (page 14, line 208-209).

8. L.199. Which are "widely existed" genes?
Response: We expected to express the meaning of "common microbial functions", which contains the functions like in production of methane.
In addition, we have revised the sentence to "In addition, we compared the genes assigned to MCA (15,346,132 genes), MCH (9,707,833 genes), MPI (18,662,450 genes), and OTH (15,507,636 genes), and found that only a small proportion of genes (less than 12%) were unique in each of the four groups (Fig. 1b), which revealed that common microbial functions in AD were shared among different BGPs" in revised manuscript. (page 14, line 206-211).

9. L.269. What justifies the presence of "more than 80%" as a core microbiome member? Core microbiome means the collection of microbes present in ALL investigated samples.
Response: We have changed the requirements for defining core microbiome from "in more than 80% of the studied samples" to "present in all investigated samples". Though more microbes can be listed as core microbiome with the condition of "more than 80% samples", it is hard to justify these extra microbes as core microbiome member at this condition. In the revised manuscript, we found only Bacteroides and Clostridium (Fig. 4) were detected in all investigated samples.

10. L.304 and 307. The manures of the various animals are rich in these substances because their FEED is different from each other!
Response: We have added the words "These results are consistent with the fact that the manures of the various animals are rich in these substances because their feed is different from each other." in the revised manuscript. (page 21-22, line 332-334).

11. L.329. "consortium of …genes". Genes do not form a consortium.
Response: We have changed the word "genes" to "microbes", and corrected the sentence to "Compared to the published microbial gene catalogs of different ecosystems such as soil, ocean, animal gut and rumen [20, 32, 54-57], biogas plants are man-made extremely anaerobic ecosystems where AD is performed by a complex consortium of anaerobic microbes.". (page 26, line 408-411).

Close
