# Supplementary material for: A microbial gene catalog of anaerobic digestion from full-scale biogas plants
Source: Gigascience. 2021 Jan 27;10(1):giaa164. doi: 10.1093/gigascience/giaa164 (PMC7842101; doi:10.1093/gigascience/giaa164)
Supplement: giaa164_Response_to_Reviewer_Comments_Revision_1 [file giaa164_response_to_reviewer_comments_revision_1.pdf]

Author's Response To Reviewer Comments

Close

Response to the comments of the Editor and reviewers
Thank you for sending us the feedback on our manuscript A microbial gene catalog of anaerobic digestion from full-scale biogas plants [Paper # GIGA-D-20-00207].
We are now submitting a new version to address the remaining concerns of reviewers. Followings are our specific responses:

Response to Reviewer #1:
I would like to thank the authors for addressing all of my points in there response to reviewers document this made the process of review much easier.

I can confirm that the revisions do address all of my concerns and I am happy to now recommend this manuscript for acceptance.

The document would benefit from a fresh set of eyes checking over the wording and grammar and I list here a couple of examples that could be made easier to read/understand:

(line 55)
"In the context of global climate change, biogas as a renewable energy form has become increasingly attractive to the world's attention in recent years." - > "In the context of global climate change, biogas as a renewable energy form has increasingly drawn the world's attention in recent years.
Response: We have revised the sentence according to reviewer's suggestion to "In the context of global climate change, biogas as a renewable energy form has increasingly drawn the world's attention in recent years". (page 4)

(line 134)
The integrity of DNA extracts was checked on 0.7% (w/v) agarose gel with GelRed nucleic acid gel stain (cat. no. 41003; Biotium, USA), and DNA samples with obvious concentrated DNA band and the fragment length of the band > 15 kb were used for further analysis (Additional file 3: Fig S2).
->
The integrity of DNA extracts was checked on 0.7% (w/v) agarose gel with GelRed nucleic acid gel stain (cat. no. 41003; Biotium, USA). DNA samples showing obvious concentrated DNA bands >15 kb in size were used for further analysis (Additional file 3: Fig S2).
Response: We have revised the sentence according to reviewer's suggestion to "The integrity of DNA extracts was checked on 0.7% (w/v) agarose gel with GelRed nucleic acid gel stain (cat. no. 41003; Biotium, USA). DNA samples showing obvious concentrated DNA bands > 15 kb in size were used for further analysis". (page 9)

In addition, we have made other revisions about the wording and grammar:

1. (line 63) Anaerobic digestion includes four sequential metabolic steps, namely hydrolysis, acidogenesis, acetogenesis, and methanogenesis, and are performed by a complex consortium of bacteria and archaea [4, 5].
->
Anaerobic digestion includes four sequential metabolic steps, namely hydrolysis, acidogenesis, acetogenesis, and methanogenesis, and is performed by a complex consortium of bacteria and archaea [4, 5]. (page 4-5)

2. (line 138) After DNA quality checks, the three replicates of high-quality DNA (band length > 15 kb, A260/280 1.8-2.0, dsDNA concentration > 20 ng/μL) of each sample was pooled for library construction.
->
After DNA quality checks, the three replicates of high-quality DNA (band length > 15 kb, A260/280 1.8-2.0, dsDNA concentration > 20 ng/μL) of each sample were pooled for library construction. (page 9)

3. (line 330) Besides, the relative abundance of genes involved in the hydrolysis of proteins was much higher in MCH (Fig. 5c), which is consistent with the relatively high protein content of chicken manure [45-47].
->
Besides, the relative abundance of genes involved in the hydrolysis of proteins was much higher in MCH (Fig. 5c), which is associated with the relatively high protein content of chicken manure [45-47]. (page 21)

4. (line 346) In addition, various parameters in AD also have important effects on shaping microbial communities, and several process parameters (operation temperature; pH; hydraulic retention time, HRT, and reactor volume), physicochemical characteristics of feedstock (total nitrogen, TN; total carbon, TC; and total solid, TS) and intermediate metabolites (total ammonia nitrogen, TAN; and VFAs) for all BGPs (Additional file 2: Table S1) from the groups MCA, MCH, MPI, and OTH were analyzed.
->
In addition, various parameters in AD also have important effects on shaping microbial communities. Several process parameters (operation temperature; pH; hydraulic retention time, HRT, and reactor volume), physicochemical characteristics of feedstock (total nitrogen, TN; total carbon, TC; and total solid, TS) and intermediate metabolites (total ammonia nitrogen, TAN; and VFAs) for all BGPs (Additional file 2: Table S1) from the groups MCA, MCH, MPI, and OTH were analyzed. (page 22-23)

5. (line 352) Redundancy analysis (RDA) at the genus level revealed that operation temperature and TAN were primarily determinant parameters that influenced the microbial composition, and then followed by TS, acetate, total VFAs, acetate, TN, and pH (Additional file 12: Fig. S7). The result was consistent with a previous study that TAN and digester temperature were identified as the main contributing factors to cluster formation [8].
->
Redundancy analysis (RDA) at the genus level revealed that operation temperature and TAN were primarily determinant parameters that influenced the microbial composition, and then followed by TS, acetate, total VFAs, TN, and pH (Additional file 12: Fig. S7). The result was agreed with a previous finding that TAN and digester temperature were identified as the main contributing factors to cluster formation [8]. (page 23)

6. (line 407) In addition, we also provided 2,426 MAGs derived from full-scale biogas plants.
-> Additionally, we also provided 2,426 MAGs derived from full-scale biogas plants. (page 26)

Close
