# Supplementary material for: A microbial gene catalog of anaerobic digestion from full-scale biogas plants
Source: Gigascience. 2021 Jan 27;10(1):giaa164. doi: 10.1093/gigascience/giaa164 (PMC7842101; doi:10.1093/gigascience/giaa164)
Supplement: giaa164_Reviewer_1_Report_Original_Submission — Christopher Hunter, Ph.D. -- 7/17/2020 Reviewed [file giaa164_reviewer_1_report_original_submission.pdf]

Reviewer Report

Title: A microbial gene catalog of anaerobic digestion from full-scale biogas plants

Version: Original Submission **Date:** 7/17/2020

Reviewer name: Christopher Hunter, Ph.D.

Reviewer Comments to Author:

These data provide a nice addition to the current public availability of metagenomic data for anaerobic digestion, but the authors do not appear to have looked for existing biogas plant datasets to compare against or include in the catalog. As such I feel that there needs to be some major revisions to the work to show that they have made every effort to include existing data and therefore have a representative gene catalog of all biogas plants, not just those sampled here.
(Line 320) "Here, we present the first comprehensive microbial gene catalog of anaerobic digestion (AD), "
Given that the catalog does not include data from any previous anaerobic digestion sequencing projects and <1% of large biogas plants in 1 country were sampled I think it is naÃ¯ve to describe this as a comprehensive gene catalog of AD. It maybe a comprehensive gene catalog of Chinese biogas plants?
There are other large sequencing datasets on biogas plants available in public domain databases such as the MGnify database or MG-RAST, e.g. In this study (released over 3 years ago) there are >600m clean reads from 12 samples
https://www.ebi.ac.uk/metagenomics/studies/MGYS00001781 I am sure there will be others if the authors look for them. In order to state that this is a comprehensive gene catalog there must be some evidence that they have incorporated previous work.
Linked to the inclusion of other datasets, there should be a better description of the methods used for abundance calculations and normalisation of abundance across samples within this study and those from external studies to be included.
(line 186)
"With the mapped reads, we calculated the relative gene abundance as previously described [20, 21]. In addition, 4,360 genes were removed from the gene catalog as they have no read mapped. "
I am not an expert statistician but I have concerns that the relative abundance of genes and species in this way is inaccurate. I also do not have access to the cited manuscript (Nature paywall) so I cannot see how the normalisation was done between samples. The authors should include a summary of their methods (better still add them to protocols.io).
I would appreciate someone with a greater experience in statistics to take a look at these methods to confirm their suitability for the purpose.
In addition there are a number of minor revisions that should also be considered;
1 - The data description states "of 110,975 biogas plants been established in China, including 6,737 large-scale and 34 extra large-scale biogas plants" but then goes on to describe the 56 they selected as "full-scale", does that mean those 56 are large or extra-large?
2 - (line 133)

It would be nice to have the gel images for "The integrity of DNA extracts was checked on 0.7% (w/v) agarose gel with GelRed nucleic acid gel stain".
3 - (line 163)
"The Illumina raw reads were cleaned by trimming the adapter sequences and low-quality regions using two in-house software clean_adapter and clean_lowqual [14]"
ref[14] =Clean_adapter and clean_lowqul on github. https://github.com/fanagislab/DBG_assembly/tree/master/clean_illumina
This github link gives an ERROR 404, we need access to this, and some indication of its suitability to the task, why was an inhouse script written instead of using an existing tool?
4 - (line 194)
"The rarefaction curve approached saturation with the increase of sample number (Fig. 1a), suggesting that our gene catalog covered the vast majority of microbial genes in full-scale BGPs."
This is a little misleading, the saturation of sampling is not indicative of the level of coverage of all BGPs, only that you have captured the majority of genes present within your sampled BGPs. You should adjust the sentence to:
"The rarefaction curve approached saturation with the increase of sample number (Fig. 1a), suggesting that our gene catalog covered the vast majority of microbial genes to be found in the 56 full-scale BGPs sampled in this study."
More comparison to existing datasets would need to be done to evaluate the coverage of the catalog for all BGPs. Even the one example that has been compared to, shows that ~40% of that comparison dataset are not present in the catalog.
5 - (Line 263)
"represented by 400 genera, 6,816 KOs (Additional file 5: Fig. S4), accounting for about 98.76% and 99.39% of the total relative abundance of annotated genera and KOs"
This sentence doesn't make sense to me, please clarify its meaning.
6 - Fig 5a - Why were "Other" category BGPs excluded from this analysis?
7 - (line 281)
Section on "Microbial functional differentiation among BGPs with different feedstocks"
This section is also not required as part of a datanote describing the data as it goes into the analysis of the data, it could be removed entirely.
As it it this section is lacking in any statistical evaluation and the discussion makes bold statements about some groups having higher/lower relative abundance of things that when you look at the Fig5 are not at all obvious with the interquartile ranges overlapping considerably. e.g. "the relative abundance of genes involved in the hydrolysis of proteins was much higher in MCH (Fig. 5c)," Fig 5c shows an increase median abundance value for MCH, but all 3 have drastically overlapping IQRs. Some indication of the significance or robustness of these findings would be appropriate. Please also see my previous comment on the suitability of the abundance calculations and normalisation.
8 - If analysis of the data is to be included, another point that should be addressed is that of the pH of the plants. One of the major environmental influences on bacterial activity in anaerobic digestion (AD) is pH (See here for ref 10.1186/1754-6834-5-39.), the pH of the reactors are recorded in the metadata, but there has been no mention of its effects within the discussion, how does the pH correlate with the 4 reactor classes MCA (13 cattle manure BGPs), MCH (6 chicken manure BGPs), MPI (27 pig manure BGPs),

and OTH (10 BGPs with other substrates)? How does pH effect beta diversity of samples? etc...

Level of Interest

Please indicate how interesting you found the manuscript: Choose an item.

Quality of Written English

Please indicate the quality of language in the manuscript: Choose an item.

Declaration of Competing Interests

Please complete a declaration of competing interests, considering the following questions:

- Have you in the past five years received reimbursements, fees, funding, or salary from an organisation that may in any way gain or lose financially from the publication of this manuscript, either now or in the future?
- Do you hold any stocks or shares in an organisation that may in any way gain or lose financially from the publication of this manuscript, either now or in the future?
- Do you hold or are you currently applying for any patents relating to the content of the manuscript?
- Have you received reimbursements, fees, funding, or salary from an organization that holds or has applied for patents relating to the content of the manuscript?
- Do you have any other financial competing interests?
- Do you have any non-financial competing interests in relation to this paper?

If you can answer no to all of the above, write 'I declare that I have no competing interests' below. If your reply is yes to any, please give details below.

I declare that I work for GigaScience as a data curator.

I agree to the open peer review policy of the journal. I understand that my name will be included on my report to the authors and, if the manuscript is accepted for publication, my named report including any attachments I upload will be posted on the website along with the authors' responses. I agree for my report to be made available under an Open Access Creative Commons CC-BY license (http://creativecommons.org/licenses/by/4.0/). I understand that any comments which I do not wish to be included in my named report can be included as confidential comments to the editors, which will not be published.

Choose an item.

To further support our reviewers, we have joined with Publons, where you can gain additional credit to further highlight your hard work (see: https://publons.com/journal/530/gigascience). On publication of this paper, your review will be automatically added to Publons, you can then choose whether or not to claim your Publons credit. I understand this statement.

Yes Choose an item.
