# Supplementary material for: A microbial gene catalog of anaerobic digestion from full-scale biogas plants
Source: Gigascience. 2021 Jan 27;10(1):giaa164. doi: 10.1093/gigascience/giaa164 (PMC7842101; doi:10.1093/gigascience/giaa164)
Supplement: giaa164_Reviewer_1_Report_Revision_1 — Christopher Hunter, Ph.D. -- 11/13/2020 Reviewed [file giaa164_reviewer_1_report_revision_1.pdf]

Reviewer Report

Title: A microbial gene catalog of anaerobic digestion from full-scale biogas plants

Version: Revision 1 **Date:** 11/13/2020

Reviewer name: Christopher Hunter, Ph.D.

Reviewer Comments to Author:

I would like to thank the authors for addressing all of my points in there response to reviewers document this made the process of rereview much easier.
I can confirm that the revisions do address all of my concerns and I am happy to now recommend this manuscript for acceptance.
The document would benefit from a fresh set of eyes checking over the wording and grammar and I list here a couple of examples that could be made easier to read/understand:
(line 55)
"In the context of global climate change, biogas as a renewable energy form has become increasingly attractive to the world's attention in recent years." - > "In the context of global climate change, biogas as a renewable energy form has increasingly drawn the world's attention in recent years.
(line 134)
The integrity of DNA extracts was checked on 0.7% (w/v) agarose gel with GelRed nucleic acid gel stain (cat. no. 41003; Biotium, USA), and DNA samples with obvious concentrated DNA band and the fragment length of the band > 15 kb were used for further analysis (Additional file 3: Fig S2).
->
The integrity of DNA extracts was checked on 0.7% (w/v) agarose gel with GelRed nucleic acid gel stain (cat. no. 41003; Biotium, USA). DNA samples showing obvious concentrated DNA bands >15 kb in size were used for further analysis (Additional file 3: Fig S2).

Level of Interest

Please indicate how interesting you found the manuscript: Choose an item.

Quality of Written English

Please indicate the quality of language in the manuscript: Choose an item.

Declaration of Competing Interests

Please complete a declaration of competing interests, considering the following questions:

- Have you in the past five years received reimbursements, fees, funding, or salary from an organisation that may in any way gain or lose financially from the publication of this manuscript, either now or in the future?
- Do you hold any stocks or shares in an organisation that may in any way gain or lose financially from the publication of this manuscript, either now or in the future?
- Do you hold or are you currently applying for any patents relating to the content of the manuscript?
- Have you received reimbursements, fees, funding, or salary from an organization that holds or has applied for patents relating to the content of the manuscript?
- Do you have any other financial competing interests?
- Do you have any non-financial competing interests in relation to this paper?

If you can answer no to all of the above, write 'I declare that I have no competing interests' below. If your reply is yes to any, please give details below.

I am employed by GigaScience as a database curator for GigaDB.

I agree to the open peer review policy of the journal. I understand that my name will be included on my report to the authors and, if the manuscript is accepted for publication, my named report including any attachments I upload will be posted on the website along with the authors' responses. I agree for my report to be made available under an Open Access Creative Commons CC-BY license (http://creativecommons.org/licenses/by/4.0/). I understand that any comments which I do not wish to be included in my named report can be included as confidential comments to the editors, which will not be published.

Choose an item.

To further support our reviewers, we have joined with Publons, where you can gain additional credit to further highlight your hard work (see: https://publons.com/journal/530/gigascience). On publication of this paper, your review will be automatically added to Publons, you can then choose whether or not to claim your Publons credit. I understand this statement.

Yes Choose an item.
