# Supplementary material for: A microbial gene catalog of anaerobic digestion from full-scale biogas plants
Source: Gigascience. 2021 Jan 27;10(1):giaa164. doi: 10.1093/gigascience/giaa164 (PMC7842101; doi:10.1093/gigascience/giaa164)
Supplement: giaa164_Reviewer_2_Report_Original_Submission — James P. J. Chong -- 8/5/2020 Reviewed [file giaa164_reviewer_2_report_original_submission.pdf]

Reviewer Report

Title: A microbial gene catalog of anaerobic digestion from full-scale biogas plants

Version: Original Submission **Date:** 8/5/2020

Reviewer name: James P. J. Chong

Reviewer Comments to Author:

This manuscript reports the metagenomic sequencing of a large number of anaerobic digestion (AD) plants in China.
The methods for carrying out this work are appropriate and generally well described. However there are a few points that should be addressed:
- It is not clear what the rationale or effect of the freeze thaw steps were on DNA extraction
- There is no mention of what the quality criteria were that DNA samples had to pass in order to be sequenced
- There do not appear to be biological replicates of the samples, so it is not clear how representative the samples are of each digester
- It is not clear whether technical replicates of DNA extraction were carried out, or how consistent this process was

I'm not sure I agree with some of the conclusions:
- The authors claim this is a "comprehensive microbial gene catalog of anaerobic digestion" (line 320), but Fig S2 shows that their dataset does not include 34% of a related, but smaller dataset. Surely if these genes are missing from the current work then this cannot be "comprehensive"? It could perhaps be claimed to be "comprehensive for AD plants located in China", although with an N50 of ~ 4 kb and 56% of genes reported by the authors as less than full length, this is also perhaps overstating their claim.
- The AD core genera claim is difficult to substantiate without showing that the genera identified are different from those found for the core genera in the feedstock i.e. those found in cattle, chicken and pig gut microbiomes. It would be good to add such a comparison using published data.

The quality of the language in the manuscript is generally good.
Statistics are appropriately used.
Additional clarification would be useful where the authors claim 56 "full-scale" biogas plants were sampled, but don't define what "full-scale" represents. They mention "large" and "extra large" scales, but none of these terms are defined with respect to volume. Samples are taken from digesters ranging from 12 m3 to 8000 m3. If 12 m3 is considered "full scale" (this is not the case in many parts of the world), what range of reactors are "large scale" compared to "extra large" scale?
There is little operational information concerning the digesters from which the metagenomes were sequenced. There is no indication of hydraulic retention time, feedstock composition (e.g. COD), or biogas volume / composition, which means little biological insight is possible, as it is impossible to correlate how well each digester was functioning and therefore how well the metagenome associated with each digester was performing. It could be that many of the species identified do not contribute to AD but are rather competing for resources within the digesters, or not metabolically active at all.

Level of Interest

Please indicate how interesting you found the manuscript: Choose an item.

Quality of Written English

Please indicate the quality of language in the manuscript: Choose an item.

Declaration of Competing Interests

Please complete a declaration of competing interests, considering the following questions:

- Have you in the past five years received reimbursements, fees, funding, or salary from an organisation that may in any way gain or lose financially from the publication of this manuscript, either now or in the future?
- Do you hold any stocks or shares in an organisation that may in any way gain or lose financially from the publication of this manuscript, either now or in the future?
- Do you hold or are you currently applying for any patents relating to the content of the manuscript?
- Have you received reimbursements, fees, funding, or salary from an organization that holds or has applied for patents relating to the content of the manuscript?
- Do you have any other financial competing interests?
- Do you have any non-financial competing interests in relation to this paper?

If you can answer no to all of the above, write 'I declare that I have no competing interests' below. If your reply is yes to any, please give details below.

I declare that I have no competing interests

I agree to the open peer review policy of the journal. I understand that my name will be included on my report to the authors and, if the manuscript is accepted for publication, my named report including any attachments I upload will be posted on the website along with the authors' responses. I agree for my report to be made available under an Open Access Creative Commons CC-BY license (http://creativecommons.org/licenses/by/4.0/). I understand that any comments which I do not wish to be included in my named report can be included as confidential comments to the editors, which will not be published.

Choose an item.

To further support our reviewers, we have joined with Publons, where you can gain additional credit to further highlight your hard work (see: https://publons.com/journal/530/gigascience). On publication of

this paper, your review will be automatically added to Publons, you can then choose whether or not to claim your Publons credit. I understand this statement.

Yes Choose an item.
