# Supplementary material for: A microbial gene catalog of anaerobic digestion from full-scale biogas plants
Source: Gigascience. 2021 Jan 27;10(1):giaa164. doi: 10.1093/gigascience/giaa164 (PMC7842101; doi:10.1093/gigascience/giaa164)
Supplement: giaa164_Reviewer_3_Report_Original_Submission — KornÃ©l L. KovÃ¡cs -- 8/17/2020 Reviewed [file giaa164_reviewer_3_report_original_submission.pdf]

Reviewer Report

Title: A microbial gene catalog of anaerobic digestion from full-scale biogas plants

Version: Original Submission **Date:** 8/17/2020

Reviewer name: KornÃ©l L. KovÃ¡cs

Reviewer Comments to Author:

The paper describes an impressively large sequencing project involving the microbiome of 59 full scale biogas facilities. The collection of more than 22 thousand AD genes is useful for further research, this justifies the work done. However, the paper draws disappointingly poor conclusions at the end of the data analysis. There is no innovative, "take home", new message for the readers. The only aspect discussed briefly is the clustering of the metagenomes according to the employed AD substrate, which is not surprising or novel in AD microbiology at all. If this is indeed the only conclusion that the authors could extract from this work, the paper is not suitable and not acceptable for publication in GigaScience, which expects novel ideas, discoveries to be communicated from the metagenomic studies. The paper needs very thorough revision before it can be considered for publication in GIGA.

Specific major comments:

1. You should check if a number of other process parameters, e.g. reactor size and geometry, mixing, additional substrate biomasses, residence times, loading rates, biogas yields, product methane contents, etc. would correlate with the metagenom data. Alterations caused by feeding the AD reactors with various types of animal manure is trivial and expectable. Did you screen co-fermentation systems?
2. You should compare your data with literature data more extensively regarding the results obtained by others using sequencing and bioinformatics methods, substrates, geographical sites, temperatures, etc. There have been numerous relevant studies published in Europe (Germany, Austria, Hungary) to consider.
3. In the bioinformatics workflow you should compare results obtained by using reference databases other than NCBI-NR.
4. Similarly, it is a major flaw that genome-based evaluation (binning) of the data is not included. This would validate the read-based bioinformatics. In addition, binning would allow you species level resolution of the microbiota, which could be more informative than looking at the microbiomes at genus level.

Additional corrections needed:

1. L.56. There is no substantial "shortage" of fossil fuels. It is the environmental global climate change effect that drives research into renewable.
2. L.62. How do you calculate "over the last two decades"? Industrial biogas technology older than that.
3. L.67-69. Adjust the tenses of the verbs.
4. L.80-81. There have been many more studies on large scale AD microbiomes.
5. L.87-88. L. 89. Grammar!
6. L.93. "Ambient" temperature varies probably a great deal across China. What are the real values?

7. L.198. "most of the genes were shared"… Fig 1.b. does not corroborate this statement.
8. L.199. Which are "widely existed" genes?
9. L.269. What justifies the presence of "more than 80%" as a core microbiome member? Core microbiome means the collection of microbes present in ALL investigated samples.
10. L.304 and 307. The manures of the various animals is rich in these substances because their FEED is different from each other!
11. L.329. "consortium of …genes". Genes do not form a consortium.

Level of Interest

Please indicate how interesting you found the manuscript: Choose an item.

Quality of Written English

Please indicate the quality of language in the manuscript: Choose an item.

Declaration of Competing Interests

Please complete a declaration of competing interests, considering the following questions:

- Have you in the past five years received reimbursements, fees, funding, or salary from an organisation that may in any way gain or lose financially from the publication of this manuscript, either now or in the future?
- Do you hold any stocks or shares in an organisation that may in any way gain or lose financially from the publication of this manuscript, either now or in the future?
- Do you hold or are you currently applying for any patents relating to the content of the manuscript?
- Have you received reimbursements, fees, funding, or salary from an organization that holds or has applied for patents relating to the content of the manuscript?
- Do you have any other financial competing interests?
- Do you have any non-financial competing interests in relation to this paper?

If you can answer no to all of the above, write 'I declare that I have no competing interests' below. If your reply is yes to any, please give details below.

I declare that I have no competing interests

I agree to the open peer review policy of the journal. I understand that my name will be included on my report to the authors and, if the manuscript is accepted for publication, my named report including any attachments I upload will be posted on the website along with the authors' responses. I agree for my report to be made available under an Open Access Creative Commons CC-BY license

(http://creativecommons.org/licenses/by/4.0/). I understand that any comments which I do not wish to be included in my named report can be included as confidential comments to the editors, which will not be published.

Choose an item.

To further support our reviewers, we have joined with Publons, where you can gain additional credit to further highlight your hard work (see: https://publons.com/journal/530/gigascience). On publication of this paper, your review will be automatically added to Publons, you can then choose whether or not to claim your Publons credit. I understand this statement.

Yes Choose an item.
